# Gut microbiota regulate atherosclerosis via the gut-vascular axis: a scoping review of mechanisms and therapeutic interventions

**DOI:** 10.3389/fmicb.2025.1606309

**Published:** 2025-08-08

**Authors:** Dezhi Zhang, Xiaoqing He, Yewei Shi, Xinyue Chen, Kai Yu, Shuang Wang

**Affiliations:** ^1^Department of General Practice, The First Hospital of China Medical University, Shenyang, China; ^2^Department of Orthopedics and Traumatology, Liaoning University of Traditional Chinese Medicine, Shenyang, China

**Keywords:** atherosclerosis, gut microbiota, gut-vascular axis, metabolites, pathogenesis, treatment strategies

## Abstract

**Background:**

Atherosclerosis (AS) is a chronic inflammatory and metabolic disease, and advancements in its treatment have long been hampered by the complexity of its underlying mechanisms. The newly proposed “gut-vascular axis” theory holds promise for systematically elucidating the interactions between gut microbiota (GM) and vascular homeostasis. This provides a novel research framework for formulating precise preventive and therapeutic strategies against AS.

**Objective:**

To explore the mechanisms by which GM and their metabolites regulate AS via the gut-vascular axis, and the potential application of intervention strategies targeting this axis in the prevention and treatment of AS.

**Methods:**

Following the methods of a scoping review, we searched the databases Cochrane Library, Embase, PubMed and Web of Science, and the search period was from January 1, 2014, to July 25, 2024. Two researchers individually reviewed the basic characteristics of the included studies.

**Results:**

A total of articles were retrieved, identified 3556 articles and 192 of which were ultimately included in the study. The results are divided into three main sections, including the impact of GM and gut microbial metabolites (GMMs) on AS, and how various intervention factors can influence AS by influencing the composition of the GM.

**Conclusion:**

Based on the gut-vascular axis theory, a multi-target combined intervention strategy has been proposed, encompassing probiotics/prebiotics regulation and precise nutritional interventions, thereby establishing novel approaches for AS prevention and treatment. Future research should prioritize the integration of artificial intelligence (AI) with multi-omics technologies to comprehensively decipher the interaction mechanisms between microbial metabolic networks and vascular pathologies within the gut-vascular axis framework. This interdisciplinary approach will promote the advancement of AS management toward emerging personalized precision medicine.

**Systematic review registration:**

10.17605/OSF.IO/X8WQM.

## 1 Introduction

Coronary artery disease (CAD), peripheral arterial disease (PAD), stroke, and other illnesses are all included in the category of atherosclerotic diseases (Tsao et al., 2022). According to the 2019 Global Burden of Disease (GBD) data, from 1990 to 2019, the number of cardiovascular disease (CVD) cases and incidents increased from 272 million to 523 million, the number of deaths rose from 12.1 million to 18.6 million, and disability-adjusted life years (DALYs) increased from 279.8 million to 393.1 million, highlighting the growing burden of CVD worldwide (Roth et al., 2020). AS is a chronic inflammatory illness that serves as the pathological foundation of CVD, characterized by the accumulation of fat or fibrous substances in the arterial intima, which gradually invades the artery cavity, obstructs blood flow, and ultimately results in tissue ischemia caused by atherosclerotic plaque (Libby et al., 2019; Tsao et al., 2022). AS increases the risk of atrial fibrillation, cardiomyopathy, gangrene, ischemic episodes, myocardial infarction, stable angina pectoris, stroke and ulceration (Libby et al., 2019). Numerous research investigations have shown that conventional variables, such as age, dyslipidemia, hyperglycemia, hypertension, obesity and sex, and non-traditional factors, including air pollution, changes in the microbiome, clonal hematopoiesis, fetal programming and sleep disorders, contribute to decreased vascular tone, enhanced inflammation, increased shear stress and vascular permeability, ultimately leading to impaired endothelial function (Benincasa et al., 2022; Cimmino et al., 2023). Comorbid conditions such as arterial hypertension, dyslipidemia, endothelial dysfunction, type 2 diabetes mellitus (T2DM) and visceral adiposity contribute to the development of an atherogenic environment (Lechner et al., 2020). Immune cells originating in the spleen contribute to atherosclerotic plaque inflammation and the inflammatory response to myocardial infarction (Heusch and Kleinbongard, 2025). The spleen becomes a major organ for extramedullary myelopoiesis, contributing to an already elevated pool of circulating Ly6Chi monocytes that would readily enter the inflamed arteries to accelerate AS (Dutta et al., 2012). The induction of paracrine and autocrine interactions among various cell types, including vascular smooth muscle cells, endothelial cells, monocytes/macrophages, dendritic cells, and T cells, leads to the production of microRNAs and tissue-specific epigenetic reprogramming of these cells (Panduga et al., 2024). This reprogramming is regulated by DNA methylation and histone modifications, which ultimately contributing to the development of AS (Panduga et al., 2024). Macrophages play a crucial role in the development of CVD by promoting AS and contributing to plaque vulnerability (Jinnouchi et al., 2020; Liu D. et al., 2022). Macrophages within the plaque are a heterogenous population known to be derived from a number of sources, consisting of both true macrophages and macrophage-like cells, which may contribute differently to lesion development or regression (Susser and Rayner, 2022; Fang et al., 2023). A study has demonstrated that oxidized phospholipids, which are prevalent in atherosclerotic lesions, can precipitate apoptosis and destabilization of regulatory T (Treg) cells (Appleton et al., 2023). The resultant dysfunction of these cells is a pivotal factor in the advancement of AS (Appleton et al., 2023).

The gut microbiota (GM) contains approximately 3 × 10^13^ bacteria, most of which are symbiotic which are composed of *Actinobacteria*, *Bacteroidetes*, *Fusobacteria*, *Firmicutes*, *Proteobacteria*, and *Verrucomicrobia* (Goodrich et al., 2014). The “gut-vascular axis” theory proposed by Lorenzo’s team provides a novel research paradigm for elucidating the molecular mechanisms through which GM and their metabolites regulate the development and progression of AS (Flori et al., 2024). GM and gut microbial metabolites (GMMs) directly or indirectly influence vascular inflammation, endothelial function, and lipid metabolism (LM) through complex host-microbe interactions, thereby contributing to AS progression (Alexandrescu et al., 2024; Mao et al., 2024). Trimethylamine N-oxide (TMAO) promotes the formation and instability of atherosclerotic plaques by inducing macrophage foam cell formation, activating the NLRP3 inflammasome, and enhancing platelet activation (Zhen et al., 2023; Dolkar et al., 2024). In contrast, short-chain fatty acids (SCFAs), such as butyrate, exert anti-atherosclerotic effects via multiple pathways, including activation of GPR41/43 to suppress vascular inflammation, enhancement of intestinal barrier integrity, and promotion of Treg cell differentiation (Manolis et al., 2022; Alexandrescu et al., 2024; Singh et al., 2024). Notably, SCFAs inhibit hepatic flavin monooxygenase 3 (FMO3) expression by suppressing histone deacetylases (HDACs), thereby reducing TMAO production (Alexandrescu et al., 2024; Gan et al., 2024b). This highlights the importance of the SCFAs-TMAO balance as a pivotal regulatory node in AS (Alexandrescu et al., 2024; Gan et al., 2024b). Therapeutic strategies targeting the gut-vascular axis show promise, including probiotics/prebiotics to remodel microbial composition, TMA lyase inhibitors to block TMAO generation, and dietary interventions (increased fiber intake) to boost SCFAs synthesis (Cai et al., 2022; Chen L. et al., 2023; Jarmukhanov et al., 2024). Furthermore, microbiome-directed therapies, such as fecal microbiota transplantation (FMT), have demonstrated efficacy in animal models by reducing pathogenic bacterial abundance and TMAO levels, thereby attenuating atherosclerotic lesions (Gan et al., 2024b; Zhang et al., 2025). These findings provide a theoretical foundation and translational avenues for developing novel interventions for AS.

This scoping review integrates a multidimensional evidence system to elucidate the mechanisms underlying the interaction between GM and AS within the theoretical framework of the “gut-vascular axis”. Through systematic literature retrieval and comprehensive analytical methodologies, this study elucidated the influence of GM and its metabolites, specifically SCFAs and TMAO, on the pathogenesis of AS. This is achieved through molecular pathways related to immunometabolism, LM, intestinal barrier homeostasis, and regulation of vascular endothelial function. The translational value of microbiota-targeted interventions directed at the gut-vascular axis for AS prevention and treatment is critically evaluated. This synthesis advances the theoretical development of the gut-vascular axis paradigm and establishes a foundation for precision prevention strategies based on gut-vascular axis modulation. These findings hold significant scientific implications for optimizing cardiovascular disease prevention frameworks and promoting clinical translation, offering novel approaches to address the global challenge of cardiovascular disease burden.

## 2 Materials and methods

### 2.1 Protocol and registration

This scoping review was based on the methodological guidelines developed by Arksey and O’Malley and subsequent refinements by Colquhoun et al., and adheres to the PRISMA Extension for Scoping Reviews (PRISMA-ScR): Checklist and Explanation.(Arksey and O’Malley, 2005; Colquhoun et al., 2014; Tricco et al., 2018). The protocol for this review is available within the Open Science Framework -Registration doi: https://doi.org/10.17605/OSF.IO/X8WQM.

### 2.2 Inclusion and exclusion criteria

Inclusion Criteria: 1. Articles that aim to investigate the mechanisms by which GM and GMMs affect AS, as well as those that explore the treatment of AS through the modulation of GM and GMMs. 2. Original Research Article. 3. Published from 2019 to 2024. Exclusion Criteria: 1. Articles not related to the research topic. 2. The article only includes *in vitro* experiments. 3. These articles do not describe in detail the mechanism of GM and GMMs or the intervention methods affecting AS. 4. Articles of types such as these, conference abstracts, books, reviews, guidelines, news articles, etc. 5. Articles for which full text is not available. 6. Mendelian randomization study.

### 2.3 Databases searched AND search strategy

The Cochrane Library, Web of Science, Embase, and PubMed were among the electronic resources that were searched the search period was from January 1, 2014, to July 25, 2024. Search terms were used to query titles, abstracts, and keywords in the databases. The search terms included: “atherosclerotic cardiovascular,” “atherosclerosis”, “gastrointestinal microbiome,” “gut microbiota,” “intestinal microbiome,” “gut microbiome,” “intestinal microbiota,” “gastrointestinal microbial communities” (Supplementary Table 1). Using PubMed as an example, as Table 1 illustrates.

**TABLE 1:** Search Strategies for PubMed Database.

Search strategy	Searches	Number of obtained literature
#1	(((((“Gastrointestinal Microbiome” [Title/Abstract]) OR (“gut microbiota” [Title/Abstract])) OR (“Intestinal microbiome”[Title/Abstract])) OR (“gut microbiome”[Title/Abstract])) OR (“Intestinal microbiota” [Title/Abstract])) OR (“Gastrointestinal Microbial Communities” [Title/Abstract]) Sort by Publication Date	74,315
#2	(((((“Gastrointestinal Microbiome” [Title/Abstract]) OR (“gut microbiota” [Title/Abstract])) OR (“Intestinal microbiome” [Title/Abstract])) OR (“gut microbiome” [Title/Abstract])) OR (“Intestinal microbiota”[Title/Abstract])) OR (“Gastrointestinal Microbial Communities” [Title/Abstract]) Filters: from 2014/1/1-2024/7/25 Sort by: Publication Date	70,236
#3	(“atherosclerotic cardiovascular disease” [Title/Abstract]) OR (“atherosclerosis” [Title/Abstract]) Sort by Publication Date	148,042
#4	(“atherosclerotic cardiovascular disease” [Title/Abstract]) OR (“atherosclerosis” [Title/Abstract]) Filters:from 2014/1/1-2024/7/25 Sort by: Publication Date	67,256
#5	((((((“Gastrointestinal Microbiome” [Title/Abstract]) OR (“gut microbiota” [Title/Abstract])) OR (“Intestinal microbiome” [Title/Abstract])) OR (“gut microbiome” [Title/Abstract])) OR (“Intestinal microbiota” [Title/Abstract])) OR (“Gastrointestinal Microbial Communities” [Title/Abstract]) AND (2014/1/1:2024/7/25[pdat])) AND ((atherosclerotic cardiovascular disease [Title/Abstract]) OR (“atherosclerosis” [Title/Abstract]) AND (2014/1/1:2024/7/25 [pdat])) Filters:from 2014/1/1-2024/7/25 Sort by: Publication Date	905

### 2.4 Selection of sources of evidence

We imported the literature into EndNote (version 21.4) for subsequent screening, after removing duplicates. The studies were independently verified by two researchers using the inclusion and exclusion criteria. Initially, we conducted a literature review that focused on the titles and abstracts of studies to identify those that were ineligible. Then, we obtained the full texts of the initially screened studies. Finally, we performed a secondary screening of the full texts according to the inclusion and exclusion criteria to select the final set of included studies. A third researcher will be consulted in order to settle any disputes that may arise between the two researchers during this process.

### 2.5 Data extraction and synthesis

Data were independently extracted by one author and verified by another. Discrepancies were discussed and resolved. The Strengthening the Organization and Reporting of Microbiome Studies (STORMS) checklist is a comprehensive 17-item reporting guideline developed by a multidisciplinary team of experts (Mirzayi et al., 2021). It is designed to provide standardized reporting frameworks for human microbiome studies, thereby enhancing the rigor, reproducibility, and transparency of the research design (Mirzayi et al., 2021). Additionally, it supports manuscript preparation, peer review processes, and comparative analysis of results (Mirzayi et al., 2021). This article identifies essential information to be extracted from the included studies based on the SCOPING REVIEW methodology and GM’s STORMS framework. The basic information of the included studies (such as authors, publication year, etc.), descriptive details of the study design (including species, animal sex, sequencing methods, etc.), research objectives, results, and conclusions were extracted. Before synthesizing the findings, the studies were grouped according to their primary research objectives and factors.

### 2.6 Data analysis

We used Endnote 21.4 software to screen and manage the literature included in the study. Then use GraphPad Prism version 9 (GraphPad Prism software) to create stacked bar charts. Create multiple types of bubble point charts and two column line charts on the websit.^1^ Draw flowcharts and mechanism diagrams on the BioRender website.^2^

## 3 Result

### 3.1 Characteristics of reviewed studies

**FIGURE 1:**
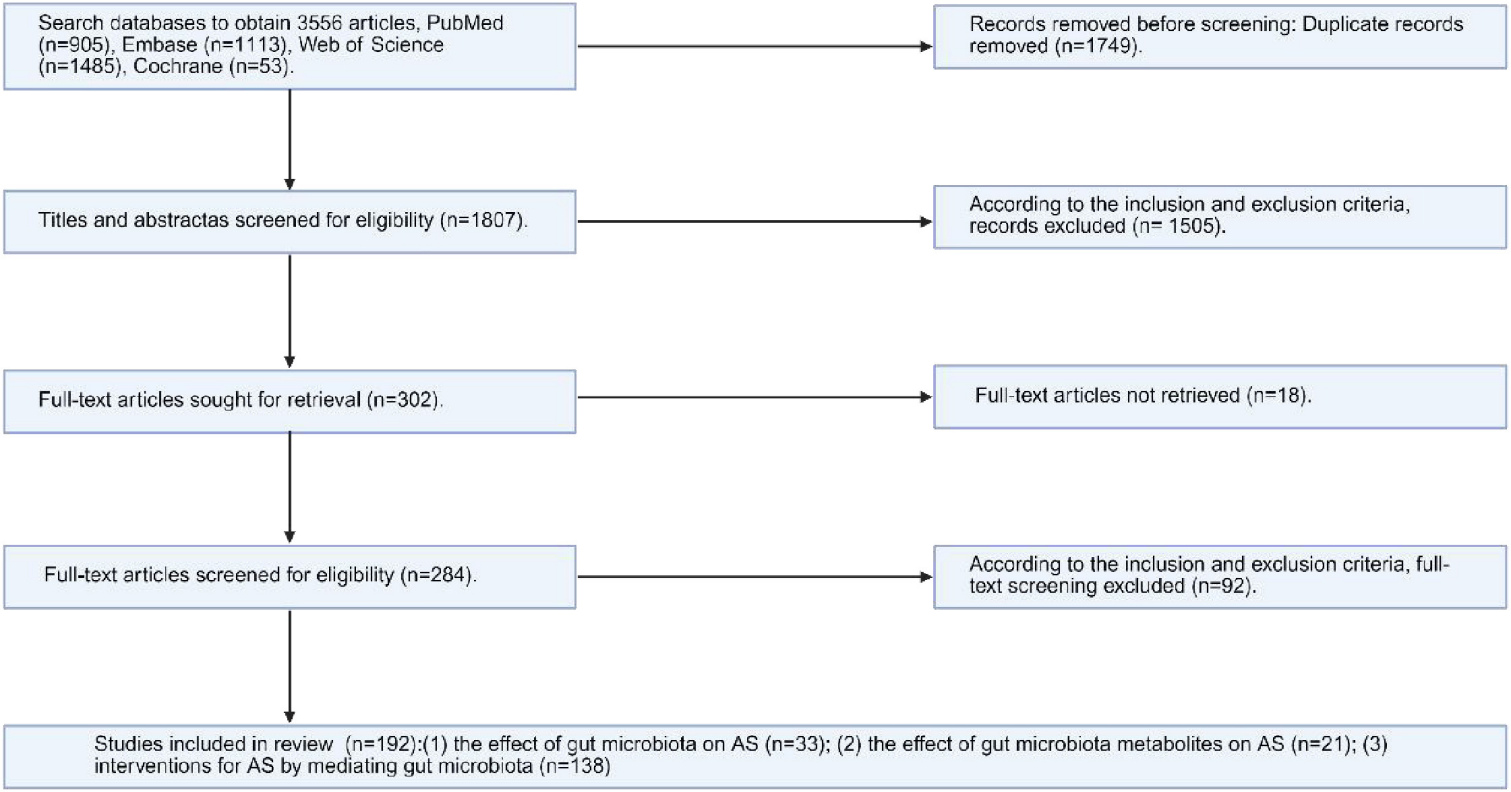
Flowchart of the inclusion and exclusion of participants in this study. Created in BioRender. Dezhi (2025) (https://BioRender.com/q30c258). Detailed information is available in Supplementary material 1.

The search strategy identified 3,556 articles, and after deleting duplicate articles, 1,807 remained. After applying the inclusion and exclusion criteria and reading the titles and abstracts, 1,505 articles were excluded, leaving 302 that needed to be retrieved in full (Figure 1 and Supplementary material 1). Eighteen articles could not be retrieved owing to insufficient information. After reviewing the full texts of the remaining 284 articles, 192 studies were included (Brandsma et al., 2019; Dong et al., 2019; Gautam et al., 2019; Han et al., 2019; He et al., 2019; Kiouptsi et al., 2019; Koeth et al., 2019; Luo et al., 2019; Millar et al., 2019; Moghadasian et al., 2019; Nie et al., 2019; Shi et al., 2019; Tuomisto et al., 2019; Wil et al., 2019; Wu F. et al., 2019; Wu F. et al., 2019; Xiong et al., 2019; Zhao et al., 2019; Aldana-Hernández et al., 2020; Carnevale et al., 2020; Chen et al., 2020; Ding et al., 2020; Guo et al., 2020; Hassan et al., 2020; Ji et al., 2020; Kappel et al., 2020; Kwun et al., 2020; Li et al., 2020; Liang et al., 2020; Liu C. et al., 2022; Liu Q. et al., 2020; Loffredo et al., 2020; Millar et al., 2020; Toya et al., 2020; Wang et al., 2020; Wu et al., 2020; Yue et al., 2020; Zhu et al., 2020; Baragetti et al., 2021; Chen et al., 2021; Garshick et al., 2021; Ghosh et al., 2021; Gu et al., 2021; Guo et al., 2021; Hu et al., 2021; Huang et al., 2021; Ji et al., 2021; Jin et al., 2021; Koay et al., 2021; Kobayashi et al., 2021; Krupa et al., 2021; Li X. X. et al., 2021; Li Y. H. et al., 2021; Liang et al., 2021; Liu F. et al., 2021; Liu F. et al., 2021; Lv et al., 2021; Shi et al., 2021; Sun et al., 2021; Szabo et al., 2021; Tang J. et al., 2021; Tang W. H. W. et al., 2021; Tian et al., 2021; Tsutsumi et al., 2021; van den Brule et al., 2021; Wang F. et al., 2021; Wang J. et al., 2021; Xiao et al., 2021; Xie et al., 2021; Xue et al., 2021; Yan et al., 2021; Yang R. et al., 2021; Yang S. et al., 2021; Zhang et al., 2021a; Zhang et al., 2021b; Zhao et al., 2021; Zhu et al., 2021; Bai et al., 2022; Cai et al., 2022; Choroszy et al., 2022; Clark et al., 2022; Collins et al., 2022; Dai et al., 2022; Dong et al., 2022; Dongliang et al., 2022; Gan et al., 2022; Gao H. et al., 2022; Gao M. et al., 2022; Haghikia et al., 2022; Han et al., 2022; Huang W. C. et al., 2022; Huang Y. et al., 2022; Kashyap et al., 2022; Kim et al., 2022; Kwek et al., 2022; Li X. L. et al., 2022; Li Y. W. et al., 2022; Li Y. W. et al., 2022; Lin et al., 2022; Liu C. et al., 2022; Liu D. et al., 2022; Liu J. et al., 2022; Liu Y. J. et al., 2022; Ma et al., 2022; Millar et al., 2022; Nakajima et al., 2022; Panyod et al., 2022; Qi et al., 2022; Qiao et al., 2022; Shan et al., 2022; Shi G. et al., 2022; Shi H. et al., 2022; Shi H. et al., 2022; Sto et al., 2022; Szabo et al., 2022; Wang A. et al., 2022; Wang Y. et al., 2022; Wu et al., 2022; Xie et al., 2022; Xu et al., 2022; Xue et al., 2022; Yang Q. et al., 2022; Yang X. Y. et al., 2022; Zhai et al., 2022; Zhang et al., 2022; Zhen et al., 2022; Zhou et al., 2022; Zhu et al., 2022; Bai et al., 2023; Chen C. Y. et al., 2023; Chen L. et al., 2023; Cheng et al., 2023; Du et al., 2023; Fu et al., 2023; Gan et al., 2023; Hao et al., 2023; He et al., 2023; Huang T. et al., 2023; Hutchison et al., 2023; Jia A. et al., 2023; Jie et al., 2023; Kasahara et al., 2023; Kawamata et al., 2023; Liao et al., 2023; Liu R. et al., 2023; Liu Y. et al., 2023; Lv et al., 2023; Ma et al., 2023; Mu et al., 2023; Panyod et al., 2023; Park et al., 2023; Samuthpongtorn et al., 2023; Sayols-Baixeras et al., 2023; Schönke et al., 2023; Tian et al., 2023; Traughber et al., 2023; Tu et al., 2023; Wang G. et al., 2023; Wang Z. et al., 2023; Wu et al., 2023; Zhang et al., 2023; An et al., 2024; Ding et al., 2024; Gan et al., 2024a; He et al., 2024; Jiang et al., 2024; Jin et al., 2024; Li et al., 2024a; Li et al., 2024b; Li et al., 2024d; Li et al., 2024f; Li et al., 2024g; Liang et al., 2024a; Liang et al., 2024b; Liang et al., 2024c; Luo K. et al., 2024; Ma et al., 2024; Masiá et al., 2024; Miao et al., 2024; Qi et al., 2024; Shen et al., 2024; Sun et al., 2024; Traughber et al., 2024; Wan et al., 2024; Wang X. et al., 2024; Wang Y. et al., 2024; Xing et al., 2024; Xu et al., 2024; Yang et al., 2024; Yu et al., 2024; Yue et al., 2024; Zhu et al., 2024). The study classified the included articles into three distinct research themes based on their respective focus: (1) the impact of GM on AS (*n* = 33); (2) the influence of GMMs on AS (*n* = 21); and (3) interventions for AS through the mediation of GM (*n* = 138). This review compiled a summary table of the key microbial taxa and metabolites associated with AS (Table 2; Supplementary Table 10).

**TABLE 2:** Overall types of AS-associated GM/GMMs.

Feature	AS-associated dysbiosis
Microbial Diversity	Reduced diversity, enrichment of pro-inflammatory species.
*Firmicutes/Bacteroidetes (F/B)* Ratio	Increased *Firmicutes/Bacteroidetes (F/B)* ratio (linked to inflammation).
TMAO-Producing Bacteria	Enrichment of *Lachnoclostridium, Clostridium, Olsenella, Desulfovibrio* (TMAO producers).
TMAO Level	Increased TMAO level.
SCFAs-Producing Bacteria	Decreased *Faecalibacterium, Roseburia*.
SCFAs Levels (Butyrate, Acetate, Propionate)	Decreased SCFAs levels.
Tryptophan derivatives	Decreased 3-indolepropionic acid (IPA) level.
Beneficial bile acids	Decreased Glycine lipids, Glycoursodeoxycholic Acid levels.
Urolithin	Decreased Urolithin A, Urolithin B levels.
Harmful gut microbiota	Increased *Firmicutes, Escherichia coli, Proteobacteria, Deferribacteres*.
Beneficial gut microbiota	Decreased *Blautia, Duncaniella spB8, Blautia producta, Faecalibaculum prausnitzii, Bifidobacterium, Clostridium, Lactobacillus, Streptococcus, Verrucomicrobiota*.

### 3.2 Characteristic of research that were considered in the analysis of the associated GM and AS

To better describe the characteristics of the included studies while considering the level of evidence, we categorized the studies based on whether they involved human subjects or not. We divided the studies into two categories: those including human participants (*n* = 21) and those excluding human participants (*n* = 12). We provided detailed information on these 33 studies based on the identified variables that need to be extracted.

#### 3.2.1 The research includes experiments on human subjects

**FIGURE 2:**
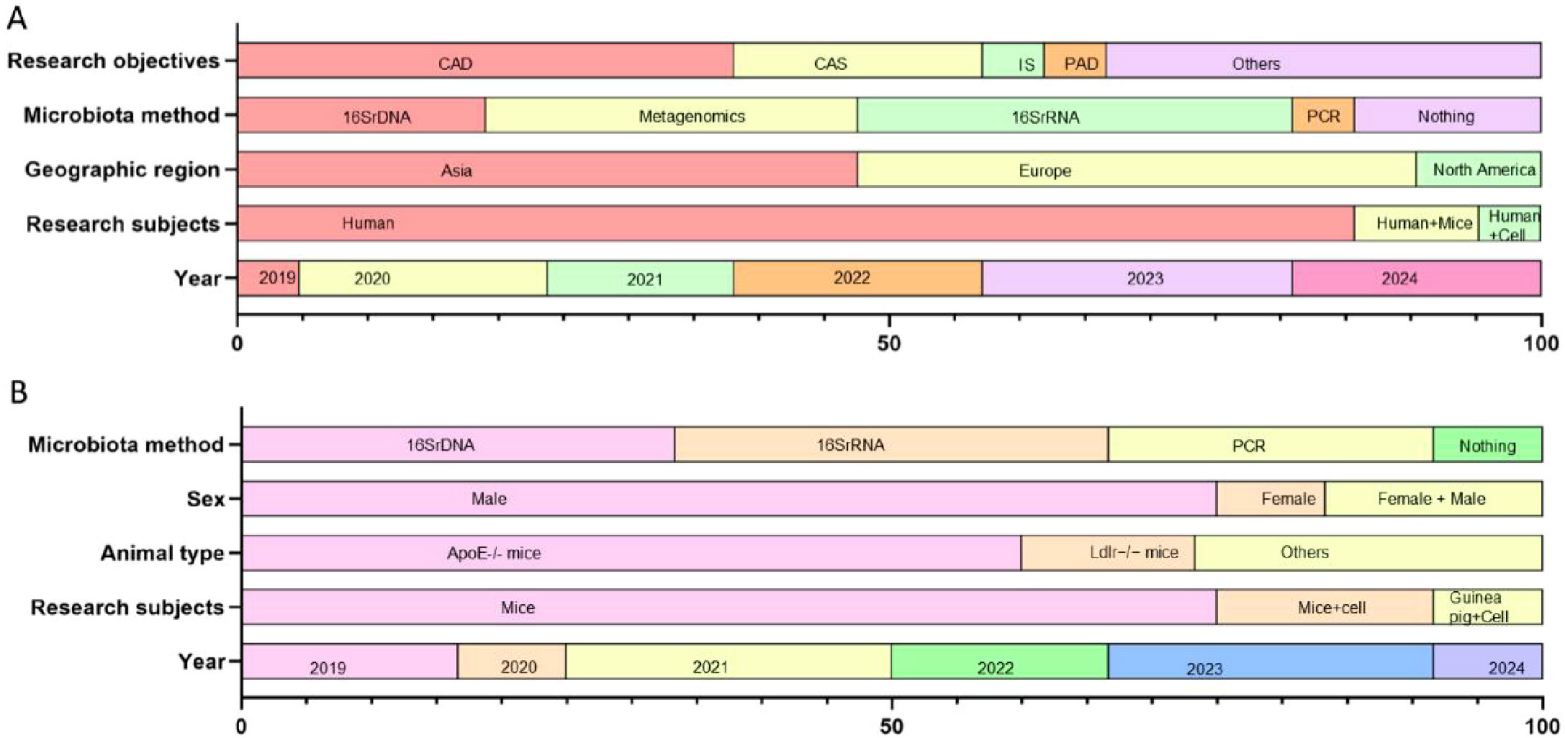
The main characteristics of the effects of GM on AS are presented (*n* =33). **(A)** Bar chart illustrating the main characteristics of studies involving humans (*n* = 21). The data are presented as percentages, reflecting the publication years of the included studies, categories of study subjects, geographical regions of the study populations, research objectives, and primary methods used for measuring gut microbiota. The research objectives were Coronary artery disease (CAD), Carotid atherosclerosis (CAS), Ischemic stroke (IS), Peripheral atherosclerotic disease (PAD), and others (including atherosclerosis and subclinical atherosclerosis). **(B)** Bar chart illustrating the main characteristics of studies not involving humans (*n* = 12). The data are presented as percentages, reflecting the publication years of the included studies, the categories of study subjects, the genotypes of animal models, the sexes of animal models, and the primary methods used for measuring gut microbiota.

This section includes a total of 21 studies (Figure 2A; Supplementary Table 2). The number of studies published in 2019 is the lowest, accounting for 4.76%, while those published in 2023 are the highest, accounting for 23.81%. The studies focusing solely on humans accounted for 85.72%, those involving both humans and mice accounted for *n* = 9.52% and those involving both humans and cells accounted for 4.76%. The study populations are from Asia in 47.62%, Europe in 42.86%, and North America in 9.52%. Since the sequencing methods of GM can affect experimental results, we have compiled the sequencing methods used in these 22 studies. These include 16S rDNA gene sequencing, Metagenomics sequencing, 16S ribosomal RNA gene sequencing, PCR sequencing, and No sequencing (19.05, 28.57, 33.33, 4.76% and *n* = 14.29%, respectively). There are 8 studies examining the impact of GM on CAD, 4 studies investigating the relationship between GM and Carotid Atherosclerosis (CAS), 1 study examining the relationship with Ischemic Stroke (IS), and 1 study looking at the relationship with PAD.

#### 3.2.2 The research includes experiments on animal subjects

This section comprises of 12 studies (Figure 2B; Supplementary Table 3). In terms of publication dates, the number of years with the highest number of publications was 2021 and 2023, each accounted for 25%, while the number of years with the fewest publications was 2020 and 2024, each accounted for 8.33%. 75% of the studies were about mice alone, 16.67% involved mice and cells, and 8.33% involved guinea pigs and cells. Animal experiments require the establishment of animal models of AS, and the success of this model is crucial to experimental outcomes. Therefore, we compiled the common types of animals used to create AS models, including the genotypes of mice: 60% of the mice were Apolipoprotein E-deficient (ApoE-/-), 13.33% were low-density lipoprotein receptor-deficient (Ldlr−/−) mice, and the remaining 26.67% were guinea pigs, hamsters, and animal models lacking known genetic histories linked to AS. Additionally, the sex of the animals may affect the progression of AS, so we also documented the sex of the animals included in the studies: 75% of the animals were male, 8.33% were female, and 16.67% were both male and female. Finally, we compiled the following GM sequencing methods used in the included studies: 16S rDNA gene sequencing and 16S ribosomal RNA gene sequencing accounted for 33.33%, PCR sequencing accounted for 25%, and no sequencing accounted for 8.33%.

### 3.3 Characteristic of research that were considered in the analysis of the associated GMMs and AS

**FIGURE 3:**
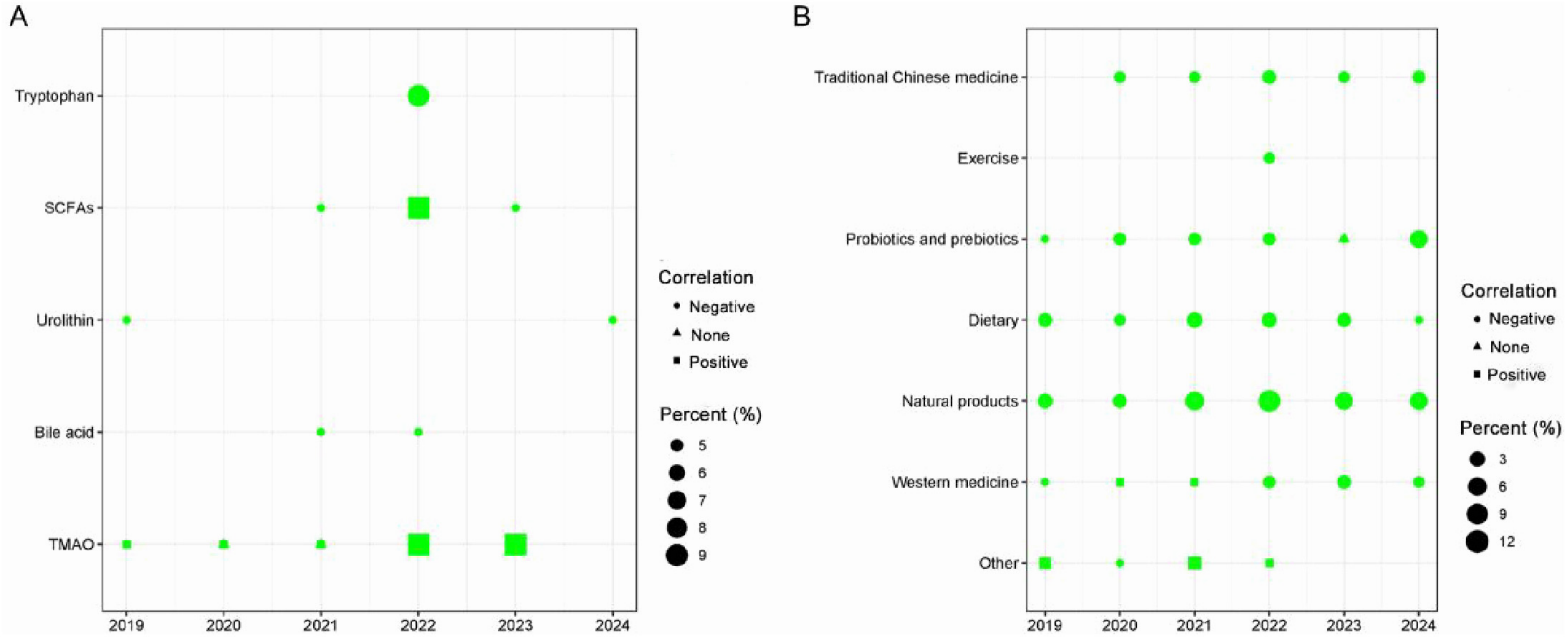
**(A)** The effects of GMMs on AS (*n* = 21), the data are presented as percentages, reflecting the publication years, types of gut microbiota metabolites, and the relationship between these metabolites and AS. **(B)** Factors mediating AS through GM (*n* = 138), the data are presented as percentages, reflecting the publication years, types of intervention factors that can mediate the impact of gut microbiota on AS, and the relationship between these intervention factors and AS.

The types of GMMs included in the reviewed literature consist of TMAO, SCFAs, Tryptophan, Bile acids, and Urolithin, totaling five types (Figure 3A; Supplementary Table 4). Among them, 47.62% of the studies focused on TMAO, followed by SCFAs accounted for 23.81%, and finally Tryptophan, Bile acids, and Urolithin accounted for 9.52%. Of the 21 studies, the highest number of publications was 42.86% in 2022, while the lowest was 4.76% in 2024. Among these studies, 38% reported a positive correlation between GMMs and AS, 42.86% reported a negative correlation, and 19.05% found no association between GMMs and AS.

### 3.4 Characteristic of research that intervention of AS factors by mediating GM

This section is primarily categorized into seven types (Figure 3B; Supplementary Table 5): Probiotics and prebiotics contributed for 15.22%, dietary interventions accounted for 17.39%, natural compounds accounted for 39.86%, Traditional Chinese Medicine (TCM) accounted for 9.42%, Western medicine accounted for 9.42%, exercise accounted for 1.45%, and other factors accounted for 7.25%. The year 2019 observed the least number of studies published, with 9.42%, while 2023 recognized the maximum number of studies published, with 27.54%.

#### 3.4.1 Probiotics and prebiotics

In our investigation of probiotic interventions for AS, we identified that probiotics exert a significantly beneficial impact on AS. This effect is primarily achieved through alterations in GM structure, regulation of microbial metabolites, reduction of systemic inflammatory response (IR), improvement of LM, and maintenance of the stability of the intestinal mucosal barrier (IMB) (Supplementary Table 5; Hassan et al., 2020; Liang et al., 2020; Liu Q. et al., 2020; Kobayashi et al., 2021; Liang et al., 2021; Tang J. et al., 2021; Kashyap et al., 2022; Zhai et al., 2022; Jie et al., 2023; Liang et al., 2024a; Liang et al., 2024b; Yang et al., 2024). Specifically, probiotics regulate GMMs mainly by reducing TMAO levels, increasing ALC, elevating SCFAs, and promoting bile acid synthesis and metabolism (Liang et al., 2020; Tang J. et al., 2021; Kashyap et al., 2022; Liang et al., 2024a).

According to the chemical structure of prebiotics, the prebiotics included in this study can be categorized into bioactive polysaccharides, dietary fiber, and saccharide structures (Xiong et al., 2019; Zhen et al., 2022; Hutchison et al., 2023; Li et al., 2024a; Li et al., 2024b; Li et al., 2024f; Qi et al., 2024; Xing et al., 2024; Zhu et al., 2024). Research on the therapeutic effects of prebiotics on AS suggests that these compounds primarily exert anti-AS effects by modulating the composition of GM, optimizing LM pathways, reducing IR, and enhancing the integrity of IMB (Supplementary Table 5; Xiong et al., 2019; Zhen et al., 2022; Hutchison et al., 2023; Li et al., 2024a; Li et al., 2024b; Li et al., 2024f; Qi et al., 2024; Xing et al., 2024; Zhu et al., 2024).

#### 3.4.2 Dietary intervention

In research on dietary interventions for AS, specific dietary patterns and foods have been found to significantly inhibit the progression of AS (Dong et al., 2019; He et al., 2019; Millar et al., 2019; Moghadasian et al., 2019; Millar et al., 2020; Yue et al., 2020; Ghosh et al., 2021; Guo et al., 2021; Liu F. et al., 2021; Zhao et al., 2021; Gao H. et al., 2022; Huang Y. et al., 2022; Li Y. W. et al., 2022; Li Y. W. et al., 2022; Liu J. et al., 2022; Cheng et al., 2023; Huang T. et al., 2023; Kawamata et al., 2023; Wu et al., 2023; Shen et al., 2024). The mechanisms underlying these effects involve multiple biological processes, including enhancement of GM composition, regulation of microbial metabolites, reduction of IR, improvement of LM, and maintenance of IMB integrity (Dong et al., 2019; He et al., 2019; Millar et al., 2019; Moghadasian et al., 2019; Millar et al., 2020; Yue et al., 2020; Ghosh et al., 2021; Guo et al., 2021; Liu F. et al., 2021; Zhao et al., 2021; Gao H. et al., 2022; Huang Y. et al., 2022; Li Y. W. et al., 2022; Li Y. W. et al., 2022; Liu J. et al., 2022; Cheng et al., 2023; Huang T. et al., 2023; Kawamata et al., 2023; Wu et al., 2023; Shen et al., 2024). Particularly in terms of regulating GMMs, dietary interventions have been demonstrated to: efficiently lower serum levels of TMAO, increase the concentration of SCFAs (He et al., 2019; Moghadasian et al., 2019; Liu F. et al., 2021; Zhao et al., 2021; Li Y. W. et al., 2022; Liu J. et al., 2022). These mechanisms work together to inhibit AS. In contrast, the use of fried oils and ingestion of food-grade titanium dioxide particles (E171) have been found to promote the development of AS (Supplementary Table 5; Kwek et al., 2022; Zhu et al., 2022).

#### 3.4.3 Natural products

The natural compounds included in these studies were classified according to their chemical structures, primarily consisting of carotenoids, alkaloids, flavonoids, phenolic acids and their derivatives, polyphenols, triterpenoids, and other natural compounds (Gautam et al., 2019; Han et al., 2019; Luo et al., 2019; Nie et al., 2019; Wil et al., 2019; Ding et al., 2020; Li et al., 2020; Wang et al., 2020; Wu et al., 2020; Gu et al., 2021; Li X. X. et al., 2021; Liu F. et al., 2021; Lv et al., 2021; Shi et al., 2021; Tsutsumi et al., 2021; Wang F. et al., 2021; Wang J. et al., 2021; Xie et al., 2021; Yang S. et al., 2021; Zhang et al., 2021b; Clark et al., 2022; Dai et al., 2022; Gao M. et al., 2022; Han et al., 2022; Lin et al., 2022; Liu D. et al., 2022; Liu Y. J. et al., 2022; Ma et al., 2022; Panyod et al., 2022; Qiao et al., 2022; Shan et al., 2022; Shi H. et al., 2022; Wang A. et al., 2022; Wu et al., 2022; Xie et al., 2022; Xu et al., 2022; Zhang et al., 2022; Du et al., 2023; He et al., 2023; Jia A. et al., 2023; Kasahara et al., 2023; Liu R. et al., 2023; Liu Y. et al., 2023; Panyod et al., 2023; Tu et al., 2023; Wang G. et al., 2023; Ding et al., 2024; He et al., 2024; Jiang et al., 2024; Jin et al., 2024; Li et al., 2024g; Liang et al., 2024c; Ma et al., 2024; Sun et al., 2024; Wang Y. et al., 2024). The mechanisms by which the natural compounds included in the studies inhibit the development of AS mainly involve improving the composition of GM, regulating microbial metabolites, reducing IR, improving LM, and protecting the integrity of the IMB (Supplementary Table 5; Gautam et al., 2019; Han et al., 2019; Luo et al., 2019; Nie et al., 2019; Wil et al., 2019; Ding et al., 2020; Li et al., 2020; Wang et al., 2020; Wu et al., 2020; Gu et al., 2021; Li X. X. et al., 2021; Liu F. et al., 2021; Lv et al., 2021; Shi et al., 2021; Tsutsumi et al., 2021; Wang F. et al., 2021; Wang J. et al., 2021; Xie et al., 2021; Yang S. et al., 2021; Zhang et al., 2021b; Clark et al., 2022; Dai et al., 2022; Gao M. et al., 2022; Han et al., 2022; Lin et al., 2022; Liu D. et al., 2022; Liu Y. J. et al., 2022; Ma et al., 2022; Panyod et al., 2022; Qiao et al., 2022; Shan et al., 2022; Shi H. et al., 2022; Wang Y. et al., 2022; Wu et al., 2022; Xie et al., 2022; Xu et al., 2022; Zhang et al., 2022; Du et al., 2023; He et al., 2023; Jia A. et al., 2023; Kasahara et al., 2023; Liu R. et al., 2023; Liu Y. et al., 2023; Panyod et al., 2023; Tu et al., 2023; Wang G. et al., 2023; Ding et al., 2024; He et al., 2024; Jiang et al., 2024; Jin et al., 2024; Li et al., 2024g; Liang et al., 2024c; Ma et al., 2024; Sun et al., 2024; Wang Y. et al., 2024).

#### 3.4.4 Traditional Chinese medicine

In the literature reviewed for this study, the compounds were systematically classified according to the principles of TCM dialectics and their primary pharmacological effects (Ji et al., 2020; Zhu et al., 2020; Yang R. et al., 2021; Zhang et al., 2021a; Dongliang et al., 2022; Qi et al., 2022; Wang A. et al., 2022; Yang Q. et al., 2022; Fu et al., 2023; Liao et al., 2023; Wan et al., 2024; Yu et al., 2024; Yue et al., 2024). The identified categories included blood-activating and stasis-resolving, qi-invigorating and surface-strengthening, heat-clearing and toxicity-relieving, tranquilizing and mind-stabilizing, and diuretic and dampness-removing. The mechanisms of TCM treating AS mainly include beneficial regulation of the GM, regulation of microbial metabolites, enhancement of the IMB, improvement of LM and mitigation of IR (Supplementary Table 5; Ji et al., 2020; Zhu et al., 2020; Yang R. et al., 2021; Zhang et al., 2021a; Dongliang et al., 2022; Qi et al., 2022; Wang A. et al., 2022; Yang Q. et al., 2022; Fu et al., 2023; Liao et al., 2023; Wan et al., 2024; Yu et al., 2024; Yue et al., 2024).

#### 3.4.5 Western medicine

The drugs included in the study were classified according to their primary use as antibiotics, hypoglycemics, anticoagulants, liver-protecting agents, antiparasitics, anti-alcohol dependent drugs, antimalarials, and treatments for Parkinson’s disease (Supplementary Table 5; Shi et al., 2019; Kappel et al., 2020; Garshick et al., 2021; Yan et al., 2021; Bai et al., 2022; Li X. L. et al., 2022; Yang X. Y. et al., 2022; Bai et al., 2023; Hao et al., 2023; Tian et al., 2023; Traughber et al., 2023; Miao et al., 2024; Traughber et al., 2024). In the study investigating the mechanisms of Western medicine in the treatment of AS, researchers identified several potential pharmacological mechanisms (Shi et al., 2019; Yan et al., 2021; Bai et al., 2022; Li X. L. et al., 2022; Yang X. Y. et al., 2022; Bai et al., 2023; Hao et al., 2023; Tian et al., 2023; Traughber et al., 2023; Miao et al., 2024; Traughber et al., 2024). These include enhancing the composition of GM, regulating microbial metabolites, improving LM, inhibiting IR, and maintaining the stability of the IMB (Shi et al., 2019; Yan et al., 2021; Bai et al., 2022; Li X. L. et al., 2022; Yang X. Y. et al., 2022; Bai et al., 2023; Hao et al., 2023; Tian et al., 2023; Traughber et al., 2023; Miao et al., 2024; Traughber et al., 2024). Specifically, Guolin et al. found in a hamster experiment that intermittent antibiotic application significantly inhibited AS progression (Miao et al., 2024). In contrast, two independent studies have shown that the use of antibiotics may cause dysbiosis of the GM, enhancing IR, and worsening AS (Kappel et al., 2020; Garshick et al., 2021).

#### 3.4.6 Exercise and others

Endurance exercise, evening exercise, directed chemical manipulation, fecal microbiota transplantation (FMT), and human umbilical cord mesenchymal stem cell (UCSCs) transplantation can inhibit the formation of atherosclerotic plaques (Supplementary Table 5; Chen et al., 2020; Li Y. H. et al., 2021; Huang W. C. et al., 2022; Kim et al., 2022; Schönke et al., 2023). Exposure to arsenic, chronic intermittent hypoxia, propamocarb, acrolein, IL-10 deficiency, and intermittent hypoxia/hypercapnia can promote the development of AS (Wu F. et al., 2019; Wu F. et al., 2019; Hu et al., 2021; Jin et al., 2021; Xue et al., 2021; Shi H. et al., 2022). Diesel exhaust particles (DEP) do not appear to promote AS development (van den Brule et al., 2021).

## 4 Discussion

### 4.1 The impact of GM on the development of AS

Several studies have demonstrated that the composition of the GM in patients with AS differs significantly from that in healthy individuals (Tuomisto et al., 2019; Kwun et al., 2020; Toya et al., 2020; Chen et al., 2021; Ji et al., 2021; Szabo et al., 2021; Choroszy et al., 2022; Szabo et al., 2022; Chen L. et al., 2023; Lv et al., 2023; Samuthpongtorn et al., 2023; Sayols-Baixeras et al., 2023; Wang Z. et al., 2023; An et al., 2024; Li et al., 2024d; Masiá et al., 2024). For example, the abundance of *Fusobacterium nucleatum*, *Metaviridae*, and *Proteobacteria* is greater in patients with AS, whereas the abundance of *Bifidobacterium*, *Faecalibacterium*, and *Bacteroidetes* is decreased (Tuomisto et al., 2019; Kwun et al., 2020; Toya et al., 2020; Chen et al., 2021; Ji et al., 2021; Szabo et al., 2021; Choroszy et al., 2022; Szabo et al., 2022; Chen L. et al., 2023; Lv et al., 2023; Samuthpongtorn et al., 2023; Sayols-Baixeras et al., 2023; Wang Z. et al., 2023; An et al., 2024; Li et al., 2024d; Masiá et al., 2024). In case-control studies, researchers found that changes in the abundance of fifteen gut fungi, including *Mucoromycota*, *Mortierellomycota*, *Mucoromycetes*, *Mortierellomycetes*, and *Tremellomycetes*, are closely associated with the development of CAD (An et al., 2024). This study further suggested that interactions between fungi may affect the onset and progression of CAD (An et al., 2024). In the future, gut fungi are expected to become diagnostic tools for identifying patients with CAD and for assessing disease severity (An et al., 2024). Nakajima et al. found that microbiota such as *Paraprevotella*, *Succinatimonas*, and *Bacillus* are associated with acute coronary syndrome (ACS), while *Lachnospira* is related to stableina pectoris (SAP) (Nakajima et al., 2022). A study tracking longitudinal alterations in the GM of patients with ACS during the first postoperative year revealed that the longitudinal evolution of GM was directly associated with atherosclerotic plaque progression (Fernández-Avila et al., 2024). The cohort utilizing repeated measurement techniques revealed that alterations in the GM (e.g., reduced microbial diversity) occurred synchronously with elevated inflammatory markers (e.g., CD4 + T lymphocytes) and were associated with plaque instability (Fernández-Avila et al., 2024). Wang et al. discovered that *Fusobacterium nucleatum* was positively correlated with carotid plaque in HIV-infected or at-risk women, while five GM, including *Roseburia hominis* and *Roseburia inulinivorans*, were negatively correlated with carotid plaque (Wang Z. et al., 2023). Researchers have also found that the microbial metabolite imidazole propionate (ImP) is significantly positively correlated with carotid plaque and positively associated with various pro-inflammatory markers (Wang Z. et al., 2023). In a large population-based cohort study, an increased abundance of *Streptococcus anginosus* and *S. oralis subsp. oralis* in the GM was significantly associated with coronary AS and systemic inflammation (Sayols-Baixeras et al., 2023). A study has found that in patients with gastrointestinal symptoms, small intestinal bacterial overgrowth (SIBO) is associated with a higher prevalence of subclinical atherosclerotic plaques in the carotid artery, abdominal aorta, and lower extremity arteries (Dong et al., 2022). This association is independent of other traditional cardiovascular risk factors (Dong et al., 2022). A study has for the first time discovered that patients with PAD exhibit high levels of lipopolysaccharides (LPS), suggesting that modulating the GM and intestinal permeability may serve as novel targets for preventing atherosclerotic complications (Loffredo et al., 2020). These findings offer crucial clues for further exploring the role of GM in cardiovascular diseases (CD) and may offer new strategies for the evaluation and therapy of these diseases (Table 3).

**TABLE 3:** Characteristics of the research that were considered in the analysis of the associated GM in human with AS.

References	Author	Time	Study population and disease status	Main gut microbiota associated with atherosclerosis diseases
Tuomisto et al. (2019)	Sari Tuomisto et al	2019	Male autopsy cases (*n* = 67, age range 44–95).	**Increase:***Clostridium leptum*. **Decrease:***Bifidobacterium*. The increase of *Enterobacteriaceae* is associated with larger fibrotic areas of coronary artery plaques. The increase in *Clostridium leptum group* is associated with a larger calcified area. The increase of *Streptococcus spp.* is related to the area of coronary artery calcification.
Chen L. et al. (2023)	Liuying Chen et al	2023	31 patients with CAD and 21 healthy controls.	**Decrease:** *Blautia, Fusicatenibacter*, *Monoglobus*, and *Eubacterium*. **Increase:** *Sutterella*, *Lachnospiraceae_NK4A136_group, UCG-002*, *UCG-005*, *[Eubacterium]_hallii_grou*, *Collinsella*, *Colidextribacter*, *NK4A214_group*, *Negativibacillus*, *Faecalitalea*, *Family_XIII_AD3011_group*, *Peptoniphilus*, *Fructilactobacillus*, and *Solobacterium*.
An et al. (2024)	Kun An et al	2024	31 healthy volunteers and 101 hospitalized patients with CAD. The CAD patients were further divided into three subgroups: (1) stable CAD (SCAD, *N* = 38), (2) unstable angina (UA, *N* = 41), and (3) acute myocardial infarction (AMI, N = 22).	**Negative correlation:** *Mucoromycota*, *Mortierellomycota*, *Mucoromycetes*, *Mortierellomycetes*, *Tremellomycetes*, *Mucorales*, *Mortierellales, Filobasidiales*, *Mortierellaceae*, *Nectriaceae*, *Fusarium*, *Issatchenkia*, *Issatchenkia_orientalis*.
Szabo et al. (2021)	Helga Szabo et al	2021	108 asymptomatic MZ Hungarian twins (54 pairs, mean age 52.4 ± 14.1 years, 58% female).	**Increase:***Firmicutes*. **Decrease:***Bacteroidetes*, *Prevotellaceae*.
Lv et al. (2023)	Hang Lv et al	2023	23 SCAS patients (2 females, 21 males), along with 27 healthy individuals (12 females, 15 males).	**Increase:** *Verrucomicrobia*, *Actinobacteria*, *Collinsella, Akkermansia*, *Ruminococcaceae_UCG_014*, *Parabacteroides*, *Phascolarctobacterium*, *Alistipes*, *Ruminococcus_torques_group*, *Odoribacter*, *Lactobacillus*, *Enterococcus*, *Barnesiella.* **Decrease:** *Lachnospira*, *Ochrobactrum*, *Lachnoclostridium*, *Tyzzerella_3*, *Megasphaera*, *Lachnospiraceae_ NK4A136_group*, *Dorea*, *Prevotellaceae_NK3B31_group*, *Sarcina, Paraprevotella*, *Lachnospiraceae_UCG_004*.
Choroszy et al. (2022)	Marcin Choroszy et al	2022	15 CAD patients and 15 healthy individuals.	**Decrease:** *Bacteroidetes*, *Rickenellaceae*, *Tannerelaceae, Prevotellaceae*, *Alistipes*. **Increase:** *Firmicutes*, *Proteobacteria*, and *Actinobacteria*, *Coriobacteriales*, *Ruminococcaceae*.
Kwun et al. (2020)	Ju-Seung Kwun et al	2020	22 STEMI patients and 20 age-matched and sex-matched healthy controls.	**Increase:** *Proteobacteria*, *Enterobacteriaceae*, *Escherichia*, *Parabacteroides*, *Christensenella*. **Decrease**:*Firmicutes*, *Lactobacillales*, *Lactobacillus*.
Toya et al. (2020)	Takumi Toya et al	2020	53 advanced CAD patients and 53 age-, sex-, race-, and BMI-matched controls.	**Increase:***Ruminococcus Gnavus*. **Decrease:***Lachnospiraceae Anaerosporobacter*, *Lachnospiraceae K4B4*, *Ruminococcus Gauvreauii*.
Masiá et al. (2024)	Mar Masiá et al	2024	Participants included were adults (> 18 years old) with HIV infection receiving antiretroviral regimens based on non-nucleoside reverse transcriptase inhibitors (NNRTI) or integrase strand transfer inhibitors (INSTI), with undetectable viral load during at least the last 6 months (HIV-1 RNA levels < 50 copies/mL).	**Increase:***Agathobacter*, *Ruminococcus_2*, *Bilophila*, *Bifidobacterium*. **Decrease:***Faecalibacterium*.
Samuthpongtorn et al. (2023)	Chatpol Samuthpongtorn et al	2023	14 patients with stroke and 15 healthy controls.	**Positive correlation:** *Ruminococcus*, *Streptococcus*, *Actinomyces*, *Dorea.* **Negative correlation:** *Bifidobacterium*, *Faecalibacterium*.
Ji et al. (2021)	Lei Ji et al	2021	32 CAS patients and 32 healthy controls.	**Increase:** *Acidaminococcus*, *Christensenella* and *Lactobacillus*.
Chen et al. (2021)	Jingfeng Chen et al	2021	31 patients diagnosed with CAS (12 women and 19 men; mean age, 51.32 ± 6.73 years) and 51 sex- and age-matched healthy controls (25 women and 26 men; mean age, 48.49 ± 6.17 years).	**Increase:** *Abiotrophia defectiva*, *Acidaminococcus intestini*, *Gemella haemolysans*, *Lactobacillus mucosae*, *Leuconostoc lactis*, *Megasphaera elsdenii*, *Ruminococcus* sp. *JC304*, *Streptococcus anginosus*, *Turicibacter sanguinis*, and *Turicibacter unclassified,Escherichia coli*, *Halomonas unclassified*, *Klebsiella pneumoniae* and *Pantoea unclassified*. **Decrease:** *Bacteroides* sp. *3_1_19*, *P. unclassified* and *Prevotella copri*.
Wang Z. et al. (2023)	Zheng Wang et al	2023	493 wihs women. the wihs was a multicenter cohort study of women with or at risk for hiv infection, now continuing as part of the multicenter aids cohort study (macs)-wihs combined cohort study.	**Increase:** *Fusobacterium nucleatum*. **Decrease:** *Roseburia hominis*, *Roseburia inulinivorans*, *Odoribacter splanchnicus*, *Clostridium saccharolyticum* and *Johnsonella ignava.*
Szabo et al. (2022)	Helga Szabo et al	2022	22 patients with osa, 16 with and 6 without carotid atherosclerosis.	Decreased diversity of gut microbiota is associated with increased IMT. **Increase:** *Escherichia-Shigella*, *Prevotella*, *Ruminococcaceae*. **Decrease:***Peptostreptococcaceae*.
Sayols-Baixeras et al. (2023)	Sergi Sayols-Baixeras et al	2023	8973 participants aged 50–65 without overt atherosclerotic disease from the population-based swedish Cardiopulmonary bioimage study (SCAPIS).	The shannon diversity index of gut microbiota is negatively correlated with CACS. **Increase:** *Streptococcus* and *Vibrio genera*.
Li et al. (2024d)	Youshan Li et al	2024	214 ACVD patients and 171 healthy volunteers.	**Increas:** *Metaviridae*, *Autographiviridae*, *Siphoviridae*. **Decrease:** *Quimbyviridae*, *unclassified viruses*.
Nakajima et al. (2022)	Akihiro Nakajima et al	2022	Patients with stable angina pectoris (SAP) or acute coronary syndromes (ACS) who underwent cardiac catheterization were enrolled.	*Christensenellaceae*, *Synergestapeae*, *Marinifilaceae*, *Desulfovibrio*, *Pseudomonadaceae* are associated with acute coronary syndrome (ACS),*Christensenellaceae R7 group*, *Cloacibacillus*, *ParaPrevotella*, *Butyricimonas*, *Bilophila* are associated with ACS. *Lachnospira*, *Fusicatenibacter*, and stable angina spectators (SAP) are related.
Dong et al. (2022)	Changhao Dong et al	2022	411 patients were included in this study (mean age, 59.2 years; range, 28–85 years), of whom 241 (58.6%) were diagnosed as SIBO positive and 170 (41.4%) as SIBO negative.	The overgrowth of small intestinal bacteria is positively correlated with the incidence of abdominal aortic plaque, carotid artery plaque, and lower limb arterial plaque.
Loffredo et al. (2020)	Lorenzo Loffredo et al	2020	40 consecutive PAD patients.	Not mentioned changes in the composition of gut microbiota.

CAD, Coronary artery disease; CAS, Carotid atherosclerosis; SCAD, Stable Coronary Artery Disease; UA, Unstable angina; AMI, Acute myocardial infarction; NNRTI, Non-nucleoside reverse transcriptase inhibitors; INSTI, Integrase strand transfer inhibitors; SAP, Stable angina pectoris; ACS, Acute coronary syndromes; ACVD, Acute cardiovascular disease; PAD, Peripheral Artery Disease; SCAS, Symptomatic carotid atherosclerosis; STEMI, ST-segment elevation myocardial infarction; CACS, Coronary artery calcium score; SCAPIS, Swedish Cardiopulmonary BioImage Study; OSA, Obstructive Sleep Apnea; The WIHS was a multicenter cohort study of women with or at risk for HIV infection, now continuing as part of the Multicenter AIDS Cohort Study (MACS)-WIHS Combined Cohort Study.

Disruption of the GM may exacerbate gut dysbiosis (GD) through interactions among microbial communities, thereby promoting the development of AS (Brandsma et al., 2019; Kiouptsi et al., 2019; Carnevale et al., 2020; Liu Q. et al., 2020; Sun et al., 2021; Shi G. et al., 2022; Zhang et al., 2023; Wang X. et al., 2024). Gut microbiota dysbiosis(GMD)can lead to higher levels of LPS, augmented production of harmful metabolites, reduced levels of beneficial metabolites, enhanced IR, aggravated LMD, disruption of the gut mucosal barrier, and increased gut permeability (Brandsma et al., 2019; Kiouptsi et al., 2019; Carnevale et al., 2020; Liu Q. et al., 2020; Krupa et al., 2021; Sun et al., 2021; Shi G. et al., 2022; Zhang et al., 2023; Wang X. et al., 2024). Studies have found that an increased abundance of *Candida albicans (C. albicans)* can enhance the production of its metabolite formyl-methionine (f-Met), which in turn activates HIF-2α signaling in the gut, leading to an increase in the pro-inflammatory factor IL-1b and exacerbating the IR (Wang X. et al., 2024). Moreover, *C. albicans* can elevate cholesterol levels in the serum and liver, contributing to AS (Wang X. et al., 2024). In a study by Brandsma et al., transplantation of fecal microbiota from *Caspase1-/-* mice with a pro-inflammatory microbial community into antibiotic-treated Ldlr−/− mice resulted in a significant reduction in the abundance of SCFA-producing bacteria, such as *Akkermansia* and *Christensenellaceae*, in the recipient mouse gut, leading to decreased SCFAs levels (Brandsma et al., 2019). This promoted a significant increase in the number of Ly6Clo and Ly6Chi monocytes and neutrophils in the blood as well as elevated pro-inflammatory cytokine levels, such as IL-1β and IL-2, in the plasma, exacerbating inflammation (Brandsma et al., 2019). *Desulfovibrio desulfuricans (D. desulfuricans)* can decrease the number of bacteria, including *Akkermansia muciniphila* and *prausnitzii*, increase LPS levels, enhance the expression of TLR4 and P-p65 and activate the TLR4/NF-κB signaling pathway (Zhang et al., 2023). This results in increased serum levels of the pro-inflammatory cytokines TNF-α, IL-1b, and IL-6, exacerbating local and SIR (Zhang et al., 2023). *D. desulfuricans* can disrupt the gut mucosal barrier, increase gut permeability, and aggravate LPS-induced IR (Zhang et al., 2023). Roberto Carnevale and colleagues found that LPS enhances thrombosis at the site of coronary unstable plaque rupture through TLR4-mediated leukocyte-platelet interaction (Carnevale et al., 2020). Kiouptsi showed that gut commensal microbiota can enhance low-grade inflammation in the vascular wall and weaken type I and III collagen-dependent platelet activation, leading to plaque rupture (Kiouptsi et al., 2019). In summary, GMD promotes the occurrence and progression of AS through various mechanisms, including affecting lipid levels, increasing IR, altering the balance between harmful and beneficial bacteria, and the direct action of pathogenic bacteria. Therapeutic strategies targeting GMD hold promise as new targets for preventing and treating CD.

The presence of bacteria within atherosclerotic plaques in patients with AS has been confirmed by numerous studies (Koren et al., 2011; Fernandes et al., 2014). Among these bacteria, some colonize both oral and intestinal ecosystems (Koren et al., 2011). Research indicates that microbes can be transmitted from the oral cavity to the gut, suggesting that oral and gut microbiomes can influence each other (Sayols-Baixeras et al., 2023). Several studies have demonstrated that chronic periodontitis, induced by oral bacteria, can exacerbate AS (Xiao et al., 2021; Gan et al., 2022; Gan et al., 2023; Park et al., 2023; Gan et al., 2024a). The study has shown that chronic periapical periodontitis (CAP) not only accelerates the progression of AS but also significantly alters the composition and diversity of the GM, revealing a close relationship between the two (Gan et al., 2022). CAP leads to GD through the proliferation of harmful bacteria, such as *Odoribacter* and *Erysipelotrichaceae*, and decreasing the number of beneficial bacteria, such as *Faecalibacterium* and *Lachnospiraceae* (Gan et al., 2024a). Dysbiosis not only inhibits the synthesis of primary BAs but also promotes the elevation of metabolite levels, such as TCDCA, TCA, and TDCA, thereby exacerbating LM disorders and promoting the development of AS (Gan et al., 2024a). CAP also enhances the number of bacteria such as *Lachnospiraceae* and *Porphyromonadaceae*, which are positively correlated with TMAO, leading to increased TMAO concentrations and IR (Gan et al., 2023). Periodontitis alters the composition of the GM, increases the levels of endotoxins, and subsequently upregulates the expression of FMO3 in the liver, thereby increasing TMAO production and exacerbating inflammation (Xiao et al., 2021). *Porphyromonas gingivalis (PG)* infection can increase the relative abundance of *Actinobacteria* and *Deferribacteres* while reducing the abundance of *Lactobacillus gasseri* and *Mucispirillum schaedleri*, thereby altering the GM composition (Park et al., 2023). Changing in the oral microbiota may promote AS progression by affecting the structure of the GM. This mechanism reveals the complex association between periodontitis and AS and provides a new perspective for preventive strategies. In other words, the intervention of the gut and oral microbiota may be an effective approach to prevent AS (Supplementary Table 6).

### 4.2 The impact of GMMs on AS

#### 4.2.1 TMAO

**FIGURE 4:**
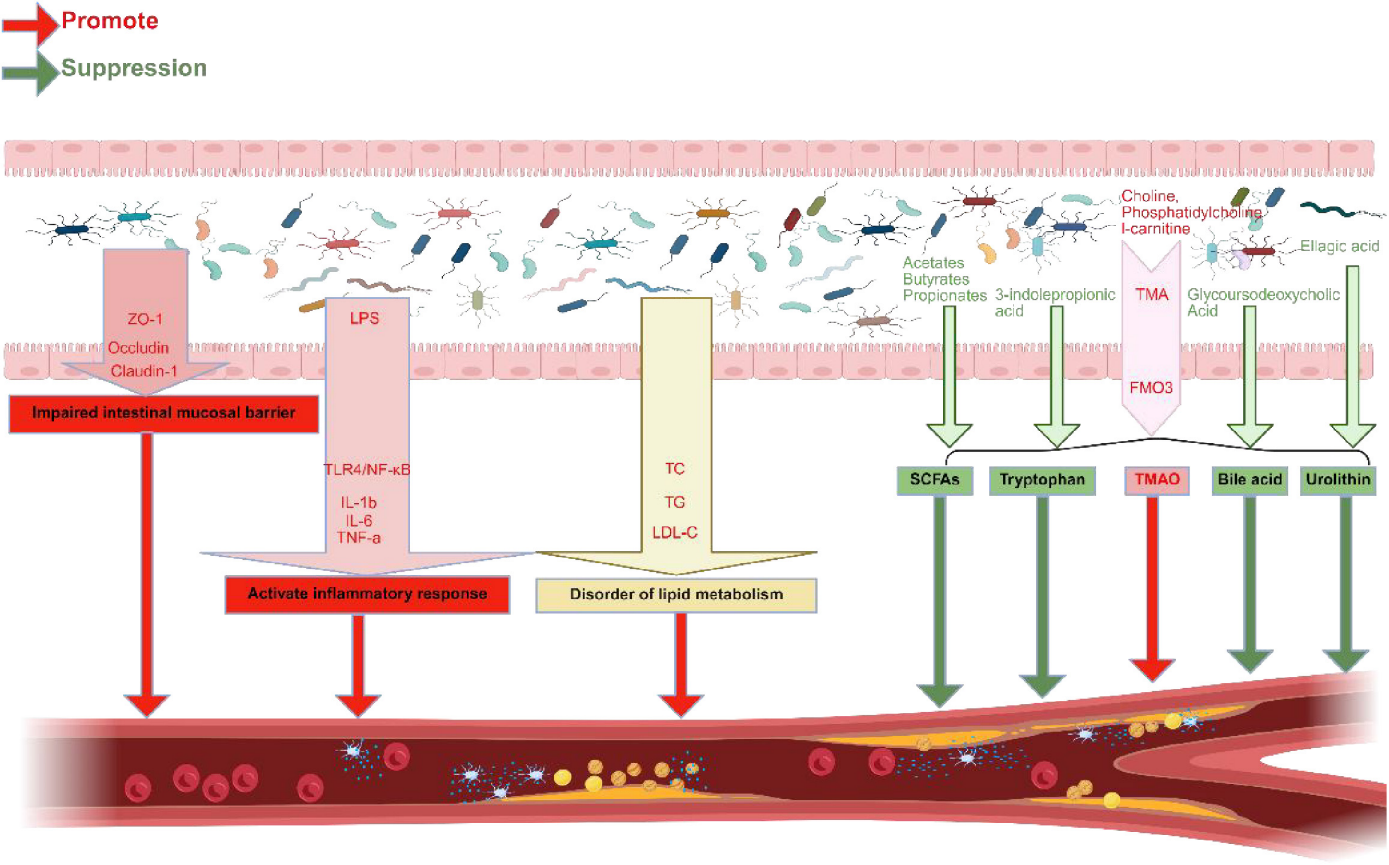
Possible mechanisms by which GM and GMMs influence AS. GMD can downregulate the expression of intestinal tight junction proteins (such as ZO-1, Occludin, and Claudin-1), increase intestinal permeability, and thereby promote the development of AS. GMD can also activate the TLR4/NF-κB signaling pathway through LPS mediation, leading to elevated levels of serum pro-inflammatory cytokines (such as IL-1β, IL-6, and TNF-α), exacerbating inflammatory responses. GMD can cause elevated levels of serum TG, TC, and LDL-C, promote lipid metabolism disorders, and further drive AS progression. The GMMs included in the study were TMAO, SCFAs, tryptophan, bile acids, and urolithin, among others. Dietary precursors (such as choline, phosphatidylcholine, and l-carnitine) are converted into TMA by transgenic TMA lyase, which is then oxidized to TMAO by flavin-containing monooxygenase (FMO, particularly FMO3). TMAO promotes the development of AS. SCFAs, including acetate, butyrate, and propionate, are produced by anaerobic microbiota in the cecum and colon through the fermentation of dietary fiber. SCFAs can inhibit atherosclerosis progression, and tryptophan-derived compounds, such as 3-indolepropionic acid, are metabolites of the intestinal microbiota that can inhibit the progression of AS. Bile acids, such as glycoursodeoxycholic acid (GCA), are metabolites of the intestinal microbiota that can inhibit AS progression. Urolithins are produced by the intestinal microbiota through the metabolism of dietary polyphenols (particularly ellagic acid) and can inhibit AS progression. Created in BioRender: Dezhi (2025) (https://BioRender.com/i40z814).

Trimethylamine (TMA) is a low-boiling nitrogen-containing small-molecule chemical, dietary precursors such as choline, phosphatidylcholine, and l-carnitine, which are prevalent in meats, dairy products, eggs, and fish, are transformed to TMA by genetically modified TMA lyases (Goel et al., 2016; Simó and García-Cañas, 2020). Following intestinal absorption, TMA travels through the portal vein to the liver, where it is converted to TMAO by the flavin-containing monooxygenase (FMO) family, namely FMO3 (Gatarek and Kaluzna-Czaplinska, 2021). Choline conversion to TMA, which elevates TMAO levels, may be facilitated by microorganisms, such as *L. saccharolyticum WM*1 (Cai et al., 2022). TMAO levels are positively correlated with CAD, suggesting that TMAO may be involved in the pathogenesis of AS (Tang W. H. W. et al., 2021). Studies have revealed that TMAO, a key molecule produced by hepatic metabolism, may act through the “liver-heart axis” to further influence the cardiovascular system (Zhou et al., 2025). The liver-heart axis refers to the intricate relationship between the liver and heart, highlighting their bidirectional influence on each other’s health (Ambale-Venkatesh and Lima, 2019). The mechanisms by which TMAO affects AS may involve several factors. TMAO can promote IR by upregulating the secretion of pro-inflammatory cytokines (such as IL-1β, IL-6, and TNF-α) and increasing the protein expression of ICAM-1 and p-NF-κB p65/NF-κBp65, thereby exacerbating inflammation (Chen C. Y. et al., 2023). TMAO may promote the expression of NLRP3 inflammasomes and ASC, and activate NF-κB p65, thus affecting the progression of AS (Chen C. Y. et al., 2023). TMAO also inhibits SIRT1, promotes ROS generation, and increases the inflammatory cytokine levels, exacerbating the IR (Zhou et al., 2022). TMAO may influence the composition and function of GM, therefore influencing the path of AS (Zhou et al., 2022). The intake of choline and TMAO increases the species diversity of the GM, particularly the proportional abundance of *Bacteroidetes* and *Firmicutes*, which could result in increased inflammation and compromised intestinal barrier function (Zhou et al., 2022). Notably, the predictive effect of TMAO levels may differ between sexes, with a better prediction in males than in females, suggesting that the ways in which TMAO influences AS may be significantly influenced by sex characteristics (Guo et al., 2020). However, some studies have shown no correlation between TMAO concentrations and AS progression (Aldana-Hernández et al., 2020; Collins et al., 2022). For animal experiments, some important factors, including animal background, source or procurement, housing conditions (single- or multi-housed, caging types, etc.) and facility containment levels also play a role in influencing the gut microbiome and atherosclerosis outcome (Aldana-Hernández et al., 2020; Collins et al., 2022). These conflicting results partially stem from the relative paucity and heterogeneity of human data, underscoring the need to validate the findings from animal models in broader human populations. This may suggest that TMAO’s role of TMAO in the pathogenesis of AS is not singular. In female cohorts, an increased intake of dietary fiber was positively correlated with higher TMAO concentrations, whereas no such correlation was observed in the male cohort (Almer et al., 2024). This finding suggests the presence of sex-specific metabolic pathways (Almer et al., 2024). The study posits that these sex differences may be attributed to variations in gut microbial composition or enzyme activity, although further research is necessary to elucidate the precise mechanisms involved (Almer et al., 2024). In summary, TMAO contributes to the onset and progression of AS through multiple pathways, including promoting IR and affecting GM (Figure 4; Supplementary Table 7). Further research into the specific mechanisms of TMAO in AS will assist develop fresh approaches to AS treatment and prevention.

#### 4.2.2 SCFAs

SCFAs, which include acetates, butyrates, and propionates, are mostly created when anaerobic bacteria degrade undigested food fiber in the large intestine and cecum (Macfarlane and Macfarlane, 2003). SCFAs are key regulatory factors in LM and the inhibition of IR (Bultman, 2018). They are produced by different GM metabolisms; for example, *Bacteroidetes* produce acetic and propionic acids, whereas Firmicutes produce butyric acid (Jia A. et al., 2023). Studies have shown that SCFAs play a significant role in regulating LM, improving IR, and maintaining the intestinal barrier function. In a cross-sectional study, fecal butyrate levels in patients with AS were significantly higher than those in healthy individuals (Sto et al., 2022). However, no substantial correlation was observed between elevated butyrate levels and markers of inflammasome activation, suggesting that butyrate may influence AS at different stages or through distinct mechanisms (Sto et al., 2022). The positive correlation between butyrate levels and butyrate-producing genera, such as *Roseburia* and *Faecalibacterium*, demonstrated the importance of GM in butyrate production (Sto et al., 2022). Importantly, butyrate can promote an increase in IL-1β levels by upregulating the expression of PPARδ and miR-181b while lowering the production of ROS in endothelial cells, consequently preventing the progression of AS (Tian et al., 2021). The increase in butyrate was significantly positively correlated with an increase in bacteria with anti-inflammatory properties, such as *Blautia producta*. Butyrate can downregulate genes related to LPS biosynthesis, further indicating its role in regulating the intestinal immune responses (Liu C. et al., 2022). Butyrate can reduce atherosclerotic inflammation in ApoE−/− mice by preventing M1 macrophage polarization, enhancing the activation of M2 macrophages, and regulating the GPR43/HDAC-miRNAs axis (Ma et al., 2023). Ma et al. also found that butyrate could lower the serum cholesterol levels in ApoE−/− mice (Ma et al., 2023). However, the studies have found no significant correlation between butyrate and plasma lipid levels or AS development (Sto et al., 2022). Propionate can improve LM and exert anti-AS effects by increasing the production of regulatory IL-10 and regulatory T cells in the small intestine, and by regulating the expression of genes that are associated with cholesterol metabolism, such as Srebp2 and Cyp7a1 (Haghikia et al., 2022). In summary, SCFAs are involved in the regulation of AS through multiple pathways, including regulation of the GM, improvement of intestinal barrier function, immune responses, and LM (Figure 4; Supplementary Table 7). The potential molecular mechanisms of SCFAs warrant further in-depth investigation.

The production of 3-indolepropionic acid (IPA), IPA is a tryptophan derivative, is entirely dependent on the presence of the GM (Wikoff et al., 2009). Studies have shown that the level of IPA, a metabolic product of GM, is significantly reduced in patients with CAD and is inversely correlated with the risk of atherosclerotic cardiovascular disease (ASCVD) (Xue et al., 2022). These findings imply that IPA plays a pivotal role in the pathogenesis of AS. In another cross-sectional study, it was observed that, in women with or without HIV infection, the plasma levels of IPA and its associated gut bacteria were inversely correlated with carotid artery plaques (Luo K. et al., 2024). Luo et al. believe that IPA and the GM that produce IPA may have a potential protective effect against AS (Luo K. et al., 2024). Xue et al. used integrated metagenomic and metabolomic approaches to discover that the GMD in CAD patients resulted in a significant reduction in the production of IPA (Xue et al., 2022). Furthermore, through animal and cellular experiments, Xue et al. found that IPA could regulate the miR-142-5p/ABCA1 signaling pathway, promote cholesterol reverse transport in macrophages, and effectively inhibit the formation of plaques caused by AS (Xue et al., 2022). In summary, the tryptophan metabolite IPA plays a crucial role in the pathogenesis of AS. Regulating the composition of the GM and production of IPA may be a new strategy for the prevention and management of AS (Figure 4; Supplementary Table 7). Future research should explore the association between the GM and IPA.

Bile acid exerts anti-AS effects by regulating the activation of the immune system and IR as well as by improving lipid profiles. Glycine lipids, a metabolic product of the GM, inhibit immune system activation by downregulating the expression of Major Histocompatibility Complex (MHC) II-related genes, including H2aa, H2ab1, H2eb1, H2dma, H2dmb1, Cd74, and Irf8 (Millar et al., 2022). Glycine lipids also significantly reduced the manifestation of inflammatory marker genes such as IL-1β and inhibited IFNγ-induced manifestation of H2aa, H2eb1, and IL-1β, further alleviating the IR (Millar et al., 2022). Glycoursodeoxycholic Acid (GCA) inhibits macrophage recruitment and downregulates Monocyte Chemoattractant Protein-1 (MCP-1) and IL-1β mRNA expression, thereby reducing inflammatory cell infiltration (Huang et al., 2021). Glycine lipids are capable of lowering serum levels of total cholesterol (TC), non-high-density lipoprotein cholesterol (non-HDL-C), and free fatty acids while also reducing cholesterol accumulation in the liver, all of which contribute to slowing the progression of AS (Millar et al., 2022). GCA is able to reduce the levels of total triglycerides (TGs), TC, and low-density lipoprotein cholesterol (LDL-C) in the liver; decrease serum TC and LDL-C; inhibit the expression of Acyl-CoA Cholesterol Acyltransferase 2 (ACAT2); and promote the excretion of fecal cholesterol, all of which help to improve lipid profiles and alleviate the pathological changes of AS (Huang et al., 2021). GCA also modulates the composition of the GM, particularly by increasing the abundance of *Parabacteroides* and *Alloprevotella*, which are negatively correlated with AS progression, while simultaneously reducing the abundance of *Turicibacter* and *Alistipes*, which are positively correlated with AS progression, thus aiding AS suppression (Huang et al., 2021). In summary, glycine lipids and GCA collectively play a role in mitigating the development of AS through multiple mechanisms, including regulation of immune responses, reduction in the infiltration of inflammatory cells, and improvement of lipid levels (Figure 4; Supplementary Table 7). These studies provide novel insights into future preventive and therapeutic strategies, suggesting that gut microbiota-derived bile acids may be potential targets for intervention in AS.

Urolithin is primarily derived from dietary polyphenols, especially ellagic acid, which is widely found in foods, such as berries, nuts, and pomegranates (D’Amico et al., 2021). Within the urolithin family, urolithin A (UroA) and urolithin B (UroB) are the most thoroughly studied subtypes. UroA can mitigate endothelial inflammation by reducing macrophage content in plaques, inhibiting the manifestation of endothelial adhesion molecules, promoting the production of NO, and downregulating the expression of YAP/TAZ proteins and TEAD transcriptional activity (Xu et al., 2024). UroA also improves cholesterol metabolism by regulating transcription and cleavage of the lipidogenic transcription factor SREBP1/2 in the liver (Xu et al., 2024). UroB, on the other hand, not only upregulates the expression of SR-BI and ABCA1 to promote reverse cholesterol transport but also increases cholesterol efflux from cholesterol-rich macrophages to HDL particles, thereby reducing lipid plaque deposition (Zhao et al., 2019). These results demonstrate the significant potential of urolithin compounds in the prevention and treatment of AS and provide a crucial direction for future research (Figure 4; Supplementary Table 7). Based on the mechanism described above, propose novel targets and directions for intervening in the progression of AS (Supplementary material 2).

### 4.3 The mechanism of various intervention factors on AS

#### 4.3.1 Effects of probiotics and prebiotics on AS

In recent years, studies have revealed the significant effects of specific probiotics on the development of AS (Cruz Neto et al., 2024). The specific strains include *Lactobacillus rhamnosus GG*, *Lactiplantibacillus plantarum ATCC 14917*, *Lactobacillus mucosae A1* and *Lactobacillus plantarum ZDY04* (Cruz Neto et al., 2024). *Bifidobacterium*, a common probiotic, has notable functions in combating AS. Research indicates that *Bifidobacterium* can optimize the structure of the GM by raising the relative abundance of *Desulfobacterota*, *Actinobacteria*, and *Verrucomicrobiota*, while decreasing the relative abundance of *Firmicutes* and *Bacteroidetes*, thereby lowering the *Firmicutes/Bacteroidetes (F/B*) ratio (Liang et al., 2024b). *Bifidobacterium* can regulate the metabolic products of the GM, such as decreasing serumTMA and TMAO levels and enhancing the concentration of anti-inflammatory lipids such as alkyl lysophosphatidylcholine (ALC), producing antioxidant and anti-inflammatory properties (Liang et al., 2020; Liang et al., 2024a). Further studies have found that when Bifidobacterium is used in conjunction with krill oil, it not only reduces serum endotoxin levels but also more effectively alleviates IR and improves LM (Liang et al., 2021). In addition to *Bifidobacterium*, other probiotics, such as *Faecalibacterium prausnitzii*, *Enterobacter aerogenes ZDY01*, *Lactobacillus plantarum ATCC 14917*, and *Lactobacillus rhamnosus GG*, have also shown potential in combating AS. *Faecalibacterium prausnitzii* reduces serum LPS levels and the expression of inflammation-related factors, enhancing IMB function against AS (Yang et al., 2024). *Enterobacter aerogenes ZDY01* increases the abundance of beneficial bacteria, lowers TMAO levels, reduces macrophage content in plaques, and promotes cholesterol transformation and excretion (Tang J. et al., 2021). *Lactobacillus plantarum ATCC 14917* improves GM composition, downregulates the expression of inflammatory factors, and alleviates IR (Hassan et al., 2020). Through enhancing the abundance of lactic acid bacteria(LAB), decreasing the abundance of *Bacteroidetes*, and increasing the SCFAs content, which has a preventive effect on AS (Kobayashi et al., 2021). Taiyu Zhai et al showed that *Lactobacillus rhamnosus GG* increased the diversity of GM and improved its composition, promoting the biosynthesis and metabolism of unsaturated fatty acids and ketone bodies (Zhai et al., 2022). Jie et al. isolated *Bacteroides cellulosilyticus*, *Faecalibacterium prausnitzii*, and *Roseburia intestinalis* from human feces, which, in mouse models, upregulated the expression of the nuclear bile acid receptor farnesoid X receptor (FXR) and Nr4a1 (Nur77), improving LM (Jie et al., 2023). Furthermore, metagenomic analysis of the gut microbiome of patients identified *Bacteroides xylanisolvens*, *Eubacterium eligens*, and *Roseburia inulinivorans* as potential new probiotics or targets for AS treatment (Kashyap et al., 2022). In summary, probiotics exert a positive influence on AS through multiple pathways. Future research should delve deeper into the specific mechanisms of action of different probiotics to provide a richer scientific basis and clinical application prospects for AS prevention and treatment.

Qi et al. showed that Dendrobium officinale polysaccharide significantly reduced the ratio of *Bacteroidetes* to *Firmicutes (F/B)* in the GM, lowered the serum levels of TC, TG, and LDL-C, and reduced the expression levels of inflammatory cytokines IL-1β, and IL-6 and TNF-α, while increasing the levels of anti-inflammatory factors Arg1, Mrc1, Retnla, and Irf4 (Qi et al., 2024). Manno-oligosaccharides from Cassia Seed Gum can protect the integrity of the IMB by promoting the mRNA expression of tight junction proteins (TJPs), thereby reducing intestinal permeability (Li et al., 2024b). Dietary fiber reduces the F/B ratio in the GM, increases the abundance of butyrate-producing bacteria, and increases the total concentration of SCFAs, thereby alleviating the progression of AS (Hutchison et al., 2023). Another study found that inulin decreased the abundance of *Firmicutes* and increased the abundance of *Bacteroidetes*, significantly reducing the F/B ratio, lowering plasma LPS levels and the content of GMMs such as L-glutamine, improving dyslipidemia, and mitigating IR to treat AS (Li et al., 2024f). Further research has shown that prebiotics with sugar chain structures can mitigate AS. Chitin oligosaccharides can increase SCFAs content, reduce serum LPS, improve blood lipid profiles and liver steatosis, decrease the levels of inflammatory factors such as IL-1β, IL-6, and TNF-α to alleviate inflammation, and promote mRNA expression of TJPs to enhance the integrity of the IMB (Zhen et al., 2022). Zhu et al. suggested that sialic acid and 3’-sialyllactose could potentially inhibit the development of AS by decreasing the prevalence of pathogenic bacteria and increasing the prevalence of beneficial bacteria, thereby affecting multiple microbial metabolic pathways (Zhu et al., 2024). These findings present a scientific foundation for the development of polysaccharide-based dietary that may have potential clinical value in the prevention and treatment of AS (Supplementary Table 8).

#### 4.3.2 The impact of diet on AS

The role of diet and its mechanisms in preventing and treating AS have increasingly become the subject of research (Supplementary Table 8). Two studies found that an increased intake of vegetables, fruits, and dairy products has a negative correlation with the incidence of AS (Baragetti et al., 2021; Zhu et al., 2021). The intake of vegetables and fruits can significantly enhance GM diversity and boost the quantity of beneficial bacteria such as *Leuconostoc*, *Trichococcus*, *Turicibacter*, and *Dorea*, and the alterations in GM are linked to mitigating IR, reducing liver steatosis, and improving dyslipidemia (Guo et al., 2021). This demonstrated how important the GM is to the diet-mediated control of AS. In men, whole-fat milk intake was significantly inversely associated with coronary artery calcification (Ghosh et al., 2021). The polar lipids in milk can alter the GM composition to lower the *F/B* ratio and downregulate the expression of CCL4 mRNA in liver tissue and CCL2 mRNA in the aorta to mitigate IR and improve LM, thereby inhibiting the progression of AS (Millar et al., 2020). Cereals and cereal products, oils, proteins, and their degradation products can exert anti-AS effects by changing the composition of the GM and GMMs, mitigating IR, improving LM, and enhancing the integrity of the IMB (He et al., 2019; Millar et al., 2019; Moghadasian et al., 2019; Yue et al., 2020; Gao H. et al., 2022; Huang Y. et al., 2022; Li Y. W. et al., 2022; Liu J. et al., 2022; Huang T. et al., 2023; Wu et al., 2023). Highland barley, a whole grain, has been found to reduce serum TNF-α levels and suppress NLRP3 expression in the aorta (Wu et al., 2023). Highland barley increases the relative abundance of beneficial bacteria with anti-inflammatory properties, such as *Lachnospiraceae, Lactobacillus*, *Muribaculaceae*, and *Bifidobacterium*, further mitigating IR and inhibiting atherosclerotic plaque formation (Wu et al., 2023). He et al. found that fish oil can modulate the expression of genes associated with LM, thereby reducing plasma TC, TG, and non- HDL-C levels (He et al., 2019). It decreases plasma levels of IL-1β, TNF-α, and MCP-1, which collectively mitigate IR (He et al., 2019). Fish oil increases the abundance of GM that produce SCFAs, promoting SCFAs generation and inhibiting microbial LPS production, thus reducing TMAO-aggravated atherosclerotic plaque formation (He et al., 2019). Huang et al. discovered that long-term supplementation with 0.67 g/kg/day can decrease the expression of ICAM-1 and reduce serum levels of TNF-α and IL-1β, thereby mitigating IR (Huang Y. et al., 2022). Additionally, it reduces plasma TG and LDL-C levels, improving LM (Huang Y. et al., 2022). The supplementation also increases the expression of TJPs, enhancing the integrity of the IMB, and alters GM composition to reduce AS induced by a high-fat diet in ApoE−/− mice (Huang Y. et al., 2022). Other dietary and eating habits, such as red yeast rice, moderate alcohol consumption, ketogenic diets, Cabernet Sauvignon dry red wine, and Ligustrum Robustum, can inhibit the formation of AS by regulating the composition of the GM and GMMs, regulating LM, and mitigating inflammation (Dong et al., 2019; Liu F. et al., 2021; Zhao et al., 2021; Cheng et al., 2023; Shen et al., 2024). Cabernet Sauvignon dry red wine enhances the expression of inflammation-related pathways, concurrently leading to a reduction in serum levels of IL-6, IL-1β and inducible nitric oxide synthase (iNOS) (Cheng et al., 2023). The regulation of the ATP-Binding Cassette Transporter A1 (ABCA1), Peroxisome Proliferator-Activated Receptor gamma (PPARγ), and Liver X Receptor alpha (LXR-α) pathways in the liver appears to be crucial for LM and cholesterol efflux (Cheng et al., 2023). It has been observed to enhance the abundance of beneficial GM, such as *Akkermansia*, *Christensenellaceae_R-7*, and *Eubacterium fissicatena*, which may contribute to inhibiting the progression of AS (Cheng et al., 2023). A 21-week randomized controlled trial investigated the effects of dietary fiber and fermented foods in healthy volunteers, demonstrating that dietary interventions significantly improved GM structure and metabolite composition (van de Put et al., 2024). This study was not specifically designed for AS patients, its results provided support for the theoretical basis that GM modulation may indirectly influence AS pathological pathways by improving GMMs (van de Put et al., 2024).

#### 4.3.3 Effects of various natural compounds on AS

##### 4.3.3.1 Carotenoids

Astaxanthin-rich extract (ASTE) can remold the GM, particularly by increasing the abundance of *Akkermansia*, to regulate the expression of genes related to cholesterol metabolism and upregulate the expression of JAM-A, Occludin, and mucin2 in the colon to enhance the intestinal barrier, thereby exerting an anti-AS effect (Supplementary Table 8; Liu D. et al., 2022). Crocin reduces the ratio of *F/B*, increases the relative abundance of *Verrucomicrobia, Akkermansia*, and *Alloprevotella*, and lowers serum LPS levels to mitigate IR (Han et al., 2022). It upregulates the expression of TJPs ZO-1 and occludin in the intestine to protect the function of the mucosal barrier (Han et al., 2022).

##### 4.3.3.2 Alkaloid

Berberine can alter the composition of the intestinal flora to reduce the level of TMAO and increase the level of SCFAs, thereby reducing the level of pro-inflammatory cytokines such as TNF-α, IL-1 β, IL-6, and increasing the level of anti-inflammatory cytokines such as IL-10, thereby reducing the IR (Supplementary Table 8; Wu et al., 2020). Berberine can reduce the relative abundance of TMA-producing bacterial species, such as *Proteus mirabilis*, *Shigella baumannii*, and *Bacteroides fragilis*, in hamsters and inhibit FMOs in the GM, thereby reducing TMAO production (Ma et al., 2022). Ma et al. found that in patients with AS, berberine can also reduce the relative abundance of TMA-producing bacterial species, such as *Eubacteriumcoprostanoligenes_group*, leading to a decrease in TMAO concentrations in the human body. Moreover, the therapeutic efficacy of berberine is superior to that of statins and aspirin (Ma et al., 2022).

##### 4.3.3.3 Flavonoids

Mangiferin has been shown to ameliorate GM structure by reducing the *F/B* ratio, raising the abundance of beneficial genera, such as *Akkermansia*, *Bifidobacteriaceae* and *Parabacteroides*, and decreasing pathogenic *Helicobacter pylori* (Supplementary Table 8; He et al., 2023). This improvement in microbiota composition enhances the production of SCFAs and reduces plasma LPS levels, thereby improving LM and mitigating inflammation. Tilianin activates SREBP2 to increase the expression of Low-Density Lipoprotein Receptor(LDLR), thereby enhancing LDLR-mediated cholesterol uptake and reducing serum LDL-C levels, which ameliorates dyslipidemia and hepatic steatosis, ultimately inhibiting AS development (Du et al., 2023). The effects of tilianin on dyslipidemia are closely linked to changes in characteristics in the composition of the GM (Du et al., 2023). Li et al. found that puerarin could decrease the quantity of *Prevotella copri* and inhibit its ability to produce TMA, resulting in reduced serum TMAO levels (Li et al., 2024g). The improvement in AS by naringin is related to the regulation of cholesterol biosynthesis into bile acids by altering the expression of CYP7A1 and the FXR/FGF15 pathway, which is induced by changes in the abundance of bacteria, such as *Bacteroides*, *Bifidobacterium*, and *Clostridium* (Wang et al., 2020).

##### 4.3.3.4 Phenolic acids

Ding et al. suggested that protocatechuic acid (PCA) increases the abundance of beneficial bacteria such as *Rikenella* and reduces the abundance of harmful bacteria such as *Helicobacter* to improve the GM and enhance the α-diversity of the GM (Supplementary Table 8; Ding et al., 2024). It also mitigates inflammation and upregulates peroxisome proliferator-activated receptor α (PPARα) expression in the liver to improve LM, thereby alleviating TMAO-aggravated AS (Ding et al., 2024). Yarong et al. were the first to confirm that paeonol (Pae) mitigates endothelial inflammation mediated by the ROS/TXNIP/NLRP3 pathway by reducing the production of the GM metabolite hydroxyisobutyric acid (HIBA) (Liu Y. et al., 2023). According to Yarong et al., HIBA may be a useful biomarker for AS clinical diagnosis (Liu Y. et al., 2023). Pae restores the expression of TJPs and enhances the integrity of the IMB by regulating the composition of GM, particularly by significantly reducing the abundance of gram-negative bacteria, thereby mitigating inflammation (Shi H. et al., 2022). It also reduced the manifestation of α-SMA and PCNA in the aorta of ApoE−/− mice through gut microbial mediation, inhibiting the proliferation of vascular smooth muscle cells (VSMCs), thus preventing the development of AS (Shi H. et al., 2022).

##### 4.3.3.5 Polyphenolic natural compounds

Millet shell polyphenols (MSPs) can increase the abundance of *Ruminococcus* and *Oscillospira*, while decreasing *Allobaculum*, thereby reducing the levels of LPS and subsequently lowering the levels of inflammatory cytokines such as IL-1β and TNF-α (Supplementary Table 8; Liu F. et al., 2021). MSPs upregulate the mRNA expression of TJPs occludin, ZO-1, and claudin1 mRNA, thereby enhancing the IMB (Liu F. et al., 2021). Peanut skin extract (PSE) has also demonstrated similar anti-atherosclerotic effects. PSE changes the composition of the GM, particularly by increasing the abundance of *Roseburia*, *Rothia, Parabacteroides*, and *Akkermansia*, while reducing the abundance of *Bilophila* and *Alistipes* (Xu et al., 2022). This contributes to reduced serum TC and LDL-C levels, increased HDL-C levels, and improved LM disorders in ApoE-/- mice (Xu et al., 2022). Additionally, it boosted the levels of the anti-inflammatory cytokine IL-10 and greatly decreased those of the pro-inflammatory cytokines TNF-α and IL-6, which inhibited the IR (Xu et al., 2022). The discovery that polyphenols modulate the GM to interrupt the TMA-FMO3-TMAO pathway and mitigate AS induced by a high-fat diet highlights the role of GM in the atherosclerotic process (Jiang et al., 2024).

##### 4.3.3.6 Triterpenoid natural compounds

Gypenoside XLIX, Ginkgolide B, Ginsenosides, Ganoderma meroterpene derivative, ginsenoside Rb1, Notoginsenoside R1, Astragaloside IV, Thelenota ananas saponin extracts, and Ginsenoside Rc can inhibit the progression of AS by changing the composition of GM, regulating microbial metabolites, improving dyslipidemia, reducing IR, and increasing the expression levels of TJPs, such as TJP1, ZO-1, and Occludin, to enhance the integrity of the IMB (Supplementary Table 8; Han et al., 2019; Lv et al., 2021; Gao M. et al., 2022; Qiao et al., 2022; Xie et al., 2022; Liang et al., 2024c; Ma et al., 2024; Sun et al., 2024; Wang Y. et al., 2024). Ginsenosides can raise the abundance of Lactobacillus in the gut, enhance the activity of bile salt hydrolase (BSH), promote the hydrolysis and excretion of conjugated bile acids, and thereby inhibit the FXR-FGF15 signaling pathway in enterohepatic circulation (Wang Y. et al., 2024). This promotes the expression of cholesterol 7α-hydroxylase (CYP7A1) to accelerate cholesterol metabolism and decrease the serum levels of TC, TG, and LDL-C to improve dyslipidemia (Wang Y. et al., 2024). Ginsenosides directly induce the production of mucins, maintain the growth of the beneficial bacteria *Akkermansia muciniphila*, and increase the expression of TJPs to enhance the IMB, thereby reducing serum LPS concentration and mitigating systemic IR (Wang Y. et al., 2024). Lv et al. found that Ginkgolide B regulates the composition of the GM, particularly by increasing the abundance of *Bacteroides* and reducing the abundance of *Helicobacter*, significantly inhibiting the mRNA and protein expression of FMO3 to reduce the concentrations of TMA and TMAO, exerting anti-atherosclerotic effects (Lv et al., 2021). Xie et al. discovered that Ginsenoside Rc (GRc) may exert anti-atherosclerotic effects through the comprehensive effects by regulating the GM and fecal metabolites closely associated with cardiovascular diseases (Xie et al., 2022).

##### 4.3.3.7 Other natural compounds

Numerous natural compounds are extracted from nature, such as long-chain monounsaturated fatty acids (LCMUFAs) from fish oil, Bowman-Birk type major trypsin inhibitor from panicum millet bran (FMB-BBTI), Capsaicin, Eicosapentaenoic Acid-Enriched Phosphoethanolamine Plasmalogens (EPA-PlsEtns), Indole-3-carbinol (I3C), *Helianthus annuus* L., and Usnea ethanol extract (UEE), among others (Supplementary Table 8; Gautam et al., 2019; Ding et al., 2020; Li et al., 2020; Tsutsumi et al., 2021; Wang J. et al., 2021; Dai et al., 2022; Liu Y. J. et al., 2022; Shan et al., 2022; Wu et al., 2022; Jia A. et al., 2023; Liu R. et al., 2023; He et al., 2024). These natural compounds can exert anti-atherosclerotic effects by improving the composition of the GM, regulating microbial metabolites, reducing IR, improving LM, and protecting the function of the IMB (Gautam et al., 2019; Ding et al., 2020; Li et al., 2020; Tsutsumi et al., 2021; Wang J. et al., 2021; Dai et al., 2022; Liu Y. J. et al., 2022; Shan et al., 2022; Wu et al., 2022; Jia A. et al., 2023; Liu R. et al., 2023; He et al., 2024). He et al. were the first to find that I3C can decrease the abundance of *Bacilli* and *Lactobacillales* and raise the abundance of *Verrucomicrobia* and *Verrucomicrobiae* to regulate the composition and metabolic function of the GM, thereby inhibiting the progression of AS (He et al., 2024). FMB-BBTI can improve the structure and function of the GM by increasing the abundance of *Lactobacillus* and *Turicibacter*, promoting LM, and decreasing the levels of the major inflammatory cytokines TNF-α and IL-1β (Shan et al., 2022). EPA-PlsEtns can reduce the abundance of *Bacteroides* and increase the abundance of *Clostridium* to regulate the composition of GM, improve bile acid metabolism, and lower serum cholesterol levels, thereby significantly reducing the area of atherosclerotic lesions (Ding et al., 2020). EPA-PlsEtns can also inhibit the expression of the farnesoid X receptor (FXR), promoting the synthesis of bile acids and further reducing cholesterol accumulation (Ding et al., 2020). UEE can reduce the abundance of *Verrucomicrobiota* while increasing the abundance of *Bacteroidetes* to regulate the composition of the GM, lowering the serum levels of LPS, IL-6, TNF-α, TC, TG, and LDL-C, and increasing the serum level of HDL-C, thus improving LM and IR (Liu Y. J. et al., 2022). UEE can also upregulate the expression of TJPs -ZO-1 and occludin in the intestine, enhancing the integrity of the IMB (Liu Y. J. et al., 2022).

#### 4.3.4 Mechanism of TCM in treating AS

Ji et al. found that the GM can mediate the reduction of serum lipid and inflammatory cytokine levels in TCM formulations, thereby enhancing the therapeutic effects of TCM on AS (Supplementary Table 8; Ji et al., 2020). Blood-activating and stasis-resolving, heat-clearing and toxicity-relieving, qi-invigorating and surface-strengthening, tranquilizing and mind-stabilizing, and diuretic and dampness-removing classes of TCM can regulate the composition of the GM and GMMs, improve IR and LM, protect the integrity of the IMB, and exert therapeutic effects on AS (Zhu et al., 2020; Yang R. et al., 2021; Zhang et al., 2021a; Dongliang et al., 2022; Qi et al., 2022; Wang A. et al., 2022; Yang Q. et al., 2022; Fu et al., 2023; Liao et al., 2023; Wan et al., 2024; Yu et al., 2024; Yue et al., 2024). Naoxintong (NXT), an herb that activates blood circulation and resolves stasis, can reshape the GM in AS mice, regulate the levels of SCFAs, and maintain the stability of the intestinal barrier, thereby reducing LPS leakage to inhibit the activation of the TLR4 pathway in the liver, and consequently improving the systemic IR (Wan et al., 2024). Wan et al. further suggested that the acetic acid content in feces and the relative abundance of *Faecalibacterium* serve as potential therapeutic biomarkers for NXT treatment in AS (Wan et al., 2024). Qing-Xin-Jie-Yu Granule (QXJYG) alters GM by increasing *Roseburia* and *Aerococcus* while decreasing *Alistipes* and *Rikenella*, affecting bile acid metabolism and cholesterol synthesis by upregulating CYP7A1 and CYP27A1 and downregulating FGF15 and β-Klotho mRNA (Wang A. et al., 2022). Alisma orientalis Beverage (AOB) modifies GM, reducing FMO3 expression and circulating TMAO to inhibit inflammatory cytokine release, thus exerting anti-atherosclerotic effects (Zhu et al., 2020). Banxia Xiexin decoction potentially improves peripheral and brain LM by reducing *Proteobacteria* and *Deferribacteres*, aiding in the treatment of AS-related depression (Liao et al., 2023).

#### 4.3.5 The impact of various western medicines on AS

Various western medicines have been shown to influence the progression of AS through regulating the composition of the GM and GMMs (Supplementary Table 8; Shi et al., 2019; Kappel et al., 2020; Garshick et al., 2021; Yan et al., 2021; Bai et al., 2022; Li X. L. et al., 2022; Yang X. Y. et al., 2022; Bai et al., 2023; Hao et al., 2023; Tian et al., 2023; Traughber et al., 2023; Miao et al., 2024; Traughber et al., 2024). Bicyclol (BIC) can alleviate inflammatory responses and improve dyslipidemia by increasing the abundance of bacterial genera that produce SCFAs, such as *Clostridium*, *Bacteroides*, and *Ruminococcaceae* (Li X. L. et al., 2022). BIC is capable of upregulating the expression of TJPs in the intestinal epithelium (occludin and ZO-1), thereby protecting the integrity of the IMB and mitigating inflammation (Li X. L. et al., 2022). Li et al. also demonstrated the anti-AS effect of BIC mediated by the GM using FMT technology (Li X. L. et al., 2022). Disulfiram can improve the composition of GM by increasing the abundance of bacteria such as *Akkermansia* and reducing the abundance of *Romboutsia*, thereby mitigating inflammation and improving LM (Traughber et al., 2024). Hydroxyurea can regulate the composition of the GM by reducing the abundance of *Lactobacillus* and *Helicobacter* and increasing the abundance of *Lachnospiraceae_NK4A136* and *Lachnospiraceae_UCG-008*, thereby downregulating the expression of NPC1L1 in small intestinal epithelial cells to improve LM (Yang X. Y. et al., 2022). Ben Arpad Kappel et al. found that antibiotics can reduce the abundance of bacteria such as *Lachnospiraceae* to inhibit tryptophan metabolism and secondary bile acid metabolism, thereby promoting the development of AS (Kappel et al., 2020). Michael S. Garshick et al. discovered that antibiotics can decrease gut microbial diversity and increase the ratio of *F/B* to promote an increase in CD68-positive cell content and M1 polarization within AS plaques, thereby exacerbating IR (Garshick et al., 2021). Miao et al. have demonstrated that intermittent antibiotic treatment modifies the GM, thereby reducing IR and enhancing the integrity of the IMB (Miao et al., 2024). This intervention effectively mitigates AS in LDLR-/- hamster models subjected to high-fat high-cholesterol (HFHC) and high-cholesterol (HC) diets (Miao et al., 2024).

#### 4.3.6 Effects of exercise on AS

Studies have shown that endurance exercise can modulate specific microbial populations, such as *Desulfovibrio* and *Tyzzerella*, promoting the production of anti-inflammatory SCFAs, thereby alleviating AS progression (Supplementary Table 8; Huang W. C. et al., 2022). Milena Schönke et al found that evening exercise, compared to morning exercise, was more effective in reducing the development of AS, an effect that may be related to improvements in the GM, suggesting that the timing of exercise may have regulatory effects on the GM structure and the progression of AS (Schönke et al., 2023).

#### 4.3.7 The impact of other factors on AS

Studies have found that certain peptide substances can selectively reshape the composition of the GM to regulate the levels of SCFAs and bile acids, thereby inhibiting the production of pro-inflammatory cytokines, such as IL-6, TNF-α, and IL-1β, increasing the number of intestinal regulatory T cells, and enhancing the intestinal barrier, which contributes to the anti-atherosclerotic effect (Supplementary Table 8; Chen et al., 2020). Kim et al. used FMT from healthy donors to beneficially reshape the GM in treating AS in CTRP9-deficient mice (Kim et al., 2022). Human umbilical cord mesenchymal stem cells (HUCSCs) have the potential to ameliorate GMD induced by a high-fat diet (Li Y. H. et al., 2021). It achieve this by reducing the production of TMAO, thereby inhibiting the inflammatory progression and phagocytosis of oxidized low-density lipoprotein (ox-LDL), ultimately resulting in a decreased burden of atherosclerotic plaque (Li Y. H. et al., 2021). Among the included studies, various factors, such as environmental factors, plant bactericides, and immune deficiencies, have all been confirmed to promote the development of AS by regulating the structure of the GM and its metabolic products (Wu F. et al., 2019; Wu F. et al., 2019; Hu et al., 2021; Jin et al., 2021; Xue et al., 2021; Shi H. et al., 2022). Wu et al. found a significant correlation between the family *Aeromonadaceae* and the genus Citrobacter and intima-media thickness (IMT), as well as a significant interaction between Citrobacter and arsenic in water in IMT (Wu F. et al., 2019). This indicates that the GM has a significant role in the development of AS in populations exposed to high amounts of arsenic. Propamocarb exposure can increase the abundance of AS-associated GM, such as *Peptostreptococcaceae, Ruminococcaceae*, and *Clostridiales_VadinBB60_group*, causing GMD, thus promoting AS (Jin et al., 2021). Acrolein can mediate the activation of the MAPK pathway and downregulate Clock-Bmal1 in the process of AS, leading to the overexpression of MMP9 (Wu F. et al., 2019). The absence of IL-10 reduces GM diversity, especially by decreasing the number of beneficial bacteria and increasing the number of harmful bacteria, leading to an increase in the production of LPS, ultimately causing systemic inflammation (Shi H. et al., 2022). Intermittent hypoxia (IH) or intermittent hypercapnia (IC) can promote AS by changing GMMs, affecting IR and LM (Xue et al., 2021). Chronic Intermittent Hypoxia (CIH) increases the quantity of harmful bacteria, such as *Halomonas* and *Oceanospirillales*, and decreases the quantity of beneficial bacteria, such as *Sutterella*, leading to GMD (Hu et al., 2021). This inhibits microbial functions related to DNA replication, recombination, repair proteins, glycosaminoglycan biosynthesis and metabolism, and cofactor and vitamin metabolism, thereby exacerbating AS (Hu et al., 2021). Diesel exhaust particles (DEPs) reduced the alpha diversity of the GM and the levels of SCFAs but had no significant effect on the development of AS (van den Brule et al., 2021).

## 5 Limitations

During the scoping review, we recognized that the selection and evaluation of literature inevitably encountered subjective factors. Such interference may result in the overlooking of valuable findings or incorrect inclusion of lower-quality studies. Although we strive for objectivity and fairness, completely eliminating the influence of subjective bias is a challenging endeavor that could potentially affect the accuracy and reliability of the research outcomes. Moreover, the focus of this study on English-language literature may have neglected research from other languages or regions, possibly limiting the comprehensiveness and representativeness of this review. In particular, in interdisciplinary fields, the scoping review may not have covered all research methodologies and perspectives, particularly those that have not yet been thoroughly explored. Publication bias is a recognized concern in systematic reviews. Although we conducted a comprehensive search, we cannot completely rule out its influence.

The 192 studies included in this analysis demonstrated considerable heterogeneity in terms of experimental models (e.g., mouse genotypes such as ApoE-/-, Ldlr-/-, and hamsters; human studies encompassing various ethnicities and stages of disease), interventions (probiotic strains, drug dosages, and dietary components), and analytical methods (16S rRNA sequencing and metagenomics). This variability constrains the generalizability of mechanistic associations, thereby impeding the consensus on the roles of specific GM/GMMs across different models. For example, TMAO is known to promote AS in certain animal models; however, it exhibits inconsistent correlations in human studies. This inconsistency may arise from discrepancies between the models or inadequate control of host factors.

Most studies, particularly those involving human observational research, primarily report correlations between GM/GMMs and AS without providing direct causal validation. The “gut-vascular axis” hypothesis is currently supported by indirect evidence, highlighting the need for longitudinal cohort studies and mechanism-driven experiments, such as those utilizing germ-free animal models to establish causality. However, the present evidence does not rule out the influence of confounding factors, including dietary patterns, medication use, or genetic polymorphisms, which may simultaneously affect both GM composition and vascular pathology.

Interventions that demonstrate efficacy in animal models, such as probiotics and herbal formulations, lack sufficient validation in clinical settings. The regulatory mechanisms underlying individual variations, including baseline microbiota and genetic background, in response to interventions remain unexplored, thereby hindering the application of precision medicine in this field. Current evidence is inadequate to inform clinical guidelines, necessitating the verification of intervention efficacy and safety through large-scale randomized controlled trials and real-world data analyses.

## 6 Conclusion

In the realm of investigating the association between GM and cardiovascular diseases, current research indicates that an imbalance in GM has a close connection with the onset and progression of cardiovascular diseases. The GM contributes to the development of AS by regulating lipid levels, exacerbating IR, and disrupting microbial balance. In addition, the relationship between oral inflammation and GMD plays a significant role in the pathogenesis of AS. However, significant differences in the composition and function of GM among distinct populations may obscure the critical roles of specific bacterial strains or metabolites in the formation of AS. Studies have revealed that variations in microbial features and metabolic profiles between Chinese and Swedish populations contribute to discrepancies in AS-related biomarkers. The mechanisms by which GM regulates atherosclerotic plaque formation through the “gut-vascular axis” involve interactive pathways including immune metabolism, disordered lipid metabolism, and endothelial dysfunction. However, the synergistic or antagonistic interactions of these mechanisms remain poorly understood and require further in-depth research. Considering the substantial variations in GM composition and AS-related biomarkers among different populations, it is imperative to place greater emphasis on influential factors such as ethnic background, dietary habits, and geographical location to enhance the generalizability of research findings. Additionally, future research should focus more on the roles of the gut virome, mycobiome, and host genetics within the gut-vascular axis to enhance our understanding of the underlying mechanisms of GM.

**FIGURE 5:**
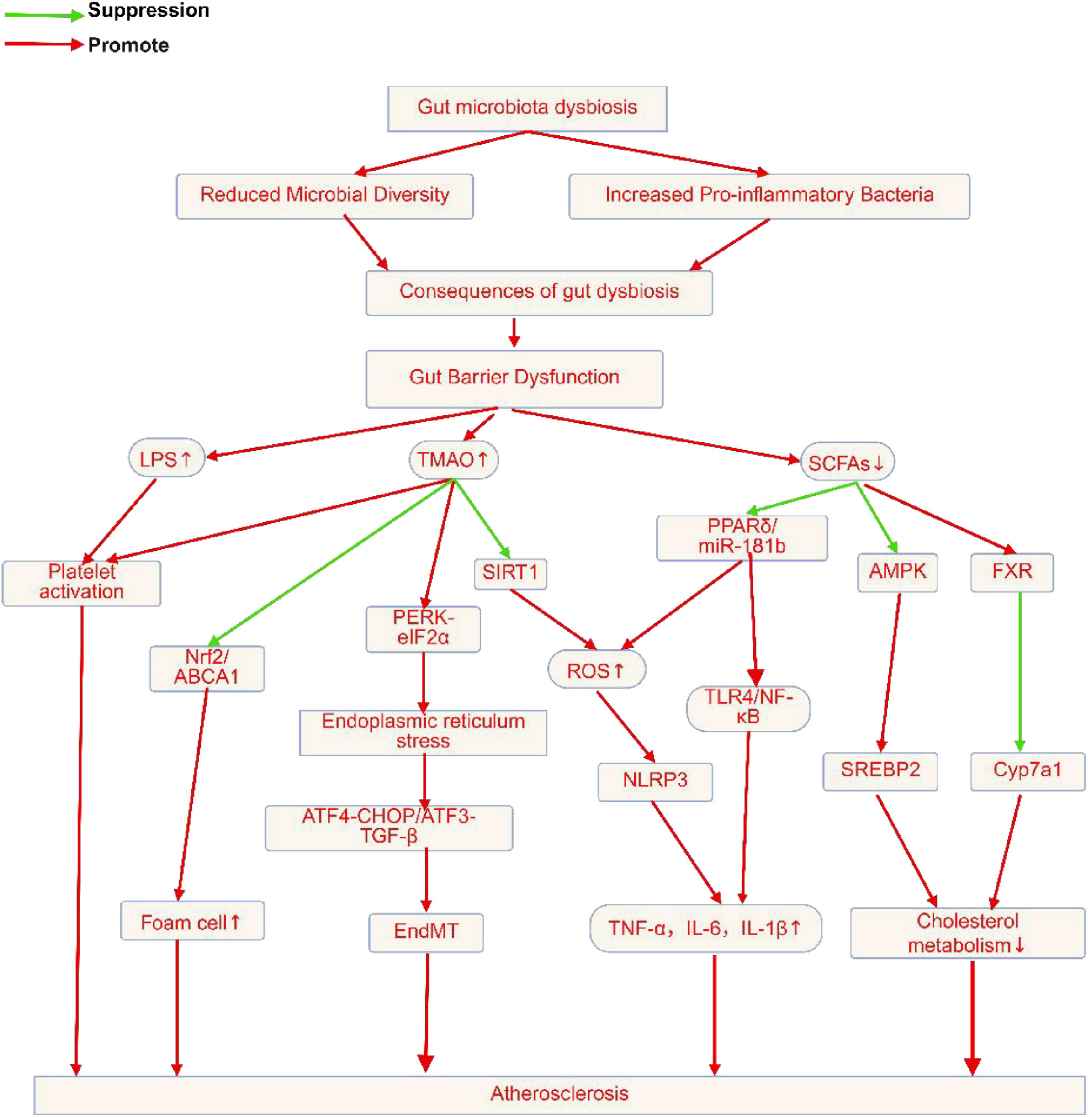
Conceptual diagram of the mechanism by which the gut-vascular axis promotes atherosclerosis. Created in BioRender. Dezhi (2025) (https://BioRender.com/wfg4i5z).

Studies suggest that intervention in the gut and oral microbiota may open new avenues for the treatment and prevention of cardiovascular diseases. For instance, TMAO exacerbates IR, affecting GM’s structure and thereby promoting the progression of AS. In contrast, microbial metabolites such as SCFA, IPA, glycine lipids, GCA, and urolithins can resist AS by improving LM, reducing inflammation, and maintaining gut barrier function. A recent study has revealed that TMAO significantly enhances macrophage polarization toward the pro-inflammatory M1 phenotype by upregulating the expression of methyltransferase Mettl3 and suppressing IRAK-M expression (Wen et al., 2025). In the inflammatory environment of myocardial infarction, the number of hematopoietic progenitor cells in the spleen significantly increased, and a large number of monocyte precursors were produced through peripheral hematopoiesis, thereby exacerbating AS (Dutta et al., 2012). TMAO activates the PERK-eIF2α pathway, a critical signaling cascade associated with endoplasmic reticulum stress (Bingyu et al., 2024). This pathway differentially regulates the transcription factors ATF4-CHOP and ATF3-TGF-β signaling networks, thereby synergistically inducing endothelial-mesenchymal transition (EndMT) and cellular apoptosis (Bingyu et al., 2024). Notably, TMAO also disrupts the dynamic balance of the Nrf2/ABCA1 pathway, exacerbating oxidative stress and lipid accumulation in macrophage-derived foam cells, thereby establishing a pro-atherosclerotic vicious cycle (Luo K. et al., 2024). SCFAs alleviate vascular inflammation by modulating epigenetic regulation through histone deacetylase (HDAC) inhibition, promoting regulatory T cell (Treg) differentiation, and suppressing pro-inflammatory cytokines (IL-6, TNF-α) (Dicks, 2024; Matacchione et al., 2024; Chulenbayeva et al., 2025). The interaction between the spleen and liver reinforces prior discoveries, with the liver-spleen axis integrating metabolic disturbances present in AS with immune-inflammatory modifications (Tarantino et al., 2021). This review also developed a concept map of the mechanism by which the gut-vascular axis promotes atherosclerosis (Figure 5). These findings not only deepen our understanding of the pathophysiological mechanisms underlying the “gut-vascular axis” but also provide crucial theoretical support for developing precision intervention strategies targeting this axis. GM-derived metabolites, such as TMAO and SCFAs, independently influence AS (Muttiah and Hanafiah, 2025). Their production is regulated by an imbalanced gut microbiota composition, suggesting that alterations in these ratios may indirectly drive the pathological mechanisms of distal AS mediated by metabolites (Muttiah and Hanafiah, 2025). This underscores the importance of monitoring GM composition as both a risk assessment marker for AS and a potential therapeutic intervention target (Gan et al., 2024b). Targeted regulation of the TMAO/SCFAs metabolic axis and its homeostasis may represent a novel strategy for AS intervention in the future.

This review identified five clinical translation-related studies and summarized the drugs/biomarkers, limitations, and corresponding solutions identified in these studies (Supplementary Table 9; Liu S. et al., 2020; Baragetti et al., 2021; Tsutsumi et al., 2021; Bai et al., 2022; Yang et al., 2024). Current evidence is primarily associative, and future studies should prioritize longitudinal and interventional studies to establish the causality between specific GM/GMMs changes and AS outcomes. While this review details the known pathways (e.g., TMAO and SCFAs), the synergistic or antagonistic interactions among various mechanisms (immune, metabolic, and endothelial) remain poorly understood and warrant further investigation. Future studies should also be devoted to elucidating the specific mechanisms by which GM/GMMs affect AS, as well as the interactions and regulatory roles of GMMs with GM.

Additionally, investigating the roles of probiotics, prebiotics, diet, natural compounds, medications, and exercise in regulating IR, LM, and gut barrier function will aid in the progression of new AS prevention and treatment strategies based on the GM and GMMs. These interventions (e.g., natural products and FMT) show promise in animal models but lack robust clinical validation in humans. This review advocates for large-scale randomized controlled trials to assess human efficacy and safety. Validation of the efficacy and safety of these natural compounds and probiotics in the treatment and prevention of AS in individuals, as well as investigating the impact of different types, durations, and intensities of exercise on GM and the AS process, will be key directions for future research. It is necessary to test synbiotics in high TMAO patients in the future. The long-term safety, optimal dosing, and combinatorial effects of interventions require systematic evaluation, such as symbiotics and the combination of diet and exercise. Simultaneously, a thorough analysis of the interactions between environmental factors, diet, lifestyle, and GM is an important research area worthy of further exploration. The gut-vascular axis mediates the development and progression of AS. However, this process is also modulated by additional AS risk factors, including environmental exposure, lifestyle, sleep disturbances, and stress. Future research should utilize multifactorial models to further elucidate the associations and causal relationships between these factors. Finally, standardized protocols for GM analysis and AS evaluation would enhance both comparability and the potential for meta-analyses. This review advocates for future GM studies to adopt the STORMS framework to standardize research design, animal models, sequencing methodologies, and outcome measurements (Mirzayi et al., 2021).

Concerns regarding the safety of microbial therapy encompass various aspects, including adverse events and regulatory challenges. Research has indicated that the safety and precision of FMT remain unpredictable in clinical settings (Li et al., 2024c). Post-FMT adverse events ranged from mild-to-moderate (e.g., abdominal pain, flatulence, increased stool frequency, vomiting, fever) to severe complications including aspiration and intestinal perforation. The safety concerns of microbiota modulation deserve attention (Gulati et al., 2023). Immunocompromised individuals (e.g., chemotherapy patients, transplant recipients) face heightened risks from probiotics. Some users may experience mild gastrointestinal symptoms like bloating or gas during initiating probiotic supplementation (Haranahalli Nataraj et al., 2024). Traditional approaches to modulating intestinal microbiota present uncertainties in safety and accuracy, with a lack of comprehensive long-term efficacy and safety data, particularly concerning suboptimal delivery systems that may increase therapeutic risk (Li et al., 2024c; Hamza et al., 2025). Regulatory frameworks require enhancement, as demonstrated by the need for more rigorous standard of FMT quality and safety standards in the United States and Europe to ensure treatment consistency and reliability (Hoffmann et al., 2025). Ultimately, addressing these safety issues depends on optimizing the delivery systems, improving long-term monitoring, and developing stringent regulatory mechanisms.

AI has demonstrated remarkable technological breakthroughs and clinical value in disease diagnosis and prediction. In addressing class imbalance issues in rare disease diagnosis, generative adversarial network (GAN)-synthesized retinal disease data has significantly improved the accuracy of hereditary retinal disease classification models, validating the effectiveness of synthetic data in mitigating diagnostic bias (Veturi et al., 2023). AI models utilizing real-world data have established diabetes complication prediction systems capable of early risk stratification for six complications including gestational diabetes and retinopathy (Huang J. et al., 2023). In ischemic stroke clinical applications, AI technology surpasses traditional scoring systems in predictive performance across multiple clinical scenarios such as functional recovery forecasting, cerebral edema risk warning, and etiology classification, through integration of imaging features, biomarkers, and clinical parameters (Heo, 2024). In the field of diagnostic medicine, artificial intelligence technologies, including machine learning and deep learning, facilitate precise risk stratification and disease detection by analyzing imaging data, such as MRI images (Hamm et al., 2023). This capability is evidenced by improved diagnostic accuracy and interpretability, particularly in the context of prostate-cancer diagnosis (Hamm et al., 2023). AI has demonstrated potential in uncovering associations within multi-omics data. For example, random forest algorithms have been utilized to identify characteristic microbial communities, specifically Clostridia, and metabolic biomarkers, such as citral, in patients with metabolic-associated fatty liver disease (MAFLD) complicated by cardiovascular risk (Li et al., 2024e). Additionally, these algorithms have been employed to predict clinical parameters (Li R. J.et al., 2021). Network modeling techniques have further elucidated three-dimensional interaction modules among microbiota, metabolites, and clinical parameters, particularly emphasizing the robust interplay between gut microbiota and urinary metabolomes (Li R. J.et al., 2021; Li et al., 2024e). Current research indicates that the efficacy of artificial intelligence in the prevention of cardiovascular diseases requires further validation through forthcoming clinical trials (El Sherbini et al., 2024). Currently, it has not been established as a reliable therapeutic intervention (El Sherbini et al., 2024). With the anticipated advancement in the comprehensive study of the gut virome, mycobiome, and other intestinal microorganisms, databases related to GM, such as GutMetaNet and gutMEGA, are expected to undergo continuous enhancement. By integrating multi-omics methodologies, including spatial metabolomics and single-cell transcriptomics, specific GM and GMMs will be identified as biomarkers for AS. Subsequently, AI will be utilized to amalgamate these specific biomarkers with AS-related influencing factors to develop clinical prediction models (Sud et al., 2023). The predictive validity of these models requires verification through extensive population-based studies. Resolving these challenges is expected to enable the personalized diagnosis and treatment of AS in the future.

## Footnotes

1
www.biologistics.com.cn

2
www.biorender.com
