# Supplementary material for: Gut microbiota regulate atherosclerosis via the gut-vascular axis: a scoping review of mechanisms and therapeutic interventions
Source: Front Microbiol. 2025 Aug 8;16:1606309. doi: 10.3389/fmicb.2025.1606309 (PMC12370700; doi:10.3389/fmicb.2025.1606309)
Supplement: Supplementary file 1 [file Data_Sheet_1.pdf]

Supplementary Materials 1：Screening Process of Literature Selection.

Step 1: Two researchers eliminated 1,749 duplicate studies from the initial 3,556 imported articles through a two-step procedure: ① automated removal of 1,518 duplicates using Endnote software; ② independent manual screening of titles and abstracts, followed by a consensus discussion to remove an additional 231 duplicates.

Step 2: Two researchers independently screened the titles and abstracts against predefined inclusion and exclusion criteria, followed by cross-checking. Discrepancies were resolved by a third researcher, resulting in the exclusion of 1,505 articles from the review. The exclusion criteria were as follows: ① 68 studies excluded due to incompatible document types; ② 452 studies excluded for irrelevant research topics; ③ 519 review articles excluded; ④ 466 studies excluded for being outside the defined publication time frame.

Step 3: Full-text retrieval was attempted for the remaining 302 records. After discussion and verification by the third researcher, 18 articles without accessible full texts were excluded from the study.

Step 4: Two researchers independently conducted a full-text review of the 284 articles. Following a reappraisal of the inclusion and exclusion criteria and arbitration by the third researcher, 92 articles were excluded due to thematic incompatibility.

Step 5: The final 192 articles underwent iterative independent verification by two researchers, cross-checking, and third-party validation of the data. All three researchers confirmed the consensus on the final inclusion of 192 articles through a structured discussion.

Supplementary Table 1:The search strategy.

Search Strategies for Web of Science Database

Search strategy	searches	Number of obtained literature
#1	(((((TS=("Gastrointestinal Microbiome")) OR TS=("gut microbiota")) OR TS=(Intestinal Microbiome")) OR TS=("gut microbiome"))OR TS=("Intestinalmicrobiota")) OR TS=("Gastrointestinal Microbial Communities")	97614
#2	(TS=("atherosclerotic cardiovascular disease")) OR TS=("atherosclerosis")	100810
#3	#1 AND #2	1485

Embase Database Retrieval Strategy

Search strategy	searches	Number of obtained literature
#1	('gastrointestinal microbiome': ti, ab, kw OR 'gut microbiota': ti, ab, kw OR 'intestinal microbiome': ti, ab, kw OR 'gut microbiome':ti.ab, kw OR 'intestinal microbiota' ti ab. kw OR 'gastrointestinal microbial communities': ti, ab, kw)AND [2014-2024]/py	84678
#2	('atherosclerotic cardiovascular disease': ti, ab, kw OR atherosclerosis: ti, ab, kw)AND [2014-2024]py	102291
#3	#1 AND #2	1113

Cochrane Library Database Retrieval Strategies

Search strategy	searches	Number of obtained literature
#1	("Gastrointestinal Microbiome"):ti, ab, kw OR ("gut microbiota") ti, ab, kw OR ("Intestinal Microbiome"):ti, ab, kw OR ("gut microbiome"):ti, ab, kw OR ("Intestinal microbiota" OR "Gastrointestinal Microbial Communities"):ti,ab,kw with Publication Year from 2014 to 2024, with Cochrane Library publication date from Jan 2014 to Jul 2024, in Trials	6770
#2	("atherosclerotic cardiovascular disease") ti, ab, kw OR ("atherosclerosis") ti,ab,kw with Publication Year from 2014 to 2024, with Cochrane Library publication date from Jan 2014 to Jul 2024, in Trials	5831
#3	#1 AND #2 with Publication Year from 2014 to 2024, with Cochrane Library publication date from Jan 2014 to Jul 2024, in Trials	53

Supplementary Table 2:This table presents the main characteristics of studies on the impact of GM on AS..

Title	Author	Time	Research object	Countries included in the population	Sequencing object	Research aim	Main summary
Alterations in the gut mycobiome with coronary artery disease severity	Kun An et al	2024	Human	China	16SrDNA	To investigate the potential association of the gut mycobiome with CAD, this study conducted a comprehensive analysis of the gut microbial profile in 101 CAD patients and 31 healthy controls using high-throughput sequencing.	In conclusion, the structure and composition of the gut fungal community differed from healthy controls to various subtypes of CAD, revealing key fungi taxa alterations linked to the onset and progression of CAD.
Candida albicans accelerates atherosclerosis by activating intestinal hypoxia-inducible factor2a signaling	Xuemei Wang et al	2024	Human Apoe -/- mice	China	Metagenomics	However, to date, there is still little known about the role of gut fungi and their metabolites in metabolic diseases, such as atherosclerosis.	This study findings identify a role for intestinal fungi in atherosclerosis progression and highlight the intestinal HIF-2a-ceramide pathway as a target for atherosclerosis treatment.
Low-grade endotoxaemia enhances artery thrombus growth via Toll-like receptor 4: implication for myocardial infarction	Roberto Carnevale et al	2020	Human C57BL6J mice	Italy	Nothing	Low-grade endotoxaemia is detectable in human circulation but its role in thrombosis is still unclear.	ST-elevation myocardial infarction patients disclose an enhanced gut permeability that results in LPS translocation in human circulation and eventually thrombus growth at site of artery lesion via leucocyte–platelet interaction.
Gut Microbiome Alterations in Patients With Carotid Atherosclerosis	Jingfeng Chen et al	2021	Human	China	Whole-genome shotgun	This study investigated the relationships between the gut microbiome and CAS in a cross-sectional cohort to further provide a theoretical basis to prevent and decrease the risk of CAS in China.	This study results indicate the existence of a cyclic pathway that elevates the circulating concentrations of trimethylamine-N-oxide in patients with CAS but reduces its concentrations in healthy controls.
Alterations in gut microbiota and host transcriptome of patients with coronary artery disease	Liuying Chen et al	2023	Human	China	16SrRNA	This study aims to uncover the synergistic effects of host genes and gut microbes associated with CAD through integrative genomic analyses.	This study highlight that dysregulated gut microbes contribute risk to CAD by interacting with host genes. These identified microbes and interacted risk genes may have high potentials as biomarkers for CAD.

Co-toxicity of Endotoxin and Indoxyl Sulfate, Gut-Derived Bacterial Metabolites, to Vascular Endothelial Cells in Coronary Arterial Disease Accompanied by Gut Dysbiosis	Marcin Choroszy et al	2022	Human Human umbilical vein endothelial cells Human monocytic THP-1 cell line	Poland	16SrRNA	The study aimed to research whether gut dysbiosis can increase bacterial metabolites concentration in the blood of CAD patients and what impact these metabolites can exert on endothelial cells.	16SrRNA sequencing analysis revealed gut dysbiosis in CAD patients that was further confirmed by elevated levels of bacterial metabolites, i.e., LPS and indoxyl sulfate in patientssera. Both metabolites demonstrated co-toxicity in meager concentrations to endothelial cells inducing ROS, E-selectin, and MCP-1 production and promoting thrombogenicity of endothelial cells confirmed by monocyte adherence.
Distinct gut microbiota signatures associated with progression of atherosclerosis in people living with HIV	Mar Masiá et al	2024	Human	Spain	16SrRNA	This study prospectively examined the relationship between the longitudinal dynamics of microbiota composition and the progression of subclinical atherosclerosis.	Progression of atherosclerosis in PWH might be associated with distinctive signatures in the gut microbiota.
Association between Small Intestinal Bacterial Overgrowth and Subclinical Atheromatous Plaques	Changhao Dong et al	2022	Human	China	Nothing	The aim of this study was to investigate the frequency of subclinical atheromatous plaques in patients with SIBO and to explore the association between these two conditions.	SIBO was found to be associated with subclinical atheromatous plaques, and the mechanism of this association warrants further exploration.
Gut Microbiota and Coronary Plaque Characteristics	Akihiro Nakajima et al	2022	Human	Japan	16SrRNA	This study was conducted to investigate the relationship between gut microbiota and coronary plaque characteristics in patients with coronary artery disease.	In summary, this study found bacteria that are associated with ACS, and favorable and unfavorable coronary plaque characteristics. Overall, this study findings show gut microbiota may play an important role in vulnerable plaque formation.
Is there an association between atherosclerotic burden, oxidative stress and gut-derived lipopolysaccharides?	Lorenzo Loffredo et al	2020	Human	Moldova	Nothing	The aim of this study was to assess serum LPS as well as oxidative stress in PAD patients and controls . Furthermore, this study wanted to analyze the relationship between LPS and the severity of atherosclerosis in the lower limb arteries.	This study suggest that LPS is elevated in PAD patients with a close association with the atherosclerotic burden and oxidative stress. The correlation between LPS and zonulin suggests changes in gut permeability could be a potential trigger of LPS translocation in the peripheral circulation.
Exploration of Crucial Mediators for Carotid Atherosclerosis Pathogenesis Through Integration of Microbiome, Metabolome, and Transcriptome	Lei Ji et al	2021	Human	China	16SrDNA	Taken together, both human and animal studies have indicated that alterations of the gut microbiota and plasma metabolites might be involved in the progression of AS, but the details of these alterations in patients	Through an integrated analysis of multi-omics, this study explored the possible “microbiota–metabolite–gene” regulatory axis that may act on CAS, thereby helping to establish a theoretical basis for the further specialized study of

						with CAS have not been fully characterized.	CAS.
Comparison of thrombus, gut, and oral microbiomes in Korean patients with ST-elevation myocardial infarction: a case–control study	Ju-Seung Kwun et al	2020	Human	Korea	Metagenomic	This study aimed to investigate (1) the coronary thrombus microbiome and (2) its composition relative to the gut and oral microbiomes of STEMI patients relative to those of healthy individuals.	The study results indicate that the relative abundance of the gut and oral microbiomes was correlated with that of the thrombus microbiome.
Characteristics of the gut microbiota of patients with symptomatic carotid atherosclerotic plaques positive for bacterial genetic material	Hang Lv et al	2023	Human	China	16SrDNA	This study, based on the detection of bacterial DNA in carotid plaques, explores the characteristics of GM in SCAS patients with plaque bacterial genetic material positivity, aiming to provide areference for subsequent research.	In summary, the study is the first to identify significant alterations in the gut microbiota of patients with positive plaques, providing crucial microbial evidence for further exploration of the pathogenesis of SCAS.
Dysbiosis of Gut microbiota in patients with Large-Artery Atherosclerotic Stroke: a pilot study	Chatpol Samuthpongtorn et al	2023	Human	Thailand	16SrRNA	Therefore, the purpose of the study is to establish an association between the changes in gut microbiota and ischemic stroke patients, with a particular emphasis on LAA subtypes due to their high prevalence, in order to shed light on how gut microbiota may be a potential therapeutic target for ischemic stroke.	Patients with large-artery atherosclerotic stroke had a decreased microbiome beta-diversity and certain gut microbiota genera may be related to large-artery atherosclerotic stroke.
Streptococcus species abundance in the gut is linked to subclinical coronary atherosclerosis in 8973 participants from the SCAPIS cohort	Sergi Sayols-Baixeras et al	2023	Human	Sweden	Shotgun metagenomics	This study aimed to identify associations between the gut microbiome and computed tomography-based measures of coronary atherosclerosis, and to explore relevant clinical correlates.	This study provides evidence of an association of a gut microbiota composition characterized by increased abundance of *Streptococcus spp*. and other species commonly found in the oral cavity with coronary atherosclerosis and systemic inflammation. Further longitudinal and experimental studies are warranted to explore the potential implication of a bacterial component in atherogenesis.

Structural changes in the gut virome of patients with atherosclerotic cardiovascular disease	Youshan Li et al	2024	Human	European Bioinformatics Institute database	Metagenomic	The gut virome was profiled from fecal metagenomes and compared between patients and healthy controls, which revealed numerous viral compositional and functional signatures associated with ACVD. Moreover, the ability of viral signatures to classify ACVD patients and healthy controls was also explored.	This study results provide a comprehensive view of the ACVD gut virome, which may contribute to the development of novel diagnostic and therapeutic strategies for ACVD and additional relevant cardiovascular diseases.
Association between Gut Microbial Diversity and Carotid Intima-Media Thickness	Helga Szabo et al	2021	Human	Hungary	16SrRNA	This study aimed to find associations between intestinal microbiome diversity and a marker of subclinical atherosclerosis, the carotidintima-media thickness (IMT).	The determining role of individual genera and their proportions in the development and progression of atherosclerosis can be assumed. Further studies are needed to clarify if these findings can be used as potential therapeutic targets.
The Relationship between Atherosclerosis and Gut Microbiome in Patients with Obstructive Sleep Apnoea	Helga Szabo et al	2022	Human	Hungary	16SrRNA	This study’s aim was to explore the relationship between adult OSA patients’ gut microbiome and atherosclerosis which is essential to find gut dysbiosis-targeted treatments that may be used as an adjunct to traditional OSA treatment and may result in advantages for OSA morbidities.	This is the first pilot research to analyze the association between the gut microbiome and atherosclerosis in adult patients with OSA with and without carotid atherosclerosis. Dysbiosis and individual bacteria may contribute to the development of carotid atherosclerosis in patients with OSA. Further investigations are necessary to reveal a more precise background in a larger sample.
Coronary artery disease isassociated with an altered gut microbiome composition	Takumi Toya et al	2020	Human	America	16SrDNA	This study performed the current study to investigate the composition and to infer functional differences in the gut microbiome of patients with CAD using 16S ribosomal DNA (rDNA) microbiome analysis.	The observed differences in taxa between CAD patients and controls in this study may provide insight into the link between the gut microbiome and CAD.

Age-dependent association of gut bacteria with coronary atherosclerosis:Tampere Sudden Death Study	Sari Tuomisto et al	2019	Human	Finland	PCR	The aim of this study was to investigate age-dependent changes in the major populations of the intestinal microbiome and their possible association with atherosclerotic severity and death due to CHD. Furthermore, studied whether such intestinal derived bacterial DNA can be found in coronary plaques, which could indicate the translocation of gut bacteria via the portal vein into circulation and further into coronary plaques.	DNA of the *Clostridium leptum group* and pathogenic *Enterobactericeae* increase in the gut microbiome with age and can be detected in the same individual's coronary plaques along with pathogenic *Streptococcus spp.*, associating with more severe coronary atherosclerosis.
Gut microbiota, circulating inflammatory markers and metabolites, and carotid artery atherosclerosis in HIV infection	Zheng Wang et al	2023	Human	America	Shotgun metagenomics	This study aimed to identify gut microbiota features focusing on bacterial species and functional components, measured using shotgun metagenomics sequencing, and serum proteomic inflammatory markers, measured by a proteomic platform of 92 proteins,associated with carotid artery plaque in women living with or at risk of HIV from the Women's Interagency HIV Study (WIHS). In addition, this study also related gut microbiota features with host circulating inflammatory markers and metabolites to explore potential mechanisms underlying the relationship between gut microbiota and atherosclerosis in the context of HIV infection.	Among women living with or at risk of HIV, this study identified several gut bacterial species and a microbial metabolite ImP associated with carotid artery atherosclerosis, which might be related to host immune activation and inflammation.

Apoe -/- mice: Apolipoprotein E knockout mice, 16SrDNA: 16S Ribosomal DNA, 16SrRNA: 16S Ribosomal RNA, PCR: Polymerase Chain Reaction, CAD: Coronary Artery Disease, PAD: Peripheral Arterial Disease, STEMI: ST-Elevation Myocardial Infarction, SCAS: Stable Coronary Artery Disease, LAA: Large-Artery Atherosclerotic, ACVD: Atherosclerotic Cardiovascular Disease, OSA: Obstructive Sleep Apnea, CHD: Coronary Heart Disease, HIV: Human Immunodeficiency Virus, CAS: Carotid atherosclerosis, GM: Gut microbiota ,HIF-2a: Hypoxia Inducible Factor 2 Alpha, LPS: Lipopolysaccharide, ROS: Reactive Oxygen Species, E-selectin: Endothelial Cell Selectin, MCP-1: Monocyte Chemoattractant Protein-1, SIBO: Small Intestinal Bacterial Overgrowth, ACS: Acute Coronary Syndrome, PWH: people living with HIV, WIHS: Women's Interagency HIV Study.

Supplementary Table 3: This table presents the main characteristics of studies on the impact of GM on AS.

Title	Author	Time	Research object	Sex	Sequencing method	Research aim	Main summary
Both gut microbiota and cytokines act to atherosclerosis in ApoE-/- mice	Qiuxia Liu et al	2020	C57BL/6J mice ApoE-/- mice	Male	16SrDNA	This study was to characterize the combined profiles of microbiomes and cytokines between ApoE-/- mice (a widely used mouse model for atherosclerosis) and WT mice that were fed with HFD.	Gut microbiota changes of the mice having atherosclerosis and their relationship with the inflammatory status could be one of the major etiological mechanisms underlying atherosclerosis.
A Proinflammatory Gut Microbiota Increases Systemic Inflammation and Accelerates Atherosclerosis	Eelke Brandsma et al	2019	Ldlr−/− mice	Female	16SrDNA	This study investigated whether a proinflammatory microbiota from Caspase1−/− (Casp1−/−) mice accelerates atherogenesis in Ldlr−/− mice.	Introduction of the proinflammatory *Casp1−/−* microbiota into Ldlr−/− mice enhances systemic inflammation and accelerates atherogenesis.
Chronic apical periodontitis exacerbates atherosclerosis in apolipoprotein E-deficient mice and leads to changes in the diversity of gut microbiota	Guowu Gan et al	2022	ApoE−/− mice	Male	16SrDNA	To investigate the impact of CAP on atherosclerosis and gut microbiota by establishing a *P. gingivalis* induced CAP in an ApoE−/− mice model.	In a mouse experimental model, pulp infection with *P. gingivalis* -induced CAP, thus aggravating the development of atherosclerosis. Meanwhile, CAP increased alpha diversity and altered the beta diversity of the gut microbiota.
Unveiling the oral-gut connection: chronic apical periodontitis accelerates atherosclerosis via gut microbiota dysbiosis and altered metabolites in apoE−/− Mice on a high-fat diet	Guowu Gan et al	2024	ApoE−/−mice	Male	PCR	The aim of this study was to explore the impact of CAP on atherosclerosis in apoE−/− mice fed HFD.	These findings emphasize the importance of maintaining good oral hygiene as a potential preventive measure against cardiovascular issues, as well as the need for further investigations into the intricate mechanisms linking oral health, gut microbiota, and metabolic pathways in CVD development.
Gut microbiota may mediate the impact of chronic apical periodontitis on atherosclerosis in apolipoprotein E-deficient mice	Guowu Gan et al	2023	ApoE−/− mice	Male	PCR	The study hypothesized that the gut microbiota could mediate the association between CAP and atherosclerosis.	In conclusion, this study shows that CAP-induced alterations in the gut microbiota can induce the development of atherosclerosis, suggesting that the gut bacterial pathway plays an important role in the association between CAP and atherosclerosis.

Helicobacter pylori Infection Acts Synergistically with a High-Fat Diet in the Development of a Proinflammatory and Potentially Proatherogenic Endothelial Cell Environment in an Experimental Model	Agnieszka Krupa et al	2021	Himalayan guinea pigs<br>Human umbilical vein endothelial cells<br>Human monocyte leukemic cell line	Male and Female	Nothing	The aim of this study is to explore the link between persistent HP infection and a high-fat diet in the development of proinflammatory conditions that are potentially proatherogenic.	These preliminary results support the hypothesis that HP antigens act synergistically with a high-fat diet in the development of proatherogenic conditions.
Oral *Porphyromonas gingivalis* infection affects intestinal microbiota and promotes atherosclerosis	Sowon Park et al	2023	ApoE- / - mice	Male	16SrRNA	The link between periodontitis and intestinal dysbiosis, two factors that contribute to atherosclerosis, has not been clearly defined. This study investigated the integrative effects of oral infection with *PG*, the major pathogen for periodontitis, on intestinal microbiota and atherosclerosis.	Oral PG infection promotes atherosclerosis and induces significant metabolic changes, including reduced serum HDL and reduced hepatic SR-B1 and ABCA1 expression, as well as changes in intestinal microbiota. This study suggests that intestinal dysbiosis accompanies periodontitis and could play a role in atherosclerosis.
Bacteroides fragilis Supplementation Deteriorated Metabolic Dysfunction, Inflammation, and Aorta Atherosclerosis by Inducing Gut Microbiota Dysbiosis in Animal Model	Guoxiang Shi et al	2022	Aope-/- mice	Male	PCR	The study aimed to explore whether and how BFS affects the diet, metabolic functions, intestinal environment, inflammation, and atherosclerotic lesion in the aorta of mice.	Conclusively, this study demonstrated for the first time that BFS by gavage induced gut microflora dysbacteriosis, which was characterized by decreased LAC abundance and increased DSV abundance. This condition led to the dramatic deterioration of glucose and lipid metabolic disorders and inflammation. This phenomenon aggravated vascular plaque formation and atherosclerotic progression in the mice animal model.
The Microbiota Promotes Arterial Thrombosis in Low-Density Lipoprotein Receptor-Deficient Mice	Klytaimnistra Kiouptsi et al	2019	Ldlr -/ -mice	Male and Female	16SrDNA	The possible contribution of commensal microbiota to Western diet-induced carotid artery atherosclerosis and plaque thrombogenicity in the Ldlr- /- mouse model is unknown. To explore whether the microbiota is involved in atherothrombosis, the study comparatively analyzed the plasma lipoprotein profile, vascular inflammation, carotid artery plaque size, extent of plaque rupture-induced thrombosis, and ex vivo platelet adhesion to collagen under flow in GF Ldlr- /- and CONV-R Ldlr- /- mice, receiving a normal chow control diet (CD) or a high-fat Western diet (HFD) for 16 weeks, respectively.	In conclusion, the study data demonstrate that the microbiota does not modulate late absolute atherosclerotic lesion size in the carotid artery, thus supporting recent reports using the Apoe- /- mouse model. Here the study confirmed in the Ldlr- /- mouse hypercholesterolemia model that the gut microbiota reduces plasma cholesterol levels with CD feeding, but not under HFD-induced hypercholesterolemia in the Ldlr- /- mouse model. The study results demonstrate that, despite not affecting the absolute lesion area in the carotid artery, the commensal microbiota augments low-grade inflammation in the vessel wall. The study results suggest that a diminished adhesiondependent platelet activation on type I and type III collagen

							causes reduced plaque rupture atherothrombosis in the carotid artery of HFD-fed GF Ldlr- / - mice. Future experiments should provide mechanistic insights on how the gut microbiota interferes with platelet-collagen interaction, the pivotal pathomechanism in arterial thrombosis.
The Role of Intestinal Dysbacteriosis Induced Arachidonic Acid Metabolism Disorder in Inflammaging in Atherosclerosis	Yingxin Sun et al	2021	ApoE-/mice C57BL/ 6J mice	Male	16SrRNA	To explore the underlying mechanism of how aging aggravates AS advancement.	Aging not only altered the gut microbiome community but also substantially disturbed metabolic conditions. This study results confirm that AA metabolism is associated with the imbalance of the intestinal flora in the AS lesions of aged mice. These findings may offer new insights regarding the role of gut flora disorders and its consequent metabolite changed in inflammaging during AS development.
Experimental Periodontitis Deteriorated Atherosclerosis Associated with Trimethylamine N-Oxide Metabolism in Mice	Lingling Xiao et al	2021	ApoE−/− mice C57BL/6J mice HepG2 cell	Male	16SrRNA	This study hypothesized that periodontitis may induce gut dysbiosis and abnormal hepato-intestinal metabolism, leading to the accelerated development of atherosclerosis.	This study discovered that experimental periodontitis in ApoE−/− mice induced gut dysbiosis and an increase in TMAO. These results suggest a possible mechanism by which periodontitis may accelerate atherosclerosis by influencing the intestinal microbes and the metabolism, which were triggered by inflammation of the liver and intestine.
Desulfovibrio desulfuricans aggravates atherosclerosis by enhancing intestinal permeability and endothelial TLR4/NF-kB pathway in Apoe / mice	Kun Zhang et al	2023	Apoe-/-mice Caco-2 cells Human intestinal epithelial cells	Male	16SrRNA	This study investigated the effects of *D. desulfuricans* on the atherosclerosis of Apoe-/-mice.	This study results demonstrate that *D. desulfuricans* can enhance the development of atherosclerosis by increasing intestinal permeability and host inflammatory response.

ApoE-/- mice: Apolipoprotein E knockout mice, Ldlr−/− mice: Low-density lipoprotein receptor knockout mice, 16SrDNA: 16S ribosomal DNA, PCR: Polymerase Chain Reaction, 16SrRNA: 16S ribosomal RNA, TMAO: Trimethylamine N-oxide, GF: Germ-Free, CAP: Chronic apical periodontitis, SR-B1: Scavenger Receptor Class B Type 1, ABCA1: ATP-binding cassette transporter A1, HP: Helicobacter pylori, HDL: High-Density Lipoprotein,CVD: Cardiovascular Disease,CAP: Chronic apical periodontitis, *PG: Porphyromonas gingivalis ,Casp1−/−: Caspase1−/−, LAC: Lactobacillaceae, DSV: Desulfovibrionaceae, D. desulfuricans: Desulfovibrio desulfuricans*, HFD：High-fat diet, WT: Wile type, *P. gingivalis: Porphyromonas gingivalis*, CD: Chow control diet, AA: Arachidonic acid, BFS: *Bacteroides fragilis* Supplementation.

Supplementary Table 4: This table presents the main characteristics of studies on the impact of GMMs on AS.

Title	Author	Time	Research object	Research aim	Main summary
Integrated metagenomics identifies a crucial role for trimethylamine-producing *Lachnoclostridium* in promoting atherosclerosis	Yuan-Yuan Cai et al	2022	Human ApoE−/− mice	This work aimed to identify TMA-producing genera in human intestinal microbiota.	This study discloses the significance and efficiency of the gut bacterium *L. saccharolyticum* in transforming choline to TMA and consequently promoting the development of atherosclerosis.
l-Carnitine in omnivorous diets induces an atherogenic gut microbial pathway in humans	Robert A. Koeth et al	2019	Human	This study explore the role of the gut microbiota in γBB generation in humans, and the impact of preceding dietary history and oral l-carnitine supplementation on both γBB generation and catabolism to TMA/TMAO in subjects. Finally, we also identify and characterize human fecal microbial community members that participate in the conversion of l-carnitine into TMA via the generation and catabolism of γBB.	In humans, dietary l-carnitine is converted into the atherosclerosis- and thrombosis-promoting metabolite TMAO via 2 sequential gut microbiota–dependent transformations: (a) initial rapid generation of the atherogenic intermediate γBB, followed by (b) transformation into TMA via low-abundance microbiota in omnivores, and to a markedly lower extent, in vegans/vegetarians. Gut microbiota γBB→TMA/TMAO transformation is induced by omnivorous dietary patterns and chronic l-carnitine exposure.
Plasma trimethylamine N-oxide (TMAO) levels predict future risk of coronary artery disease in apparently healthy individuals in the EPIC-Norfolk Prospective Population Study	W. H. Wilson Tang et al	2021	Human	The contributory role of the TMAO pathway in the development of coronary artery disease (CAD) in the general population, particularly amongst people who are apparently healthy, has not been established. This study investigate the prognostic value of baseline TMAO levels and the propensity for incident coronary artery disease (CAD) events in apparently healthy middle-aged people from the general population.	In apparently healthy participants of the community-based middle-aged EPICNorfolk population, elevated plasma levels of the gut microbe-dependent metabolite TMAO, and its nutrient precursor choline, predict incident risk for CVD development independent of traditional risk factors.
The Association between Trimethylamine N-Oxide and Its Predecessors Choline, L-Carnitine, and Betaine with Coronary Artery Disease and Artery Stenosis	Fei Guo et al	2020	Human	This study aim is to investigate the association of plasma TMAO and its predecessors in CAD and extent of artery lesion in different gender.	The associations of TMAO with CAD and severe artery stenosis were sex-related. TMAO alone was more powerful in determining CAD and artery stenosis in men than women, while a combination of TMAO, choline, L-carnitine, and betaine could be potential biomarkers for diagnosing CAD and artery stenosis in both men and women.

Choline and trimethylamine N-oxide supplementation in normal chow diet and western diet promotes the development of atherosclerosis in Apoe –/– mice through different mechanisms	Hong-Na Mu et al	2023	Apoe−/− mice	This study aim to investigate the effects of TMAO on atherosclerosis (AS) development and the underlying mechanisms.	This study found that adding choline or TMAO in WD for 12 weeks increased AS plaque formation through integrative effects involving inflammatory response, modulation of intestinal microbiota dysbiosis and microbiome-mediated functions on cholesterol and bile acid metabolism. Choline or TMAO supplementation in ND also promoted plaque progression in Apoe–/– mice through other mechanisms which need further study. In addition, TMAO may act differently in Apoe–/– mice when fed with ND or WD.
Choline Supplementation Does Not Promote Atherosclerosis in CETP-Expressing Male Apolipoprotein E Knockout Mice	Heidi L. Collins et al	2022	Apoe−/− mice	The objective of this study was to determine the effect of choline supplementation on atherosclerosis progression in Apoe−/− mice expressing human cholesterol ester transfer protein (hCETP) using the same diets as in previously reported studies.	In conclusion, this study failed to corroborate any association between TMAO and aortic atherosclerosis when feeding large doses of choline to non-CETP-expressing male Apoe−/− mice for 16 weeks. Enhancing reverse cholesterol transport in CETP-transfected male Apoe−/− mice also failed to show any difference in atherosclerosis despite a doseresponsive elevation of TMAO after 8 and 16 weeks. Taken together, these data from male Apoe−/− mice with or without expression of CETP do not support increased plasma TMAO as a causative agent of atherosclerosis.
Dietary Choline or Trimethylamine N-oxide Supplementation Does Not Influence Atherosclerosis Development in Ldlr−/− and Apoe−/− Male Mice	Paulina Aldana-Hernández et al	2020	Ldlr−/− mice<br>Apoe−/− mice	The aim of this study was to further investigate the relation between dietary choline and atherosclerosis in 2 atherogenic mouse models, the Ldlr−/− and Apoe−/− mice.	In the study, high intakes of dietary choline or TMAO supplementation did not influence atherosclerosis development in Ldlr−/− or Apoe−/− male mice.
Inhibition of Trimethylamine N-oxide attenuates neointimal formation through reduction of inflammasome and oxidative stress in a mouse model of carotid artery ligation	Chi-Yu Chen et al	2023	C57BL/6 mice<br>Human aortic smooth muscle cells	This study investigated the effects of high TMAO and high-choline diets on vascular remodeling in a partial carotid artery ligation model and examined the TMAO-lowering effects of DMB in vivo.	This study findings partly explain the mechanisms of TMAO-induced vascular inflammation and disturbed flow-induced atherosclerosis. Multiple mechanisms underlie the atherosclerotic changes during neointimal hyperplasia in mice fed high-choline and highTMAO diets, including increased inflammasome activation, increased ER stress, and the induction of ROS production and mitochondrial dysfunction.

Plasma Levels of TMAO can be Increased with ‘Healthy’ and ‘Unhealthy’ Diets and Do Not Correlate with the Extent of Atherosclerosis but with Plaque Instability	Yen Chin Koay et al	2021	C57BL/6J mice<br>Ldlr-/- mice<br>ApoE−/−mice<br>Human	The aims of this study were: (1) to evaluate the effect of ‘unhealthy’ (high choline) and ‘healthy’ (high fibre) diets on plasma TMAO levels and describe the different generative pathways in each case; (2) to examine the relationship between circulating TMAO levels and atherosclerosis in model systems and humans; (3) to characterize the relationship of TMAO and related metabolites to unstable plaque, the mechanism underlying cardiovascular events such as myocardial infarction.	This study data reveal distinct dietary – bacterial microbiome interactions leading to elevated plasma TMAO, in both ‘healthy’ and ‘unhealthy’ dietary contexts and provide insight into the conflicting reports of TMAO’s relationship to atherothrombosis. this study data suggest that there is no direct association of plasma TMAO and the extent of atherosclerosis. However, this study did demonstrate an association of TMAO plasma levels with atherosclerotic plaque instability. The latter is in accordance with TMAO being associated with an increased risk of cardiovascular events.
Gut-Flora-Dependent Metabolite Trimethylamine-N-Oxide Promotes Atherosclerosis-Associated Inflammation Responses by Indirect ROS Stimulation and Signaling Involving AMPK and SIRT1	Sa Zhou et al	2022	Vascular smooth muscle cells<br>Human umbilical vein endothelial cells<br>C57BL/6 mice	This study using human umbilical vein endothelial cells (HUVECs), aortic vascular smooth muscle cells (VSMCs) and a mouse model, they studied the molecular mechanisms on how TMAO could contribute to atherosclerosis development by exploring the possible underlying links between TMAO, ROS, inflammation factors and the signaling pathways involving SIRT1 and AMPK.	In summary, this study demonstrated that TMAO is associated with the development of atherosclerosis in vitro and in vivo. In vitro, we demonstrated that TMAO regulated ROS stimulation and AMPK and SIRT1 signaling to induce inflammation responses in VSMCs and HUVECs, which may contribute to promoting atherosclerosis (Figure 9). Moreover, the antioxidant NAC can inhibit the production of ROS and alleviate the inflammatory responses in VSMCs and HUVECs. In vivo, choline and TMAO promote the development of atherosclerosis. NAC and LP8198 also decelerate TMAO-induced atherosclerosis and affect the gut microbiota of C57BL/6J mice.
Fecal level of butyric acid, a microbiome- derived metabolite, is increased in patients with severe carotid atherosclerosis	Kristine Stø et al	2022	Human	This study aimed to investigate the association between fecal butyric acid, carotid atherosclerosis and risk factors for ischemic stroke.	In contrast to bulk of previous studies showing reduced abundance of butyric acid producing bacteria in patients with atherosclerosis and CVD, this study find that patients with severe carotid atherosclerosis and evidence of gut barrier damage have increased fecal level of butyric acid. This finding was supported by increased functional bacterial production of butyric acid. The study speculate that gut barrier damage could decrease intestinal absorption, and also influence the results. Further studies are needed to map out the role of butyric acid and other SCFAs in atherosclerosis and their potential future role when considering the gut as a therapeutic target to

					prevent symptomatic CVD.
Butyrate protects endothelial function through PPARδ/miR-181b signaling	Qinqin Tian et al	2021	ApoE-/-mice<br>C57BL/6 mice<br>PPARδ-/-mice<br>EC-specific PPARδ KO (Cdh5cre+; PPARδloxp/loxp) mice<br>EC-specific PPARδ WT (PPARδloxp/loxp) mice<br>Mouse brain microvascular endothelial cells	This study aims to investigate the potential roles of PPARδ and miR-181b signaling in mediating the vaso-protective effects of butyrate.	In summary, the study demonstrates that butyrate prevents endothelial dysfunction by reducing endothelial NOX2 expression and ROS generation via the PPARδ/miR-181b pathway.
Choline and butyrate beneficially modulate the gut microbiome without affecting atherosclerosis in APOE*3-Leiden.CETP mice	Cong Liu et al	2022	APOE*3-Leiden.CETP mice	The study was to investigate whether butyrate can alleviate choline-induced atherosclerosis.	In conclusion, this study demonstrate that in APOE*3-Leiden.CETP mice, a well-established model for human-like lipoprotein metabolism, both choline and butyrate beneficially modulate the gut microbiome and increase TMAO, however without affecting atherosclerosis.
Propionate attenuates atherosclerosis by immune-dependent regulation of intestinal cholesterol metabolism	Arash Haghikia et al	2022	Apoe-/- mice<br>Human	This study examined for the first time gut immunomodulatory effects of the microbiota-derived metabolite PA on intestinal cholesterol metabolism.	This study findings reveal a novel immune-mediated pathway linking the gut microbiota-derived metabolite PA with intestinal Npc1l1 expression and cholesterol homeostasis. The results highlight the gut immune system as a potential therapeutic target to control dyslipidaemia that may introduce a new avenue for prevention of ACVDs.

Butyrate suppresses atherosclerotic inflammation by regulating macrophages and polarization via GPR43/HDAC-miRNAs axis in ApoE−/− mice	Huiyan Ma et al	2023	ApoE−/− mice	This study examined the effects of orally administered butyrate on AS progression and associated mechanisms including Mψ polarization in inflammation, gut microbiota, and the role of miRNAs in HFD-induced atherosclerotic ApoE−/− mice.	This study provides anew evidence that butyrate could ameliorate the progression of inflammation in atherosclerosis through regulating macrophage polarization via GPR43-related and HDAC/PPAR-γ/NF-κB/NLRP3/miRNAs signal pathways.
Gut Microbially Produced Indole-3-Propionic Acid Inhibits Atherosclerosis by Promoting Reverse Cholesterol Transport and Its Deficiency Is Causally Related to Atherosclerotic Cardiovascular Disease	Hongliang Xue et al	2022	Huamn<br>C57BL/6 mice<br>ApoE−/− mice<br>Abca1-Flox mice<br>Lyz2-Cre transgenic mice<br>Mouse peritoneal macrophages<br>Human THP-1 monocytes<br>Aortic endothelial cells<br>Aortic smooth muscle cells<br>HEK293 cells	This study performed an integrated microbiomemetabolome analysis of fecal and serum samples from subjects with CAD, coupled with mechanistic investigations, to reveal that the key gut microbiota-derived tryptophan metabolite IPA is both clinically and mechanistically linked to ASCAD and is shown to promote macrophage RCT through a newly identified miR-142-5p/ABCA1 signaling pathway.	In conclusion, this study identified IPA, as a key microbially produced tryptophan metabolite, is negatively associated with ASCVD risk. IPA promotes macrophage RCT through a previously undescribed miR-142-5p/ABCA1 pathway in ApoE−/− mice, thus alleviating the development of atherosclerotic plaque. Furthermore, the study demonstrated that IPA and miR-142-5p are promising candidates for atherosclerosis prevention and treatment.
Tryptophan metabolism, gut microbiota, and carotid artery plaque in women with and without HIV infection	Kai Luo et al	2022	Human	This study aimed to investigate the interrelationship between tryptophan metabolites, gut microbiota, and carotid artery plaque among women living with and without HIV in the WIHS.	In a cohort of women living with and without HIV infection, plasma IPA levels and related gut bacteria were inversely associated with carotid artery plaque, suggesting a potential beneficial role of IPA and its gut bacterial producers in atherosclerosis and CVD.

Gut microbiome-derived glycine lipids are diet-dependent modulators of hepatic injury and atherosclerosis	Courtney L. Millar et al	2022	Ldlr−/− mice<br>Apoe−/− mice<br>RAW264.7 macrophages	This study sought to investigate the bioactivities of bacterial glycine lipids in mouse models of atherosclerosis and characterize the glycine lipid content in the feces of mice fed different diets.	In conclusion, the study shows that *Bacteroidetes*-derived fecal glycine lipids are significantly reduced with Western-type HFD, and that chronic intraperitoneal exposure to the microbiota-derived serineglycine lipodipeptide, L654, may prevent atherosclerosis progression and liver injury via hypolipidemic and antiinflammatory effects. The lower MHC class II gene expression seen in L654-treated mice suggests that liver tolerance was partially maintained during the inflammatory insult of Western-type HFD feeding. A breakdown of tolerance, because of a change in the gut microbiome or other aspects of HFD or obesity, may induce an inappropriate immune response, resulting in acute and chronic inflammatory liver diseases. Therefore, conditions where gut microbiome-derived glycine lipids are lost, such as HFD-induced obesity, may exacerbate development of atherosclerosis and liver injury, while correction of such depletion may attenuate these disorders.
Glycoursodeoxycholic Acid Ameliorates Atherosclerosis and Alters Gut Microbiota in Apolipoprotein E–Deficient Mice	Kan Huang et al	2021	Human THP-1 monocytes<br>ApoE-/-mice	This study aimed to investigate the role of GUDCA in the development of atherosclerosis and its potential mechanisms.	This study for the first time indicates that GUDCA attenuates the development of atherosclerosis, probably attributable to the inhibition of foam cell formation, maintenance of cholesterol homeostasis, and modulation of gut microbiota.
A novel candidate for prevention and treatment of atherosclerosis: Urolithin B decreases lipid plaque deposition in apoE-/- mice and increases early stages of reverse cholesterol transport in ox-LDL treated macrophages cells	WenhuaZhao et al	2019	THP-1 cells<br>Murine macrophage-like cell line J774<br>ApoE-/- mice	This study investigated effects of urolithin B on lipid plaque deposition, detected effects of urolithin B and its phase II conjugated metabolite on cholesterol uptake, esterification and release in ox-LDL induced foam cells including J774 and THP-1 cells.	In summary, this study showed that urolithin B can decrease the lipid plaque deposition. Urolithin B and urolithin B sulphate are not only modulating expression of SR-B1 and ABCA1 involved in reverse cholesterol, but also are able to increase cholesterol efflux from cholesterol laden macrophages to HDL particle which is the initial step within the reverse cholesterol transport. This study suggest that beneficial effects of urolithin B, and its derivatives, in animal models could promote the development of new drugs for prevention and treatment of atherosclerosis in humans.

Urolithin A promotes atherosclerotic plaque stability by limiting inflammation and hypercholesteremia in Apolipoprotein E–deficient mice	Meng-yun Xu et al	2024	ApoE−/− mice Human umbilical vein endothelial cells	This study investigated the pharmacological actions of UroA on endothelial inflammation and lipid metabolism by assessing atherosclerotic lesion area, plaque composition, and expression of genes involved in cholesterol metabolism in ApoE-/mice.	This study provides supporting evidence for the anti-atherosclerotic effect of UroA and reveals the underlying mechanism at the cellular and molecular levels. UroA exerts anti-atherosclerotic effects by inhibiting vascular inflammation and lowering blood lipid levels.

AS: atherosclerosis, ApoE-/- mice: Apolipoprotein E knockout mice, Ldlr−/− mice: Low-density lipoprotein receptor knockout mice, TMA: Trimethylamine, γBB: γ-Butyrobetaine, TMAO: Trimethylamine N-oxide,AMPK: AMP-activated protein kinase, NAC: N-Acetylcysteine, ROS: Reactive Oxygen Species, CVD: Cardiovascular Disease, NOX2: NADPH Oxidase 2, PPARδ: Peroxisome Proliferator-Activated Receptorδ, miR-181b: MicroRNA-181b, NPC1L1: Niemann-Pick C1-Like 1 Protein, ACVDs: Atherosclerotic Cardiovascular Diseases HDAC miRNAs: Histone Deacetylase-related microRNAs, PPAR-γ miRNAs: Peroxisome Proliferator-Activated Receptor gamma-related microRNAs, NF-κB miRNAs: Nuclear Factor kappa B-related microRNAs, NLRP3 miRNAs: NOD-like Receptor Protein 3-related microRNAs. RCT: Reverse Cholesterol Transport, miR-142-5: MicroRNA-142-5p, ABCA1:ATP-Binding Cassette Transporter A1, ASCAD: atherosclerotic cardiovascular disease, HIV: Human Immunodeficiency Virus, ox-LDL: Oxidized Low-Density Lipoprotein, SIRT1: sirtuin1, ND: normal chow diet, WD: western diet, hCETP: human cholesterol ester transfer protein, IPA: indole-3-propionic acid,CAD: Coronary Artery Disease, HFD：High-fat diet, PA: propionic acid, WIHS: Women's Interagency HIV Study, GUDCA: Glycoursodeoxycholic Acid, UroA: Urolithin A,DMB: 3,3-Dimethyl-1-butanol.

Supplementary Table 5: This table presents the characteristics of studies on AS-related factors intervention mediated by GM.

Title	Author	Time	Research object	Research aim	Main summary
A consortium of three-bacteria isolated from human feces inhibits formation of atherosclerotic deposits and lowers lipid levels in a mouse model	Zhuye Jie et al	2023	Apoe-/-mice<br>Human	From an established collection of bacteria isolated from healthy Chinese individuals, the study selected *B. cellulosilyticus, R. intestinalis*,and *Faecalibacterium longum*, a bacterium related to *F. prausnitzii,* and tested the effects of the sebacteria in an Apoe - / - atherosclerosis mouse model.	In conclusion, this study demonstrates a protective effect of administration of *B. cellulosilyticus, F. longum,* and *R. intestinalis* to Apoe -/- mice suggesting a potential for use of these bacteria to prevent/ameliorate ACVD.
Isolation and characterization of a novel choline degrading Citrobacter amalonaticus strain from the human gut	Jyoti Kashyap et al	2022	Human	This study hypothesized that alternative pathways for choline degradation occur within gut microbes and that certain gut microbiota can anaerobically respire or ferment QAs, such as choline.	This study have isolated, from a choline-supplemented enrichment of a human fecal sample, a strain of Citrobacter amalonaticus, that we have designated CJ25.<br>Strain CJ25 is a unique bacterium that degrades choline without the production of the proatherogenic metabolite TMA.
Metagenomic analysis of the gut microbiome in atherosclerosis patients identify cross-cohort microbial signatures and potential therapeutic target	Sheng Liu et al	2020	Human	This study reanalyzed the data in these two cohorts using MetaPhlAn2 and HUMAnN2, to provide a comprehensive species-level overview of the gut microbial composition and function. This study also identified microbial strains using the tool PanPhlAn, and predicted metabolomic profiling of microbial communities using the latest computational approach called MelonnPan.	This study results indicate intestinal bacteria such as *B xylanisolvens, E eligens*, and *R inulinivorans* could be promising probiotics and potential therapeutic target for atherosclerosis.
Faecalibacterium prausnitzii as a potential Antiatherosclerotic microbe	Hai- Tao Yang et al	2024	Human<br>Apoe-/- mice<br>HCT-116 Human colon carcinoma cell line	This study aimed to identify key biomarkers using metagenomics and untargeted metabolomics and verify their associations with atherosclerosis.	Sequencing of the samples revealed a previously unknown link between specific gut microbiota and atherosclerosis. Treatment with *F. prausnitzii* may help prevent CAD by inhibiting atherosclerosis.
Bifidobacterium animalis subsp. lactis F1−7 Alleviates Lipid Accumulation in Atherosclerotic Mice via Modulating Bile Acid Metabolites to Downregulate Intestinal FXR	XiLiang et al	2024	ApoE−/− mice<br>C57BL/6 mice	This study explored themechanism of *Bifidobacterium animalis subsp. lactis F1−7 (Bif. animalis F1−7)*, improving atherosclerosis by regulating the bile acid metabolism and intestinal microbiota in	This study hypothesized that changes in the gutmicrobiota ofatherosclerotic mice following *Bif. animalis F1−7* intervention could affect bileacid metabolism, thereby improving atherosclerotic lipid metabolism.

				the ApoE −/− mice.	
Enterobacter aerogenes ZDY01 inhibits cholineinduced atherosclerosis through CDCA-FXR-FGF15 axis	Jinghui Tang et al	2021	ApoE−/− mice	This study evaluated the anti-atherosclerotic effects of *E. aerogenes ZDY01* in choline-fed ApoE−/− mice and investigated how *E. aerogenes ZDY01* improved choline-induced atherosclerosis by decreasing cecal TMA levels and promoting RCT.	Taken together, reduced levels of serum TMAO and cholesterol by *E. aerogenes ZDY01* ameliorated choline-induced atherosclerosis in ApoE−/− mice.
Anti-atherosclerotic effects of *Lactobacillus plantarum ATCC 14917* in ApoE−/− mice through modulation of proinflammatory cytokines and oxidative stress	Adil Hassan et al	2020	ApoE−/− mice	The study aimed to investigate whether *Lactobacillus plantarum ATCC 14917* could attenuate the formation of atherosclerotic lesion in ApoE−/− mice by modulating the proinflammatory cytokines, blocking the translocation of P65 protein NF-κB of oxidative stress and improving the composition of gut microbiota in model mice.	In conclusion, the atheroprotective effect of *L. plantarum ATCC 14917* was determined by modulating proinflammatory cytokines and gut microbial diversity and inhibiting oxidative stress. *L. plantarum ATCC 14917* also contributed to the alternation of gut microbiota.
Lactic acid bacteria prevent both periodontitis and atherosclerosis exacerbated by periodontitis in spontaneously hyperlipidemic mice	Ryoki Kobayashi et al	2021	C. KOR-Apoeshl mice	This study evaluated the potential of LAB to change the intestinal flora changes induced by periodontopathic bacteria and to prevent/slow down the development of atherosclerosis.	These results suggest that oral Lg treatment is effective in preventing periodontitis and atherosclerosis.
Krill Oil Combined with *Bifidobacterium animalis subsp. lactis F1-7* Alleviates the Atherosclerosis of ApoE−/− Mice	Xi Liang et al	2021	AopE−/− mice	The purpose of this study was to explore the improvement effect of KO combined with probiotics on atherosclerosis.	This study proved that *Bif. animalis F1-7* might play a synergistic effect in the improvement of inflammation by KO to the alleviation of atherosclerosis.
Metabolite acetyl-L-carnitine participates in *Bifidobacterium animalis F1-7* to ameliorate atherosclerotic inflammation by downregulating theTLR4/NF-κB pathway	Xi Liang et al	2024	C57BL/6J mice ApoE-/- mice	This study aimed to explore the effect of *Bifidobacterium animalis F1-7* on the improvement of atherosclerotic inflammation.	This study results revealed that *B. animalis F1-7* upregulated the metabolite ALC to downregulate the inflammatory responses, leading to the reduction of plaque accumulation of atherosclerosis.

Reduction of intestinal trimethylamine by probiotics ameliorated lipid metabolic disorders associated with atherosclerosis	Xi Liang et al	2020	C57BL/6J mice	The purpose of this study was to explore the effect of TMA-degrading probiotic agents on TMAO and the related lipid metabolism in mice.	*Bif. animalis subsp. lactis F1-3-2* could be colonized in the cecum, and might directly degrade TMA or change the structure of intestinal flora. The strain had an effect on TMA and TMAO levels in vivo by decreasing cecum TMA. The strain was demonstrated to participate in the TMA/TMAO regulation, improve the lipid metabolism, and alleviate atherosclerosis caused by TMAO. However, FMO3 had not changed in this process, and needs further study.
Lactobacillus rhamnosus GG protects against atherosclerosis by improving ketone body synthesis	Taiyu Zhai et al	2022	ApoE-/-mice	This study clarified the antiatherosclerotic effect of strain GG and analyzed its effect on intestinal microbial composition and peripheral blood metabolites in mice fed a high-fat diet by 16S rRNA sequencing and nontargeted metabolomics.	In conclusion, this study provides new evidence that strain GG slows the progression of AS, which may be associated with its improvement of the gut microbiome and peripheral blood metabolome, its ability to increase the abundance of beneficial bacteria, and its participation in unsaturated fatty acid and ketone body synthesis and degradation.
Chitin oligosaccharides alleviate atherosclerosis progress in ApoE-/mice by regulating lipid metabolism and inhibiting inflammation	Hongmin Zhen et al	2022	ApoE-/- mice	The study aims to evaluate whether Chitin oligosaccharides (NACOS) treatment can prevent atherosclerosis induced by a HFD in ApoE-/- mice.	NACOS supplementation ameliorated atherosclerosis induced by HFD in ApoE-/- mice by altering cholesterol metabolism and reducing inflammation. NACOS facilitated the anti-atherosclerosis intervention through the decrease of hepatic HMGCR to reduce cholesterol synthesis, activation of genes involved in RCT to enhance cholesterol efflux and excretion, and reduction of intestinal NPC1L1 to lower cholesterol absorption.
Dissecting the impact of dietary fiber type on atherosclerosis in mice colonized with different gut microbial communities	Evan R. Hutchison et al	2023	Germ-free ApoE−/− mice with fecal samples from three human donors (DonA, DonB, and DonC)	This study tested whether the effects of dietary fiber on atherosclerosis are influenced by the gut microbiome.	This study results suggest that atheroprotection in response to FF is not universal and is influenced by the gut microbiome.

Neoagarotetraose Alleviates Atherosclerosis via Modulating Cholesterol and Bile Acid Metabolism in ApoE−/− Mice	Junyi Li et al	2024	ApoE−/− mice C57BL/6J mice	The study was conducted to investigate the protective effects and potential mechanisms of neoagarotetraose on high-fat, HFHCD-induced atherosclerosis in ApoE−/− mice.	In conclusion, dietary supplementation with NAT (1200 mg/kg bw/d) effectively suppressed AS development in ApoE−/− mice fed a HFHCD, resulting in a decrease in the aortic root lesion area by 44.5%. The anti-atherosclerotic effect of NAT is mediated through the interaction between gut microbiota and bile acid, especially the decreased intestinal FXR expression of 35.8%. These findings demonstrate the potential application of NAT as a novel dietary supplement to prevent AS and provide a novel approach for addressing this cardiovascular disease.
Manno-oligosaccharides from Cassia Seed Gum Attenuate Atherosclerosis through Inflammation Modulation and Intestinal Barrier Integrity Improvement in ApoE−/− Mice	Junyi Li et al	2024	ApoE−/− mice C57BL/6J mice	This study investigates the anti-atherosclerotic effects of Manno-oligosaccharides from cassia seed gum (CMOS) on ApoE−/− mice.	CMOS inhibit inflammation, alter intestinal barrier integrity, and regulate gut microbiota to attenuate AS in ApoE−/− mice.
Potential effects of sialic acid and 3′-Sialyllactose on intestinal health and anti-cardiovascular disease in mice fed with a high-fat diet	Meizhen Zhu et al	2024	C57BL/6J mice	This study investigated the beneficial effects of SA and 3′-SL on the intestinal and cardiovascular health of mice fed with a high-fatdiet.	SA and 3'-SL have potentialprotective effects on intestinal health and the prevention of CVD. They regulateintestinal genes, which are related to intestinal mucin synthesis, digestion and absorption, immunity and circadian rhythm, change in intestinal flora and metabolites to protect the intestinal barrier and reduce inflammation, and blood sugar and blood lipids. In addition, CVD is further prevented by increasing the sialylation of LDL.
Protective Activities of Polysaccharides from Cipangopaludina chinensis Against High-Fat Diet Induced Atherosclerosis via Regulating Gut Microbiota in ApoE-deficient Mice	Qingping Xiong et al	2019	ApoE-/- mice	This study aimed to determine the preventive effects of CCPS on HFD induced AS in mice and its underlying mechanism focusing on gut microbiota profile modulation.	The above results demonstrated that CCPS supplementation could modulate the gut microbiota composition in HFD fed ApoE-/- mice to slow down the development of AS.
The Antioxidant Dendrobium officinale Polysaccharide Modulates Host Metabolism and Gut Microbiota to Alleviate High-Fat Diet-Induced Atherosclerosis in ApoE−/− Mice	Jingyi Qi et al	2024	ApoE−/− mice	This study aims to investigate the inhibitory effect and the potential mechanism of DOP on high-fat diet-induced atherosclerosis in ApoE−/− mice.	This study suggests that DOP has the potential to be developed as a food prebiotic for the treatment of atherosclerosis in the future.

The amelioration of a purified Pleurotus abieticola polysaccharide on atherosclerosis in ApoE−/− mice	Lei Xing et al	2024	Murine macrophage cells RAW 264.7<br>C57BL/6 mice<br>ApoE−/− mice	The amelioration of the atherosclerotic effects of PAPS2 was investigated in HFD-fed ApoE−/− mice.	PAPS2 ameliorated atherosclerosis by reducing oxidative stress and inflammation, which may be associated with the regulation of the intestinal microflora. PAPS2 is a potential auxiliary anti-atherosclerotic agent that targets lipid metabolism and inflammatory responses.
The effect and mechanism of inulin on atherosclerosis is mediated by the characteristic intestinal flora and metabolites	Zhenwei Li et al	2024	ApoE−/−mice	Utilizing a mouse model induced by a high-fat diet, this study aimed to explore whether the characteristic intestinal flora and its metabolites mediate the effects of inulin intervention on atherosclerosis and to clarify the specific mechanism.	Inulin can inhibit the formation of atherosclerotic plaques, which may be related to the changes in lipid metabolism, the composition of the intestinal microbial community and its metabolites, and the inhibition of the expression of related inflammatory factors.
Whole milk consumption is associated with lower risk of coronary artery calcification progression: evidences from the Multi- Ethnic Study of Atherosclerosis	Sounak Ghosh et al	2021	Human	This study attempted to investigate the relationship betthis studyen whole milk consumption and CAC progression, and the potential effect of SCFA in it.	Self-reported whole milk consumption was inversely associated with CAC progression in community-dwelling participants, especially in those at relatively low cardiovascular risks. The beneficial effect was partially mediated by SCFA. Therefore, whole milk can be incorporated into part of a cardio-protective diet. Regarding this, future studies may target.
The Effects of Moderate Alcohol Consumption on Circulating Metabolites and Gut Microbiota in Patients With Coronary Artery Disease	Xinyue Zhao et al	2021	Human	The association of moderate alcohol consumption with serum metabolites and gut microbiome and its impact on CAD is not fully investigated.	In general, this study provided a novel insight into the effect of moderate alcohol consumption on cardiovascular health by affecting gut microbiota and serum metabolome. The impact on metabolites and microbiota in patients with CAD with moderate drinking seems to be separated from those in patients with CAD with heavy drinking or non-drinking. Drinking moderately may have more positive effects on the metabolic profiles and commensal flora of patients with CAD, which may explain how moderate drinking affects cardiovascular health.

Cow's milk polar lipids reduce atherogenic lipoprotein cholesterol, modulate gut microbiota and attenuate atherosclerosis development in LDL-receptor knockout mice fed a Western-type diet	Courtney L. Millar et al	2020	LDLr−/− mice	This study hypothesized that dietary milk PLs would have cholesterollowering and anti-inflammatory properties, ultimately preventing atherosclerosis development involved in heart disease. To test this hypothesis, this study investigated whether feeding milk PLs (at 0%, 1% and 2% of HFD by weight) for 14 weeks affected (a) blood lipids, (b) inflammation, (c) gut microbiota composition and (d) development of atherosclerosis in LDLr−/− mice.	In conclusion, supplementing AMF-rich diet-fed LDLr−/− mice with 2% (w/w) milk PL lowered atherogenic lipoprotein cholesterol, modulated gut microbiota and modestly decreased inflammatory markers in the serum, liver, adipose and aorta. These factors likely contributed to the strong attenuation of atherosclerosis development in 2% milk PL-fed mice. Thus, milk PL content may be important to consider when choosing dairy products as food. Further research in humans is warranted to confirm these preclinical findings.
Dietary Fruit and Vegetable Supplementation Suppresses Diet-Induced Atherosclerosis in LDL Receptor Knockout Mice	Weimin Guo et al	2021	LDLR-KO mice	This study aimed to determine whether there is a causal relation between consuming high levels of F&V and prevention of atherosclerosis, the hallmark of CVD pathogenesis. Furthermore, the underlying mechanisms were determined.	This study results indicate that consuming a large quantity and variety of F&Vs causally attenuates diet-induced atherosclerosis and hepatic steatosis in mice. These effects of F&Vs are associated with, and may be mediated through, improved atherogenic dyslipidemia, alleviated gut dysbiosis, and suppressed inflammation.
Red yeast rice ameliorates high-fat diet-induced atherosclerosis in Apoe−/− mice in association with improved inflammation and altered gut microbiota composition	Yanhan Dong et al	2019	Apoe−/− mice	This study aimed to evaluate the effects of RYR supplementation in a high-fat diet in terms of several atherosclerosis parameters and the contribution of intestinal gut microbiota in Apoe−/− mice.	In summary, the study adds to the accumulating evidence that red yeast rice attenuates high-fat diet-induced atherosclerosis, accompanied with decreased plaque size, plasma lipid levels, inflammation, and improvement of the gut barrier and gut microbiota structure. In addition, the MAPK pathway was elucidated to be involved in the anti-atherosclerotic effect of RYR. , the study results further support the hypothesis that the underlying mechanisms of the anti-atherosclerotic effect of RYR are closely linked with the structural changes of gut microbiota.
Dosage of Dual-Protein Nutrition Differentially Impacts the Formation of Atherosclerosis in ApoE−/− Mice	Yingchun Huang et al	2022	ApoE−/− mice	This study aimed to verify the effect of DP on AS and explore the optimal DP intake to improve AS.	In summary, the study demonstrated that long-term supplementation of DP at a dose of 0.67 g/kg/day can reduce high-fat-diet-induced atherosclerosis in ApoE−/− mice, which was accompanied by improvements in vascular plaque, lipid metabolism, inflammatory response,

					intestinal barrier function, and gut microbiota structure.
Effects of Whole Brown Bean and Its Isolated Fiber Fraction on Plasma Lipid Profile, Atherosclerosis, Gut Microbiota, and Microbiota-Dependent Metabolites in Apoe−/− Mice	Jiyun Liu et al	2022	Apoe−/− mice	The aim of this study was therefore to evaluate and compare the effects of whole brown beans and the isolated dietary fiber fraction on lipid profile, atherosclerotic plaque amount, gut microbiota, and microbiota-dependent metabolites in Apoe−/− mice fed a HF diet.	In summary, both the Wbean and Bfiber diets resulted in a tendency for lower atherosclerotic plaque amount, but did not positively affect body weight and plasma lipid profile compared with the HF diet. The Wbean diet led to a higher alpha diversity of gut microbiota compared with the HF control. Both bean diets resulted in higher relative abundance of *Actinobacteria* and *Bacteroidetes*, and lower F/B ratio. At the genus level, both bean diets showed similar effects on the stimulation of the growth of bacterial genera, such as *unclassified S24-7, Prevotella, Bifidobacterium*, and *unclassified Clostridiales*, and led to lower relative abundance of *Lactobacillus*. The Bfiber fraction also resulted in lower abundance of *Oscillospira*. A higher formation of all cecal SCFAs, a higher proportion of cecal propionic acid, and a lower proportion of cecal acetic acid were observed in mice on both bean diets. The bean diets also resulted in higher plasma TMAO concentrations compared with the HF diet, while the Bfiber diet led to lower plasma creatinine concentrations and the Wbean diet showed a lowering tendency.

Fish Oil Is More Potent than Flaxseed Oil in Modulating Gut Microbiota and Reducing Trimethylamine- N- oxide-Exacerbated Atherogenesis	Zouyan He et al	2019	ApoE−/− mice	The study was conducted (i) to investigate whether flaxseed oil or fish oil was able to suppress the TMAO exacerbated atherogenesis and (ii) to compare the relative potency of fish oil with that of flaxseed oil against TMAO aggravated inflammation, cholesterol dysregulation, and modulation in gut microbiota in ApoE−/− mice fed a WD.	In conclusion, the study clearly demonstrated that dietary TMAO accelerated atherogenesis, disturbed the cholesterol metabolism, and altered the gut microbial composition in ApoE−/− mice. Fish oil exerted more potent cardioprotective effects than flaxseed oil on TMAO-exacerbated formation of atherosclerotic plaque by lowering plasma cholesterol, inhibiting inflammation, and modulating gut microbial composition in a way to favorably promote the production of SCFAs and inhibit the microbial generation of LPS.
Camellia oil (Camellia oleifera Abel.) treatment improves highfat diet-induced atherosclerosis in apolipoprotein E (ApoE)−/− mice	Tianyang HUANG et al	2023	C57BL/6J mice ApoE−/− mice	This study investigated the beneficial effects of camellia oil in an ApoE−/− mouse model of atherosclerosis and explored its potential underlying mechanisms.	In conclusion, this is the first study to demonstrate that camellia oil significantly inhibited the formation of atherosclerotic plaques in ApoE−/− mice. Mechanistically, this preventive effect of camellia oil was probably due to the reduced levels of serum total cholesterol, triacylglycerol, and low-density lipoprotein cholesterol and the improved levels of serum high-density lipoprotein cholesterol. In addition, the decreased levels of TNF-α and alteration of the gut microbiota composition induced by camellia oil might contribute to its preventive effect against atherosclerosis in mice.
Natto consumption suppresses atherosclerotic plaque progression in LDL receptor- deficient mice transplanted with iRFP- expressing hematopoietic cells	Takeshi Kawamata et al	2023	A strain of iRFP-expressing bone marrow transplanted mice (iRFP → LDLR−/−),iRFP → LDLR-/- mice	The study evaluated the impact of natto consumption on atherosclerotic progression using an in vivo murine imaging model and strains of natto with varying vitamin K2 levels developed originally to accommodate patients on anticoagulation therapy.	The study results suggest that natto may possess antiatherosclerotic activities through multiple pathways, including the modulation of inflammatory cytokines produced by macrophages and the anti-inflammatory action of vitamin K. Further gene expression analysis and cytokine secretion measurements have elucidated that natto suppresses inflammatory activation of macrophages via inhibition of the NF-kB signaling pathway and the NLRP3 inflammasome. Future studies should focus on the evaluation of macrophage activation markers in

					atherosclerotic lesions, identification and isolation of functional components in natto, and detailed elucidation of their mechanisms of action.
Effects of Thermally-Oxidized Frying Oils (Corn Oil and Lard) on Gut Microbiota in Hamsters	Erika Kwek et al	2022	Golden Syrian hamsters	The study was designed to investigate the effects of thermally-oxidized corn oil and lard on gut microbiota in relation to atherosclerosis, inflammatory cytokines, and plasma lipids.	The study was concluded that frying oil could adversely modulate the gut microbiota and exacerbate the atherosclerosis at least in a hypercholesterolemia hamster model.
Dietary α-Linolenic Acid-Rich Flaxseed Oil Ameliorates High-Fat Diet-Induced Atherosclerosis via Gut Microbiota-Inflammation-Artery Axis in ApoE−/− Mice	Yiwei Li et al	2022	ApoE−/− mice	Thus, this study was aimed to investigate the effectiveness of the dietary alpha-linolenic acid-rich FO on the occurrence and the development of AS in ApoE−/− mice, with or without gut microbiota, which may potentially contribute to the further understanding of complicated mechanisms among AS, gut microbiota, and inflammation.	This study highlighted that the dietary ALA-rich FO mainly ameliorated the HFD-induced AS via gut microbiotainflammation-artery axis in ApoE−/− mice, which potentially served as the inexpensive interventions for the prevention and treatment of the disease.
Oral Administration of Branched-Chain Amino Acids Attenuates Atherosclerosis by Inhibiting the Inflammatory Response and Regulating the Gut Microbiota in ApoE-Deficient Mice	Ziyun Li et al	2022	ApoE−/− mice	The purpose of this study was to determine the effects of dietary BCAA supplementation on AS in ApoE−/− mice and the underlying mechanisms, with a focus on gut microbiota remodeling and the inflammatory response.	In conclusion, this study demonstrates that dietary supplementation with BCAAs may not only alleviate AS by suppressing the inflammatory response, but may also regulate bile acid excretion by altering intestinal flora in ApoE-deficient mice. This research supported the beneficial effects of BCAAs on inflammation and elucidated their potential mechanisms, providing different perspectives on the molecular mechanisms of BCAA supplementation to alleviate AS.

Medium-, long- and medium-chain-type structured lipids ameliorate high-fat diet-induced atherosclerosis by regulating inflammation, adipogenesis, and gut microbiota in ApoE−/− mice	Chonghui Yue et al	2020	C57BL/6J mice ApoE−/− mice	The aim of this study was to investigate the effects of MLM structured lipids structured lipid supplementation on the inflammation, adipogenesis and gut microbiota in high-fat diet-induced atherosclerosis in ApoE−/− mice.	This study results indicated that MLM structured lipids could improve the development of atherosclerosis and reduce the accumulation of lipids in high-fat diet-fed ApoE−/− mice by regulating the composition and diversity of the gut microbiota.
Dietary Egg Sphingomyelin Prevents Aortic Root Plaque Accumulation in Apolipoprotein-E Knockout Mice	Courtney L. Millar et al	2019	ApoE−/− mice	The aim of this study was to determine if incorporating 0.1% (w/w) ESM (equivalent to ~750 mg/day in humans) into a high-fat (45% kcal), cholesterol-enriched diet (HFD) could prevent atheroprogression in apoE−/− mice .	There was also a modulation of the gut microbiota with ESM supplementation. ESM may have the potential to prevent atherosclerosis, however further research in the clinical setting is warranted.
Anti-Atherosclerotic Properties of Wild Rice in Low-Density Lipoprotein Receptor Knockout Mice: The Gut Microbiome, Cytokines, and Metabolomics Study	Mohammed H. Moghadasian et al	2019	LDL-r-KO mice	This study aimed to investigate the impact of wild rice on bacterial species abundance and diversity from 16S rDNA data analysis collected from mouse feces and monitor the metabolic products from the feces and plasma of LDL-r-KO mice.	In conclusion, this study hereby report that the long term consumption of wild rice at 60% (w/w) in LDL-r-KO mice is associated with the prevention of atherosclerosis. This effect was accompanied by significant alterations in the fecal bacterial population and diversity, as well as significant changes in several inflammatory and metabolic biomarkers. Of particular interest was an increase in the plasma glucose levels in the wild rice fed mice; currently, this study have no explanation for this finding. Other findings that can support anti-atherogenic properties of wild rice are increases in the plasma levels of anti-inflammatory marker IL-10 and EPO. Altogether, this study provides preliminary evidence in support of additional studies on this animal model and others to improve our understanding of how gut bacterial species, plasma inflammatory markers, and metabolic biomarkers may prevent atherosclerosis. Furthermore, a dose-response study can help to establish whether lower doses of wild rice can result in similar findings in this animal model.

Oat fiber supplementation alleviates intestinal inflammation and ameliorates intestinal mucosal barrier via acting on gut microbiota-derived metabolites in LDLR / mice	Hui Gao et al	2022	LDLR-/- mice		The aim of this study was to evaluate how oat fiber acted on gut microbiota-derived metabolites, inhibited intestinal inflammation, and protected the intestinal mucosal barrier.	The study revealed that oat fiber feeding effectively attenuated the development of atherosclerosis, at least partly via affecting gut microbiota-derived metabolites, inhibiting the intestinal inflammatory response, and maintaining the integrity of the intestinal mucosal barrier.
Effects of Different Carbohydrate Content Diet on Gut Microbiota and Aortic Calcification in Diabetic Mice	Xinyi Shen et al	2024	ApoE−/− mice		This study aimed to investigate the impact of carbohydrates on gut microbiota and aortic calcification in diabetic ApoE−/− mice.	Ketogenic diet could delay the onset of aortic atherosclerosis, aortic calcification and improve intestinal barrier function in diabetic ApoE−/− mice.
Whole-Grain Highland Barley Attenuates Atherosclerosis Associated with NLRP3 Inflammasome Pathway and Gut Microbiota in ApoE−/− Mice	Tong Wu et al	2023	ApoE−/− mice		This study aimed to explore whether highland barley supplementation can prevent atherosclerosis progression and improve gut microbiota disorder in apolipoprotein E knockout (ApoE−/−) mice.	This study aimed to examine the potential of highland barley in mitigating atherosclerosis by investigating its effects on the NLRP3 inflammasome pathway and gut microbiota. The results of our study provide support for this hypothesis, as the study found that WHB exhibits stronger biological activities compared to refined highland barley RHB. One of the key factors contributing to the enhanced biological activities of WHB is its minimal processing, which allows it to retain a substantial amount of bioactive compounds. These compounds play a critical role in stimulating the growth and regulating the metabolic effects of the microbiota in the large intestine. As a result, WHB demonstrates an antiinflammatory capacity, which is particularly relevant to the development of atherosclerosis.

Cabernet sauvignon dry red wine ameliorates atherosclerosis in mice by regulating inflammation and endothelial function, activating AMPK phosphorylation, and modulating gut microbiota	Xinlong Cheng et al	2023	ApoE -/- mice	This study examined the anti-atherosclerotic effect of CSDRW in a mouse model of AS using metabolomic profiling and molecular techniques.	This study investigated the anti-atherosclerotic effect and mechanism of CSDRW in AS mice. Oral administration of CSDRW suppressed AS development by mitigating inflammation, improving hepatic lipid metabolism, alleviating oxidative stress, regulating the PPAR γLXR-α-ABCA1 pathway in the liver, and promoting the proliferation of some key gut microbes, including *Akkermansia, Christensenellaceae_R-7*, and *Eubacterium_fissicatena*. Overall, these results suggest that moderate consumption of CSDRW could benefit cardiovascular health and prevent AS. However, the synergism between polyphenols and ethanol requires further investigation in laboratory and clinical studies of AS.
Gut Microbiota Functional Dysbiosis Relates to Individual Diet in Subclinical Carotid Atherosclerosis	Andrea Baragetti et al	2021	Human	This study here address the relation between functional metagenomic signatures and individual exposure to diet during subclinical manifestation of CVD.	These findings might contribute to hypothesize future strategies of personalized dietary intervention for primary CVD prevention setting.
The gut microbiome in subclinical atherosclerosis: a population-based multiphenotype analysis	Sibo Zhu et al	2021	Human	The aim of this study was to better understand the role of the gut microbiota in vascular physiology in a subclinical elderly population, and to investigate how lifestyle affects the composition of host gut microbiota to further impact the pathogenesis of vascular diseases.	This study revealed a Chinese population–wide phenotype–metagenomic association network and a mediation effect of gut microbiota on carotid artery atherosclerosis, hinting at potential therapeutic and preventive uses for microbiota in vascular diseases.
Dietary titanium dioxide particles (E171) promote diet-induced atherosclerosis through reprogramming gut microbiota-mediated choline metabolism in APOE-/- mice	Xiaoqiang Zhu et al	2022	Apoe-/- mice RAW264.7 cells	This study investigated the effects of chronic oral administration of E171 on the development of AS in APOE-/- mice fed NCD or HCD.	In conclusion, this study revealed that long-term exposure to E171 aggravated AS progression through enhancing TMA and TMAO generation, particularly in HCD-fed AS-prone mice . Further analysis demonstrated that E171 remodeled the gut microbiota to produce more TMA and TMAO from dietary choline. To the best of our knowledge, this is the first study showing the effects of TiO2 particles on the TMA-produced bacteria strains and its association with AS development.

Ligustrum Robustum Alleviates Atherosclerosis by Decreasing Serum TMAO, Modulating Gut Microbiota and Decreasing Bile acid and cholesterol absorption in Mice	Sijing Liu et al	2021	C57BL/6J mice Apoe-/-mice	This study investigated impacts of LR on AS development and explored the potential underlying mechanisms in C57BL/6J and Apoe-/- mice.	LR attenuated AS development presumably by decreasing serum TMAO levels and increasing fecal BA excretion likely via gut microbial modulation. These effects were accompanied by increases in fecal cholesterol excretion, decreases in serum and hepatic cholesterol.
Dietary astaxanthin-rich extract ameliorates atherosclerosis/retinopathy and restructures gut microbiome in apolipoprotein E-deficient mice fed on a high-fat diet	Dong Liu et al	2022	ApoE−/−)mice C57BL/6J mice	This study is aimed to investigate the function of ASTE on AS and gut microbiota as well as the difference from ATO in apolipoprotein ApoE−/− mice.	This study results suggested that ASTE could prevent AS in both macrovascular and/or microvascular as well as used as novel prebiotics by supporting the bile acid excretion and growth of *Akkermansia*.
Long-chain monounsaturated fatty acids improve endothelial function with altering microbial flora	RIE TSUTSUMI et al	2021	Human ApoE-/-mice	This study investigated whether LCMUFAs could improve endothelial functions in mice and humans.	These data suggest that LCMUFAs alter the microbiota environment that stimulate the production of SCFAs, resulting in the induction of GLP-1 secretion. Fish oil-derived long-chain monounsaturated fatty acids might thus help to protect against cardiovascular disease.
Ameliorative effect of purified anthocyanin from Lycium ruthenicum on atherosclerosis in rats through synergistic modulation of the gut microbiota and NF-κB/SREBP-2 pathways	Yang Luo et al	2019	SD rats	Ameliorative effect and potential mechanism of purified anthocyanins from LRPA on AS were investigated.	The study demonstrated that oral administration of LRPA significantly ameliorated HFD- and VD3-induced AS. The improvement contributed to the reshaping of the destructed gut microbiota community and the modulation of signaling pathways of arterial inflammation (NF-kB) and hepatic lipid metabolism (SREBP-2).

Bowman-Birk Major Type Trypsin Inhibitor Derived from Foxtail Millet Bran Attenuate Atherosclerosis via Remodeling Gut Microbiota in ApoE−/− Mice	Shuhua Shan et al	2022	THP-1 cell ApoE−/− mice	In this study, a novel Bowman-Birk type major trypsin inhibitor from FMB-BBTI with an anti-AS effect was obtained by in vitro gastrointestinal bionic digestion.	In conclusion, a new pharmacological function of FMBBBTI derived from foxtail millet bran protein hydrolysates in anti-AS activity was discovered. The results showed that FMBBBTI administration effectively attenuated the development of AS, characterized by the reductions of lipid phagocytosis, lesion areas of aortic sinus and atherosclerotic plaque in the aorta, and the decrease of levels of major inflammatory cytokines TNF-α and IL-1β in vitro and in vivo. Interestingly, the remodeling of gut microbiota contributed to the anti-AS effect of FMB-BBTI in the apoE−/− mice AS model, characterized by the growth of Firmicutes at the phylum level, and at the genus level, the declining abundance of *[Ruminococcus]* and *Allobaculum*, and especially, the significant enrichment of Lactobacillus. This study highlights in elucidating the anti-AS effect of FMB-BBTI from the perspective of reshaping gut microbiota.
Capsaicin Ameliorates High-Fat Diet-Induced Atherosclerosis in ApoE−/− Mice via Remodeling Gut Microbiota	Zijian Dai et al	2022	ApoE−/− mice	This study aimed to investigate the role of gut microbiota in ameliorating the effect of capsaicin on AS.	In summary, this study found that dietary administration of capsaicin could prevent the development of atherosclerosis, reduce serum lipids, proinflammatory cytokines, and LPS levels, and improve HFD-induced inflammatory response in the colon. However, all these beneficial effects of capsaicin were abolished after an antibiotic intervention. Capsaicin administration also significantly altered the composition of gut microbiota and cecal metabolomic profiles, which might contribute to the improvement of atherosclerosis. Although the relationship of gut microbiota induced by capsaicin administration with atherosclerosis still needs further study, such as fecal bacteria transplantation and vitro fermentation experiments, the present study provides a new theoretical basis for the

					antiatherosclerosis effect of capsaicin.
Eicosapentaenoic Acid-Enriched Phosphoethanolamine Plasmalogens Alleviated Atherosclerosis by Remodeling Gut Microbiota to Regulate Bile Acid Metabolism in LDLR−/− Mice	Lin Ding et al	2020	LDLR−/− mice	In the study, the effect of EPA-PlsEtns on the gut microbiota−liver−bile acids axis was investigated by determining bile acid profiles, hepatic key enzymes involved in bile acids metabolism, and gut microbiota composition in LDLR−/− mice with atherosclerosis.	In conclusion, EPA-PlsEtns rather than EPA-EE exhibited a superior effect on ameliorating atherosclerosis via lowering cholesterol levels, which might be mainly attributed to the increasing bile acid synthesis from excess cholesterol in the liver. The process might be associated with the regulation of microbiota, secondary bile acid biotransformation, and individual bile acid proportion via suppressing FXR activation and the negative-regulation of the CYP7A1 expression.

Four Citrus Flavanones Exert Atherosclerosis Alleviation Effects in ApoE−/− Mice via Different Metabolic and Signaling Pathways	Feng Wang	2021	ApoE−/− mice C57BL/6 J mice	The atherosclerosis-alleviating effects of four citrus flavanones (NR, NG, HD, and HT) were investigated in ApoE−/− mice in the present study. How in vivo metabolism of the four citrus flavanones promoted the alleviation of atherosclerosis was further investigated.	In summary, the in vivo metabolism and anti-atherosclerosis signaling pathways of four citrus flavanones (naringin, naringenin, hesperidin, and hesperetin) were compared in ApoE−/− mice. Naringin had the most potent anti-atherosclerosis effect, followed by hesperidin, naringenin, and hesperetin. Naringin mainly existed in the intestinal tract after oral administration, which might be due to the high water-solubility of 7-O-nohesperidoside.It promoted bile acid synthesis by regulating CYP7A1 expression via the gut microbiota-FXR/FGF15 pathway. Some naringin absorbed in the liver downregulated PCSK9 expression to enhance cholesterol reverse transport. The other three citrus flavanones mainly alleviated atherosclerosis in the liver after absorption from the intestine. Hesperidin, which possesses a 7-Orutinoside group, was rapidly absorbed from the intestine after oral intake, and upregulated the expression of ABCA1 and enhanced cholesterol reverse transport from peripheral tissues to the liver. Naringenin and hesperetin were mainly absorbed by the small intestine and downregulated HMGCR to suppress cholesterol synthesis. Notably, hesperetin was more resistant to absorption than naringenin due to the existence of a 4′methoxyl group, and had relatively weak effects on atherosclerosis. The results provide insight into the antiatherosclerosis mechanisms of food functional flavanones and guidance for the design and development of novel strategies for preventing and treating atherosclerosis based on citrus flavanones.

Gallic acid ameliorates atherosclerosis and vascular senescence and remodels the microbiome in a sex-dependent manner in ApoE−/− mice	McKenzie Clark et al	2022	ApoE−/− mice<br>Vascular smooth muscle cells	The study investigates whether GA, a polyphenol abundant in blackberry, decreases plaque and whether its effect is also sex-dependent.	In conclusion, the study demonstrates that GA reduces plaque burden in male ApoE−/− mice in the arch and descending aorta. Similar to our blackberry study, the reduction in plaque was not correlated with modulation of the lipid profile and was not seen in females. Further, GA partly restored the microbiome composition by ameliorating HFD-induced gut dysbiosis in males. These findings suggest that the consumption of foods rich in GA may reduce the incidence and progression of atherosclerosis, which may be dependent on sex and the composition of the gut microbiota.
Gut microbiome and metabolomic profiles reveal the antiatherosclerotic effect of indole-3-carbinol in high-choline-fed ApoE-/- mice	Yuan He et al	2024	C57/BL6 mice<br>ApoE-/- mice<br>Mouse macrophages (RAW264.7)	The objective of this research was to investigate the impact of I3C on choline-induced AS and to further elucidate the underlying mechanism involved.	In summary, I3C from cruciferous vegetables suppressed the development of atherosclerosis caused by high-choline intake, enhanced the diversity of the gut microbiota and the abundance of the phylum *Verrucomicrobia*, and consequently modified the metabolites produced by gut microbes. Further metabolomic and bioinformatic analyses revealed that m1A is a key modulator of the protective effect of I3C on AS progression in high-choline-fed ApoE-/- mice. The species abundance and diversity of the gut flora are key factors for preventing atherosclerotic plaque formation; however, the detailed molecular targets of I3C and their signal transduction mechanisms still require further investigation. Therefore, this study explored the potential of I3C to promote human health, and may reduce the clinical severity of AS-related cardiovascular diseases.

Helianthus Annuus L. Alleviates High-Fat Diet Induced Atherosclerosis by Regulating Intestinal Microbiota, Inhibiting Inflammation and Restraining Oxidative Stress	Jianbing Wang et al	2021	Apoe-/- mice	The objective of this study was to investigate the anti-atherosclerosis effect and the related mechanism of HAL.	In conclusion, the study have demonstrated that HAL alleviates atherosclerotic plaque formation by inhibiting inflammation and restraining oxidative stress in the AS mouse model. This study uncovered that HAL reduced the inflammatory response of AS through regulating the intestinal microbiota and reducing intestinal permeability. Further studies are required for proof of concept in human AS.
Quinic acid regulated TMA/TMAO-related lipid metabolism and vascular endothelial function through gut microbiota to inhibit atherosclerotic	Qiao Jin et al	2024	Apoe−/− mice<br>Human coro-nary artery endothelial cells	This study attempted to investigate the mechanism of QA on atherogenesis in Apoe−/− mice induced by HFD.	QA regulated the gut-liver lipid metabolism and chronic vascular inflammation of TMA/TMAO through gut microbiota to inhibit the atherogenesis in Apoe−/− mice, and the mechanism may be related to the HMGB1/ SREBP2 pathway.
Mangiferin alleviates trimethylamine-N-oxide (TMAO)-induced atherogenesis and modulates gut microbiota in mice	Zouyan He et al	2023	ApoE−/− mice	The study aimed to investigate the effect of mangiferin on TMAO-induced atherogenesis in mice fed a high-choline diet.	In conclusion, mangiferin exhibited its ability to alleviate TMAO-induced atherosclerosis through its anti-inflammatory, cholesterol-lowering, and gut microbial modulatory activities.
Millet shell polyphenols prevent atherosclerosis by protecting the gut barrier and remodeling the gut microbiota in ApoE−/− mice	Fengming Liu et al	2021	Human aortic smooth muscle cells<br>ApoE−/− mice	To explore the anti-atherosclerotic activity of MSPs in vivo, a classic atherosclerosis model was constructed in ApoE−/− mice fed with a high-fat diet.	In conclusion, this study extracted polyphenols (MSPs) from millet shells, which have anti-atherosclerotic potential, and their main components include 3-hydroxybenzylhydrazine, luteolin3′,7-diglucoside, N-acetyltyramine, p-coumaric acid, vanillin, sinapic acid, ferulic acid and isophorone. In vivo studies showed that oral administration of MSPs lowers endotoxaemia by improving the barrier function. It further reduces inflammatory markers and atherosclerotic lesions. At the same time, changes in the structure and abundance of gut microbiota play a key role in this process. Hence, these results provide a new idea for the prevention of cardiovascular disease using natural products such as millet shell polyphenols.

Naringin Alleviates Atherosclerosis in ApoE−/− Mice by Regulating Cholesterol Metabolism Involved in Gut Microbiota Remodeling	Feng Wang et al	2020	ApoE−/− mice	This study investigated the gut microbiota-related mechanisms by which naringin alleviates atherosclerosis in female ApoE−/− mice fed with a high-fat diet.	In conclusion, naringin alleviated atherosclerosis in ApoE−/− mice by regulating cholesterol metabolism via gut microbiota remodeling. The alleviation of atherosclerosis by naringin was associated with modulation of bile acid biosynthesis from cholesterol via changes in CYP7A1andFXR/FGF15 expression, which were caused by modulation of the abundance of *Bacteroides, Bif idobacterium, Clostridium*, and *Eubacterium*. Atherosclerosis alleviation was also facilitated by promotion of RCT to the liver from peripheral tissues by suppressing the expression of PCSK9 and IDOL.
Atherosclerosis amelioration by allicin in raw garlic through gut microbiota and trimethylamine-N-oxide modulation	Suraphan Panyod et al	2022	C57BL/6 J mice<br>ApoE−/− mice<br>Human	This study aimed to investigate the effect of allicin and raw garlic in modulating both the function and composition of the gut microbiota for cardiovascular protection.	Thus, raw garlic juice and allicin can potentially prevent cardiovascular disease by decreasing TMAO production via gut microbiota modulation.
Peanut skin extract ameliorates high-fat diet-induced atherosclerosis by regulating lipid metabolism, inflammation reaction and gut microbiota in ApoE / mice	Mingjuan Xu et al	2022	ApoE- / - mice<br>C57BL/6J mice	This study aims to investigate the preventive effect of PSE on high-fat diet-induced AS in mice and explore the underlying mechanisms.	In conclusion, dietary consumption of PSE can obviously mitigate lipid metabolism disorder, reduce oxidative stress and inflammation level, regulate the balance of gut microbiota and thus alleviate AS damage in a high-fat and high-cholesterol induced atherosclerotic mouse model. Metabonomics analysis of the liver, serum and fecal showed that PSE may alleviate the diet-induced metabolic disorder of atherosclerotic mice through the pathways related to lipid and bile acid metabolism. Furthermore, the 16S rRNA sequencing analysis revealed that the anti-atherosclerotic effect of PSE may be partly attributed to changes in the composition and function of the gut microbiota, which may be related to the anti-inflammatory activity. In addition, different doses of PSE have different alleviating effects on AS. Overall, these results indicate that PSE may be a

					promising candidate to prevent AS.
Polyphenols from hickory nut reduce the occurrence of atherosclerosis in mice by improving intestinal microbiota and inhibiting trimethylamine N-oxide production	Chenyu Jiang et al	2024	C57BL/6 J mice	The relationship between polyphenol of hickory nut and atherosclerosis prevention will be firstly clarified, providing theoretical basis for the discoveryof natural products counteracting TMAO-induced AS process in hickory nut.	Hickory polyphenol extract can mitigate HFD-induced AS by regulating intestinal microflora in murine models. In addition, TMA-FMO3-TMAO pathway may play a key role in this process. This research unveils, for the inaugural time, the complex interaction between hickory nut-derived polyphenols and gut mi crobial, providing novel insights into the role of dietary polyphenols in AS prevention.
Protocatechuic acid alleviates TMAO-aggravated atherosclerosis via mitigating inflammation, regulating lipid metabolism, and reshaping gut microbiota	Huafang Ding et al	2024	ApoE−/− mice	The study investigated the effect of PCA on TMAO-aggravated atherosclerosis in ApoE−/− mice.	In summary, PCA could alleviate the TMAO-exacerbated atherosclerosis and inflammation, improve the lipid metabolism, and modulate gut microbiota.
Amelioration of Atherosclerosis by lycopene is linked to the modulation of gut microbiota dysbiosis and related gut-heart axis activation in high-fat diet-fed ApoE−/− mice	Tengcan Tu et al	2023	ApoE−/− mice	This study surmised that lycopene could regulate the gut microbiota, exert anti-atherosclerotic effect by regulating the “gut-heart” axis.	This study results indicated the protective effect of lycopene against atherosclerosis induced by HFD and further revealed that its mechanism might be its prebiotic effect on maintaining gut microbiota homeostasis and improving intestinal barrier function, consequently reducing serum LPS-triggered inflammatory response in the heart.

Anti-atherosclerotic effects of geraniin through the gut microbiota-dependent trimethylamine N-oxide (TMAO) pathway in mice	Kaiyang Lin et al	2022	ApoE / mice	This study aimed to investigate the pharmacological activity of geraniin in atherosclerosis through remodeling the gut microbiota.	Geraniin might be an effective prospective drug against cardiovascular diseases, and the gut microbiota is a potential target to reduce the risk of atherosclerotic disease.
Berberine attenuates choline-induced atherosclerosis by inhibiting trimethylamine and trimethylamine-N-oxide production via manipulating the gut microbiome	Xingxing Li et al	2021	C57BL/6J mice ApoE KO mice	The goal of this study is to examine the role of BBR in gut microbiota remodeling and TMA/ TMAO generation in C57BL/6J and ApoE KO mice with atherosclerosis induced by choline-supplemented chow diet.	These results offer new insights into the mechanisms responsible for the anti-atherosclerosis effects of BBR, which inhibits commensal microbial TMA production via gut microbiota remodeling.
Butanol Extract of Acanthopanax senticosus (Rupr. etMaxim.) Harms Alleviates Atherosclerosis inApolipoprotein E-Deficient MiceFed a High-Fat Diet	Ailing Jia et al	2023	ApoE -/ -mice C57BL/6 mice	This study investigated the effect of Butanol Extract of ASBUE on atherosclerosis in ApoE - / - mice.	This study findings demonstrated the antiatherosclerotic potential ofASBUE, which ismediated bythe interaction between the gut microbiota and lipid metabolism and regulated viathe NF-κB pathway.
Crocin mitigates atherosclerotic progression in LDLR knockout mice by hepatic oxidative stress and inflammatory reaction reduction, and intestinal barrier improvement and gut microbiota modulation	Shufen Han et al	2022	C57BL/6 mice LDLR-/- mice	This study aimed to explore the effects of crocin supplementation on atherosclerosis, and to assess the possible underlying mechanisms via targeting on hepatic oxidative stress and inflammatory reaction, and modulating intestinal barrier and gut microbiota.	These results suggest that crocin can ameliorate inflammation and attenuate atherosclerotic progression by oxidative stress reduction and intestinal barrier improvement.
Curcumin attenuates cadmium-induced atherosclerosis by regulating trimethylamine-N-oxide synthesis and macrophage polarization through remodeling the gut microbiota	Jiexin Zhang et al	2022	ApoE-/- mice	This study assess the effect of Cd exposure on gut flora, TMAO metabolism and macrophage polarization, further investigate whether curcumin protects against Cd-induced AS by remodeling gut microbiota.	This study first demonstrate that Cd exposure worsens the progression of AS via intestinal flora imbalance and increased TMAO synthesis. Curcumin was verified as a potential novel intervention for preventing Cdinduced AS via remodeling gut microbiota. This study elucidates a new approach for treating AS in regions with significant Cd exposure.
Gypenoside XLIX Ameliorate High-Fat Diet-Induced Atherosclerosis via Regulating Intestinal Microbiota, Alleviating Inflammatory Response and Restraining Oxidative Stress in ApoE−/− Mice	Ming Gao et al	2022	ApoE−/− mice	The aim of this study is to explore the anti-atherosclerosis effect of GPE on the inflammation and intestinal microbiota in a high-fat diet and atherosclerosis induced by choline in ApoE-/-mice.	This study found that gypenoside XLIX can reduce atherosclerosis and the anti-atherosclerotic effect of GPE is related to the substantial changes in intestinal microbiota and anti-inflammatory activity. This can provide a certain theoretical basis for the treatment of atherosclerosis with Chinese herbal medicine.

Ginkgo biloba extract ameliorates atherosclerosis via rebalancing gut flora and microbial metabolism	Yun Wang et al	2022	Ldlr-/-mice	This study was designed to explore the antiatherogenic mechanism of GbE from the perspective of the gut microbial regulation.	In conclusion, this study demonstrated that oral administration of GbE effectively alleviates hyper cholesterolemia, systemic inflammation, and atherosclerosis and these effects are associated with the modulation of gut microbial taxonomic composition, protection of the intestinal mucosal integrity and improvement of the microbial metabolic phenotypes including restoring SCFAs, IAA, and secondary bile acids production.
Ginkgolide B treatment regulated intestinal flora to improve high-fat diet induced atherosclerosis in ApoE / mice	Zhiyang Lv et al	2021	ApoE -/ -mice	This study aimed to explore whether GB alleviated AS and the underlying mechanisms of GB on intestinal flora.	In summary, HFD-induced dyslipidemia, inflammation, increased atherosclerotic plaque and gut barrier dysfunction could be reduced by GB treatment. Considering these previous results as well as this study, the study firstly support that the therapeutic value of GB treatment substantially reduced HFD-induced AS by the relative abundance of higher Bacteroides and lower Helicobacter, but its exact mechanism still need to carry out further research. These results suggest that the therapeutic value of GB on gut microbiota manipulation in treating AS, but it still requires further investigation.
Ginsenosides retard atherogenesis via remodelling host–microbiome metabolic homeostasis	Yun Wang et al	2024	Ldlr-/-mice C57BL/6J mice	This study aims to clearly define the efficacy and underlying mechanism of P. ginseng and its active components in protecting against atherosclerosis.	This study provides strong evidence supporting TGS and ginsenoside Rb1/Rg1 combinations as effective therapies against atherogenesis, via targeting different signal nodes by different components and may provide some elucidation of the holistic mode of herbal medicines.
Grape Seed Proanthocyanidin Extract Alleviates Atherosclerosis by Modulating the Production of Trimetlylamine N-Oxide	Guofu Wang et al	2023	ApoE-/- mice	This study aims to explore the effect of GSPE on the TMAO content and atherosclerosis.	This study demonstrates that high doses of GSPE exert anti-atherosclerosis effects by reducing TMAO content to modulate the disturbed gut microbiota.

Ferulic Acid Ameliorates Atherosclerotic Injury by Modulating Gut Microbiota and Lipid Metabolism	Yuyan Gu et al	2021	ApoE−/−mice C57BL/6 mice	This study aimed to investigate the effects of FA on the gut microbiota and lipid metabolism in the atherosclerotic mice and clarify its molecular mechanism.	The study has shown that FA treatment decreases serum lipids and reduces atherosclerotic plaques in ApoE−/− mice. FA also modulates the composition of gut microbiota and fecal metabolites, which is closely related to atherosclerosis. In addition, FA regulates lipid metabolism through activation of the AMPKα/SREBP1/ACC1 pathway in the liver. In brief, the study demonstrate that FA could significantly ameliorate atherosclerotic injury, which may be partly by modulating gut microbiota and lipid metabolism via the AMPKα/SREBP1/ACC1 pathway. Nevertheless, the direct link of FA on gut microbiota and atherosclerosis requires further studies.
Guggulsterone, a farnesoid X receptor antagonist lowers plasma trimethylamine-N-oxide levels: An evidence from in vitro and in vivo studies	A Gautam et al	2019	Wistar albino rats	The study investigated the role of GS, a farnesoid X receptor antagonist, in the choline metabolism and its TMA/TMAO inhibiting potential in a series of in vitro and in vivo studies as determined by HPLC, MS, and LC-MS techniques.	In summary, this study found that 2% choline fed diet induces AS in the current study. The supplementation with GS acts on the TMA/TMAO/FMO3 pathway and subsequently reduces the level of TMAO through the inhibition of FMO3 expression in both in vitro and in vivo analysis. Moreover, GS exhibited an antihyperlipidemic property, reduced the lipid peroxidation process, and enhanced the antioxidant defense system. In addition, GS exhibits a strong cardioprotective effect as demonstrated by a significant reduction of several AIs.
Gut Microbiota Composition Affects Procyanidin A2-Attenuated Atherosclerosis in ApoE−/− Mice by Modulating the Bioavailability of Its Microbial Metabolites	Shiying Yang et al	2021	C57BL/6 mice ApoE−/− mice	This study examined the effect of PCA2 on HFD-induced AS in ApoE−/− mice with an intact and antibiotic-depleted microbiota.	In conclusion, PCA2 attenuated lipid metabolism disorder, oxidative stress, and inflammation, thus attenuating AS progression in ApoE−/− mice fed with HFD through reshaping the gut microbiota. The intervention of antibiotics significantly reduced the bioavailability of PCA2-derived metabolites and prevented PCA2 from exerting anti-AS activity. These results indicated that the association of PCA2-influenced gut microbiota composition with the anti-AS property was mediated by

					its considerable effect on the plasma concentration of PCA2-derived phenolic acid metabolites, which drive the anti-AS property of PCA2.
Gut Parabacteroides merdae protects against cardiovascular damage by enhancing branched-chain amino acid catabolism	Shanshan Qiao et al	2022	ApoE−/− mice C57BL/6J mice	This study hypothesized that GMD could have beneficial effects against atherosclerosis through modulating the gut microbiota.	In summary, this study provide evidence for the beneficial effects of GMD and the gut commensal bacterium P. merdae against obesity-related atherosclerosis. The targeted modulation of gut microbiota represented by the enrichment of gut commensal P. merdae was demonstrated to contribute to the anti-atherosclerosis effects of GMD by mediating the catabolism of BCAAs in the gastrointestinal tracts.
Inflammation inhibition and gut microbiota regulation by TSG to combat atherosclerosis in ApoE−/− mice	Fengjiao Li et al	2020	ApoE-/- mice	The aim of this research was to study the effects of PMRP and its major active chemical constituent TSG on AS in ApoE-/- mice fed with high fat diets to provide a scientific basis in the use of PMRP and TSG against cardiovascular diseases.	PMRP and TSG improved lipid accumulation and inflammation, and regulated the intestinal microbial imbalance in ApoE-/- mice. TSG exerted a preventive effect in the development and progression of AS.
Isolation of melanoidins from heat-moisture treated ginseng and its inhibitory effect on choline metabolism	Rui Liu et al	2023	C57BL/6Jmice	The melanoidins were isolated from heat-moisture treated ginseng to explore its structural features and the preventive effect on RM induced AS in mice.	The findings indicate melanoidins isolates may inhibit choline metabolism by co-regulating gut microbiota and the AMPK pathway.
Gut microbiota and diet matrix modulate the effects of the avonoid quercetin on atherosclerosis	Federico Rey et al	2023	C57BL/6 mice ApoE KO mice	This study assessed the influence of the gut microbiota and diet matrix on quercetin-mediated athero-protection.	This study suggest that the beneficial effects of quercetin on atherosclerosis are influenced by gut microbes and dietary MAC.
Puerarin alleviates atherosclerosis via the inhibition of *Prevotella copri* and its trimethylamine production	Ze-Hua Li et al	2024	Human ApoE−/− mice	This study aimed to investigate the effects of PU and its mechanisms in mitigating AS in both mice and humans.	PU may provide therapeutic benefits in combating AS by targeting P. copri and its production of TMA.

Integration of 16S rRNA sequencing and metabolomics to investigate the modulatory effect of ginsenoside Rb1 on atherosclerosis	Yuqin Liang et al	2024	SD rats	The aim of this study was to investigate the mechanisms of Rb1 in ameliorating AS induced by HFD.	Rb1 ameliorated the HFD-induced AS, and the mechanism is related to improving intestinal metabolic homeostasis and inhibiting systemic inflammation by regulating gut microbiota.
Usnea improves high-fat dietand vitamin D3-induced atherosclerosis in rats by remodeling intestinal flora homeostasis	Yanjun Liu et al	2022	SD rats	To investigate the potential mechanisms underlying the anti-AS activity of UEE via the regulation of intestinal flora.	The underlying mechanism was the reversal of imbalances in the intestinal flora by Usnea, thereby inhibiting calcium deposition, abnormal lipid metabolism, and inflammatory response.
Berberine treats atherosclerosis via a vitamine-like effect downregulating Choline-TMA-TMAO production pathway in gut microbiota	Shu-Rong Ma et al	2022	SD rats<br>Hamsters<br>Human	This study investigates whether or not BBR could reduce TMAO production in the gut microbiota and treat atherosclerosis.	BBR might treat atherosclerotic plaque at least partially through decreasing TMAO in a mode of action similar to that of vitamins.
Noto ginsenoside R1 Ameliorate High-Fat-Diet and Vitamin D3-Induced Atherosclerosis via Alleviating Inflammatory Response, Inhibiting Endothelial Dysfunction, and Regulating Gut Microbiota	Liying Ma et al	2024	SD rats	The study was performed to investigate the impacts of a natural drug component, notoginsenoside R1, on the development of AS and the potential mechanisms.	Notoginsenoside R1, due to its unique anti-inflammatory properties, may potentially prevent the progression of atherosclerosis. This mechanism helps protect the vascular endothelium from damage, while also regulating the imbalance of intestinal microbiota, thereby maintaining the overall health of the body.
Microbiome and metabonomics study of quercetin for the treatment of atherosclerosis	Dong-Ning Wu et al	2019	ApoE−/− mice<br>C57BL/6J mice	This study aimed to explore the gut microbial and metabolic signatures of quercetin in AS treatment and conduct an integrative analysis on its biomechanism.	This study explored the gut microbial and metabolic involvement of quercetin in AS treatment and suggest the association between AS and gut metabolic regulation.
Paeonol reduces microbial metabolite α-hydroxyisobutyric acid to alleviate the ROS/TXNIP/NLRP3 pathway-mediated endothelial inflammation in atherosclerosis mice	LIU Yarong et al	2023	ApoE−/− mice	To explore the mechanism by which Pae reduces the harmful metabolites of the gut microbiota to alleviate AS.	This study innovatively confirms the mechanism by which Pae reduces the harmful metabolites of gut microbiota to alleviate AS and proposes HIBA as a potential biomarker for AS clinical judgment.
Ginger essential oil and citral ameliorates atherosclerosis in ApoE−/− mice by modulating trimethylamine-N-oxide and gut microbiota	Suraphan Panyod et al	2023	ApoE−/− mice	This study elucidated the anti-atherosclerotic effect and mechanism of GEO (Zingiber officinale Roscoe) and its bioactive compound citral in GAN diet with ʟ-carnitine-induced atherosclerosis female ApoE−/− mice.	This study showed that GEO and citral may serve as potential dietary supplements for CVD prevention by improving gut microbiota dysbiosis.

Quercetin reduces atherosclerotic lesions by altering the gut microbiota and reducing atherogenic lipid metabolites	J. Nie et al	2019	Ldlr–/– mice	This study examined the hypothesis that the oral administration of quercetin to low-density lipoprotein receptornull (Ldlr-/-) mice would improve gut health by altering the gut microbiota and controlling the levels of atherogenic lipid metabolites and proinflammatory mediators in the intestine and serum.	This study demonstrated the ability of quercetin treatment to reduce lipid levels, as well as the areas of atherosclerotic lesions and sizes of plaques. This treatment also altered the composition of the gut microbiota and decreased the levels of atherogenic lipid metabolites.
Remodelling of gut microbiota by Berberine attenuates trimethylamine N-oxide-induced platelet hyperreaction and thrombus formation	Zulong Xie et al	2021	SD rats	This study aimed to investigate whether berberine affects choline diet-induced arterial thrombosis and explore the potential mechanism.	This study demonstrate that berberine attenuates the risk of choline diet-induced arterial thrombosis by changing the gut microbial composition and reducing TMAO generation.
Paeonol Attenuated Vascular Fibrosis Through Regulating Treg/Th17 Balance in a Gut Microbiota-Dependent Manner	Xiaoyan Shi et al	2021	ApoE-/- mice<br>C57BL/6 mice	To investigate the antifibrosis effect of Pae on AS mice and demonstrate the underlying gut microbiota-dependent mechanism.	Pae attenuated vascular fibrosis in a gut microbiota-dependent manner. The underlying protective mechanism was associated with the improved Treg/Th17 balance in spleen mediated through the increased microbiota-derived SCFA production. This study demonstrated the role of Pae as a potential gut microbiota modulator to prevent and treat AS.
Inhibiting vascular smooth muscle cell proliferation mediated by osteopontin via regulating gut microbial lipopolysaccharide: A novel mechanism for paeonol in atherosclerosis treatment	Xiaoyan Shi et al	2022	Human<br>ApoE−/− mice<br>C57BL/6J mice<br>Vascular smooth muscle cells<br>THP-1 cells	To investigate a potential mechanistic link between the gut microbial LPS and VSMC proliferation in atherosclerosis progression and explore the possible role of Pae.	Overall, this study data show that microbiota dysbiosis affects microbial LPS producing, leading to increased expression of OPN in circulating monocyte/macrophages and promoted VSMC proliferation. This might be the pathway, at least in part, that links microbiota dysbiosis to atherosclerosis.
Astragaloside IV Mediates the PI3K/Akt/mTOR Pathway to Alleviate Injury and Modulate the Composition of Intestinal Flora in ApoE−/− Atherosclerosis Model Rats	Dongwen Sun et al	2024	ApoE−/− SD rats<br>SD rats	This research aims to investigate the outcome of AST IV on AS and its potential molecular mechanism.	AST IV has a potential anti-AS effect, which can improve the pathological changes of the aorta in ApoE−/− rats fed with a high-fat diet, reduce the level of inflammatory factors, and modulate the composition of intestinal flora via the PI3K/Akt/mTOR pathway.
The Role of Ophiopogonin D in Atherosclerosis: Impact on Lipid Metabolism and Gut Microbiota	Ya-Xin Zhang et al	2021	ApoE−/− mice<br>C57BL/6 mice<br>Human LO2 cells	This study aimed to examine the efficacy of OPD in the prevention of atherosclerosis.	This study illustrate that OPD could significantly protect against atherosclerosis, which might be associated with the moderation of lipid metabolism and alterations in gut microbiota composition and fecal metabolites.

Thelenota ananas saponin extracts attenuate the atherosclerosis in apoE−/− mice by modulating lipid metabolism	Qi-An Han et al	2019	ApoE−/− mice	Accordingly, this study was designed to further investigate the effects and mechanisms of T. ananas saponin extracts on the lipid metabolism and intestinal microbial diversity in apoE−/− mice, with the aim of providing a theoretical foundation of T. ananas saponin extracts in preventing CAD caused by hyperlipidemia in humans.	This study showed that saponins extracted from T. ananas alleviated the AS progress in apoE−/− mice partly by regulating lipid metabolism and gut microbial community. The saponin extracts significantly inhibited the formation of arterial atherosclerotic plaque and reduced the concentrations of lipid profiles in both plasma and adipose tissue. This study also suggested that saponins modulated key protein expressions in the liver related to lipid metabolism. More importantly, the saponins also played an essential role in the diversity of the murine gut microbial community, which is closely related to cholesterol level and inflammation.
Tilianin improves lipid profile and alleviates atherosclerosis in ApoE / mice through up-regulation of SREBP2-mediated LDLR expression	Yu Du et al	2023	C57BL/6 mice<br>ApoE- / -mice<br>Human hepatoma HepG2 cells	The purpose of this study is to investigate the efficiency and underlying mechanisms of tilianin in controlling lipid profile and preventing atherosclerosis.	This study demonstrated the lipid-lowering effect of tilianin through SREBP2-mediated transcriptional activation of LDLR. Our findings reveal a novel anti-atherosclerotic mechanism of tilianin and underlie its potential clinical use in modulating CVDs with good availability and affordability.
Effect of Berberine on Atherosclerosis and Gut Microbiota Modulation and Their Correlation in High-Fat Diet-Fed ApoE−/− Mice	Min Wu et al	2020	ApoE−/− mice<br>C57BL/6J mice	The purposes of this study were to observe the effects of high or low doses of BBR on atherosclerosis and gut microbiota modulation, and to explore their correlation in ApoE−/− mice fed a high-fat diet.	In conclusion, both high and low dose BBR can improve serum lipid and inflammation levels and alleviate atherosclerosis in a mouse model of HFD-induced atherosclerosis. This antiatherosclerotic effect of BBR may be partly attributed to changes in composition and functions of gut microbiota which may be related to anti-inflammatory activity and the metabolism of glucose and lipid. In addition, different doses of BBR had different effects, among which the attenuation of atherosclerosis was more obvious at the high dose of BBR, but the alterations of composition and functions in gut microbiota showed different sensitivity to BBR dose.

β-sitosterol inhibits trimethylamine production by regulating the gut microbiota and attenuates atherosclerosis in ApoE−/− mice	Weiping Wu et al	2022	ApoE−/− mice	This study aimed to investigate whether β-sitosterol can inhibit TMA production in ApoE−/− mice by reshaping the gut microbial structure.	This study demonstrated that β-sitosterol could inhibit trimethylamine production, attenuate AS, alleviate the inflammatory response and improve antioxidant defense capacity. Besides, β-sitosterol has beneficial effects on gut microbial communities, which is responsible for the major pathway for TMA production. This study was concluded that the microbiota-metabolism-immunity axis might play a pivotal role in the therapeutic mechanism of β-sitosterol.
Ginsenoside Rc ameliorated atherosclerosis via regulating gut microbiota and fecal metabolites	Bin Xie et al	2022	ApoE−/− mice	the study aimed to examine the anti-atherosclerotic effects and potential mechanisms of GRc against AS and provide a candidate small molecule for the prevention and treatment of CVDs.	In this study, GRc reduced atherosclerotic lesions, improved lipid levels, and systemic inflammation in HFD-induced ApoE−/− mice without significant change in body weight. Considering the poor bioavailability of GRc in oral administration, GRc exerted an anti-atherosclerotic role probably through comprehensive effects of regulating gut microbiota and differentially expressed fecal metabolites closely related to CVDs. This study for the first time reported the anti-atherosclerotic effect of GRc in ApoE−/− mice induced with HFD. However, metabolites in serum and the role of some other intestinal floras regulated by GRc are still to be clarified.
Banxia Xiexin decoction alleviates AS co-depression disease by regulating the gut microbiome-lipid metabolic axis	Xing-Xing Liao et al	2023	C57BL/6 mice ApoE- /- mice	This study sought to evaluatethe effectiveness and action mechanism of BXD in regulation of the gut microbiome via an intervention in AS co-depression mice.	BXD may exert an anti-AS co-depression therapeutic effect by modulating the abundance of some flora and thus intervening in peripheral lipid and brain lipid metabolism (via downregulation of LPC levels).
Buyang Huanwu Decoction Alleviates Atherosclerosis by Regulating gut Microbiome and Metabolites in Apolipoprotein E- deficient Mice fed with High- fat Diet	Qun Yu et al	2024	C57BL/6J mice ApoE- /- mice	This study aimed to determine whether BHD could alleviate atherosclerosis by altering the microbiome- associated metabolic changes in atherosclerotic mice.	BHD treatment effectively slows the progression of atherosclerosis by regulating altered intestinal microbiota and perturbed metabolites.

Dingxin Recipe IV attenuates atherosclerosis by regulating lipid metabolism through LXR-α/SREBP1 pathway and modulating the gut microbiota in ApoE-/- mice fed with HFD	Yaxin Zhang et al	2021	C57BL/6J mice ApoE-/- mice	This study aimed to illustrate whether DXR IV improve atherosclerosis through modulating the lipid metabolism and gut microbiota in atherosclerosis mice.	DXR IV exhibits potential anti-atherosclerosis effect, which is closely related to lipid metabolism and the gut microbiota. This study may provide novel insights into the mechanism of DXR IV on atherosclerosis and a basis for promising clinical usage.
HuangQi ChiFeng decoction maintains gut microbiota and bile acid homeostasis through FXR signaling to improve atherosclerosis	Jiaqi Fu et al	2023	C57BL/6J mice ApoE–/– mice	With gut microbiota as the core of the study and bile acid metabolismas the main line of research,we explored the association between HQCFT regulation of gut microbiota and bile acid metabolism. The mechanism of HQCFT in treating AS is explored from the perspective of targeting gut microbiota to reverse the disorder of bile acid metabolism.	HQCFT may play a part in the prevention of atherosclerosis by inhibiting the FXR/LXRα axis, increasing the expression of CYP7A1 in the liver, and regulating the interaction between the gut microbiota and bile acid metabolism.
Guanxin Xiaoban capsules could treat atherosclerosis by affecting the gut microbiome and inhibiting the AGE–RAGE signalling pathway	Yin Dongliang et al	2022	ApoE–/– mice	The aim of this study was to identify genes and pathways targeted by active ingredients in Guanxin Xiaoban capsules for the treatment of atherosclerosis based on network pharmacology and analysis of changes to the gut microbiome.	Guanxin Xiaoban capsules may improve atherosclerosis and reduce the plaque area by inhibiting the AGE-RAGE signalling pathway to delay the development of atherosclerosis. This mechanism appears to involve changes in the gut microbiota. Therefore, Guanxin Xiaoban capsules have potential value as a treatment for atherosclerosis.
Guanxinning Tablet Attenuates Coronary Atherosclerosis via Regulating the Gut Microbiota and Their Metabolites in Tibetan Minipigs Induced by a High-Fat Diet	Qinqin Yang et al	2022	Tibetan minipigs	The study was aimed at investigating the therapeutic effect of GXNT on CA and further explore the underlying mechanisms from the perspective of gut microbiota.	GXNT has the functions of regulating blood lipids, antioxidative stress, antivascular endothelial injury, inhibiting platelet aggregation, anti-inflammatory, and reducing AS plaque area, thereby exerting an anti-CA effect. The mechanism may be closely related to regulating the composition of the gut microbiota and bacterial metabolites such as TMAO, SCFAs, and BAs. This study found that GXNT could modulate the relative abundance of *Proteobacteria, Enterobacteriaceae, Escherichia, Prevotellaceae,* and *Prevotella,* possibly involved in AS development, which may be the target bacteria for GXNT in AS therapy. The experiment provides an

					experimental basis for the clinical application of GXNT in the treatment of CA and new inspiration for researching the underlying mechanism. However, the composition of GXNT is complex, and its specific mechanism of action needs to be further studied.
The Enhanced Pharmacological Effects of Modified Traditional Chinese Medicine in Attenuation of Atherosclerosis Is Driven by Modulation of Gut Microbiota	Wenyan Ji et al	2020	Wistar rats C57BL/6J mice	This study developed the AS rat model, and then systematically examined i) the clinical efficiency of a novel TCM formula on AS; ii) the potential synergistic effects of the innovative TCM formula and Western medicine on treating AS, and iii) elucidated if the structural modulation of gut microbiome may causatively contribute to the significant effect of modified TCM formula exerted on AS.	This study first demonstrated the enhanced pharmacological effect of a modified TCM formula (TMZY) on AS model (HFD) rats. Furthermore, TMZY and atorvastatin could act together for the alleviation of AS in rats by suppressing immune and inflammatory responses. This study also provided the compelling evidence that alterations of gut microbiota induced by TCM formulas are associated with the anti-AS effects of TCM formulas in mice.
Naoxintong capsule remodels gut microbiota and ameliorates early-stage atherosclerosis in apolipoprotein E-deficient mice	Haofang Wan et al	2024	C57BL/6 mice ApoE- /- mice	This study is aiming to evaluate the therapeutic effect of NXT against early-stage AS, and further illustrate the potential correlations among AS, gut microbiota, and NXT.	This study demonstrated that NXT could effectively treat early-stage AS induced by HCD in mice. NXT regulated the gut microbiota and metabolites, maintained intestinal homeostasis, and improved the systemic inflammatory response. This study may provide robust experimental support for the clinical use of NXT for AS treatment.
Tongxinluo May Alleviate Inflammation and Improve the Stability of Atherosclerotic Plaques by Changing the Intestinal Flora	Yan Qi et al	2022	New Zealand white rabbits	Tongxinluo, as a multi-target Chinese medicine to improve atherosclerosis, whether it can improve atherosclerosis by affecting the intestinal flora is worth exploring.	In conclusion, Tongxinluo can improve plaque stability and reduce inflammation in atherosclerotic rabbits, which may be achieved by modulating intestinal flora and intestinal metabolism. This study provides new views for the role of Tongxinluo in improving atherosclerotic vulnerable plaque,

					which has important clinical significance.
Qing-Xin-Jie-Yu Granule alleviates atherosclerosis by reshaping gut microbiota and metabolic homeostasis of ApoE-/- mice	Anlu Wang et al	2022	C57BL/6J mice ApoE-/- mice	To investigate the protective effects of QXJYG against AS and its potential mechanisms.	This study indicate that QXJYG is effective against HFD-triggered chronic inflammation, and contributes to the alleviation of AS development, and the antiatherogenic properties of QXJYG may be partly due to the remodeling of the gut microbiota and BA metabolism.
Xinnaokang improves cecal microbiota and lipid metabolism to target atherosclerosis	R. Yang et al	2021	ApoE-/-mice	This study aims to explore the potential mechanisms of Xinnaokang in atherosclerosis treatment.	Xinnaokang effectively alleviated atherosclerosis, and this effect might be linked with the altered features of the liver metabolite profiles and cecal microbiota.
Efficacy of Spleen-and-Stomach-Tonifying, Yin-FirePurging, and Yang-Raising Decoction Derived from the Trimethylamine N-Oxide Metabolic Pathway of Intestinal Microbiota on Macrovascular Lesions Caused by Type 2 Diabetes Mellitus	Yue Yue et al	2024	Hartley-guinea pigs	This study aimed to analyze the mechanisms underlying spleen-and-stomach-tonifying, yin-fire-purging, and yang-raising decoction derived from the TMAO metabolic pathway of intestinal microbiota in the treatment of macrovascular lesions caused by T2DM.	Gavage intervention of the spleen-and-stomach-tonifying, yin-fire-purging, and yang-raising decoction can significantly improve the intestinal microbiota structure of guinea pigs, significantly reduce the serum TMAO level of guinea pigs, regulate blood lipid levels, enhance antioxidant capacity, and improve inflammatory response. Therefore, the spleen-andstomach-tonifying, yin-fire-purging, and yang-raising decoction can reduce the risk of macrovascular lesions caused by T2DM, and its mechanism may involve its capacity to regulate the TMAO metabolic pathway of intestinal microbiota.
Alisma orientalis Beverage Treats Atherosclerosis by Regulating Gut Microbiota in ApoE-/- Mice	Boran Zhu et al	2020	ApoE−/−mice	This study investigats the role of AOB-induced gut microbiota regulation in the expansion of AS.	The antiatherosclerotic effects of AOB were found associated with changes in the content of gut microbiota and a reduction in TMAO, a gut microbiota metabolite, suggesting that AOB has potential therapeutic value in the treatment of AS.

Bicyclol Alleviates Atherosclerosis by Manipulating Gut Microbiota	Xiao-Lin Li et al	2022	ApoE-/- mice	This study aimed to elucidate the effects and molecular mechanisms of BIC on AS from the perspective of gut microecology.	This study demonstrated that BIC alleviates HFD-induced AS in Apoe(−/−) mice. BIC restores the homeostasis of gut microbiota regulates gut-related biometabolites, and improves intestinal healthiness. Systemically, the immune cell dynamics and liver function were improved, leading to amended chronic inflammation and hypercholesterolemia. Consequently, BIC attenuated endothelial activation, macrophage infiltration, and CE accumulation in the aortic arch to alleviate plaque onset. The study is the first to demonstrate the antiatherosclerotic effects of BIC and indicate that the therapeutic effects of BIC on AS is at least partially mediated by gut microbiota.
Aspirin ameliorates atherosclerotic immuno-inflammation through regulating the Treg/Th17 axis and CD39-CD73 adenosine signaling via remodeling the gut microbiota in ApoE-/- mice	Zhixia Bai et al	2023	ApoE-/- mice	This study aims to address the gap in knowledge regarding the mechanisms underlying aspirin's effects on gut microbiota dysbiosis in AS, potentially providing a novel therapeutic target for AS treatment.	This study has come to the conclusion that aspirin can alleviate the development of AS by re-balancing the Th17-Treg axis and the Treg-CD39-CD73-adenosine signaling pathways. This rebalancing process appears to positively impact the restoration of gut microbiota dysbiosis and their associated metabolites in ApoE-/- mice.
Empagliflozin ameliorates atherosclerosis via regulating the intestinal flora	Han Hao et al	2023	ApoE-/- mice	This study aimed to investigate whether SGLT2i can alleviate atherosclerosis through intestinal flora.	Empagliflozin seems to mitigate atherosclerosis partly by regulating intestinal microbiota, and this anti-atherosclerotic effect can be transferred through intestinal flora transplantation.
Ticagrelor combined with aspirin displays the signature of regulating the gut 1microbiome in consistence with improving the immuno-inflammatory response in atherosclerosis	Zhixia Bai et al	2022	Human<br>ApoE-/- mice<br>C57BL/6J mice	This study performed 16S rRNA and metagenomic analysis from patients with UAP treated with ticagrelor plus aspirin or clopidogrel plus aspirin for one month to determine the composition and functions of the gut microbiome difference between the two main medications.	In conclusion, ticagrelor or ticagrelor-aspirin mediated multiple alterations of composition and function in gut microbiota, ameliorated inflammation status, and uncovered a potential mechanism of CD39-CD73-Treg associated with better AS outcomes beyond the function as P2Y12 inhibitors. This study shed new light on the implication of the intestinal microbiota–immune system–cardiovascular axis in atherosclerotic development by the intervention of ticagrelor or tiagrelor-aspirin,

					specifying the indication of ticagrelor in infection related disease via modifying the gut microbiota.
Impavido attenuates inflammation, reduces atherosclerosis, and alters gut microbiota in hyperlipidemic mice	C. Alicia Traughber et al	2023	BMDMs<br>C57BL6J mice<br>ApoE -/ -mice	This study used a WT and a hyperlipidemic mouse model of atherosclerosis to test the effects of Miltefosine on inflammasome activity, reverse cholesterol transport, atherosclerosis, and gut microbiota.	This study indicate that Miltefosine causes pleiotropic effects on lipid metabolism, inflammasome activity, atherosclerosis, and the gut microbiota.
Disulfiram Reduces Atherosclerosis and Enhances Efferocytosis, Autophagy, and Atheroprotective Gut Microbiota in Hyperlipidemic Mice	C. Alicia Traughber et al	2024	RAW-ASC cells<br>RAW-ASC-GsdmD−/−cells<br>THP-1 cells<br>THP-ASC-GFP cells<br>Jurkat cells<br>HepG2 cells<br>Human aortic endothelial cells<br>Mouse aortic smooth muscle cells<br>C57BL6J mice<br>C57BL/6J-GsdmD−/− mice<br>ApoE-/- mice	Disulfiram was recently shown to potently inhibit GsdmD, but the in vivo efficacy and mechanism of disulfiram's anti-atherosclerotic activity is yet to be explored.	This study show that disulfiram can simultaneously modulate several atheroprotective pathways in a GsdmD-dependent as well as GsdmD-independent manne.
Metformin intervention ameliorates AS in ApoE-/- mice through restoring gut dysbiosis and anti-inflammation	Ning Yan et al	2021	ApoE-/- mice	This study was aiming to contribute to the further understanding of the role of MET on the complicated interactions among gut microbiota, inflammation and metabolism in AS progression.	This study highlighted that MET treatment ameliorated AS progression through anti-inflammation and restoring gut dysbiosis in atherosclerotic ApoE-/- mice, which could contribute to the understanding of the underlying mechanism of MET in AS treatment and potentially promote MET served as an inexpensive and effective intervention for the control of the atherosclerotic cardiovascular disease.

Hydroxyurea ameliorates atherosclerosis in ApoE-/mice by potentially modulating Niemann-Pick C1-like 1 protein through the gut microbiota	Xin-Yu Yang et al	2022	C57BL/6 mice ApoE−/− mice	The goal of this study was to investigate the efficacy of hydroxyurea in high-fat diet-fed ApoE-/- mice against atherosclerosis and examine the possible mechanism underlying treatment outcomes.	In high-fat diet-fed ApoE-/- mice, hydroxyurea effectively treated atherosclerosis, lowered serum cholesterol, modulated the gut microbiota at multiple levels and affected cholesterol absorption by reducing NPC1L1 in small intestinal epithelial cells.
Cross-omics analysis revealed gut microbiomerelated metabolic pathways underlying atherosclerosis development after antibiotics treatment	Ben Arpad Kappel et al	2020	Apoe−/− mice Human	The aim of this study was to explore the interaction between gut dysbiosis by antibiotics and metabolic pathways with the impact on atherosclerosis development.	This study provides insights into the complex interaction between intestinal microbiota and host metabolism. Our data highlight that detrimental effects of antibiotics on the gut flora are connected to a pro-atherogenic metabolic phenotype beyond classical risk factors.
Reshaping of the gastrointestinal microbiome alters atherosclerotic plaque inflammation resolution in mice	Michael S. Garshick et al	2021	Apoe−/− mice	This study aimed to determine if Abx induced alterations to the intestinal microbiota interferes with atherosclerotic plaque inflammation resolution after lipid-lowering in mice.	In conclusion, in a murine model of atherosclerosis inflammation resolution, antibiotic administration that reduced diversity and shifted the *Firmicutes/Bacteroidetes* balance reduced the impact of aggressive lipid lowering on atherosclerotic plaque inflammation resolution. This study suggest a role of the microbiome in the biology of atherosclerosis regression, and possibly in the development of the disorder. Further research is required to confirm and expand on these findings.
Remodeling Intestinal Microbiota Alleviates Severe Combined Hyperlipidemia-Induced Nonalcoholic Steatohepatitis and Atherosclerosis in LDLR-/- Hamsters	Guolin Miao et al	2024	LDLR-/- Syrian golden hamsters	This study explored the impact of modulating gut microbiota through intermittent antibiotic treatment and subsequent transfer via cohousing. Additionally, this study investigated the potential benefits of supplementation with beneficial microbial metabolites. This study objective was to improve the abnormal metabolic phenotypes associated with severe CHL in both HFHC and HC diet-induced LDLR-/- hamsters.	In summary, this study is the first to show that remodeling the gut microbiota with intermittent antibiotic treatment and subsequent transfer via cohousing, and supplementing with beneficial microbial metabolites effectively alleviate NASH and atherosclerosis in both HFHC and HC diet-fed LDLR-/- hamster models, closely resembling CHL patient characteristics.

Protective effect of hydroxychloroquine on rheumatoid arthritis-associated atherosclerosis	Na Shi et al	2019	K/BxN mice	This study investigated the effect of a HFD on the development of atherosclerosis in K/BxN mice, and the effect of HCQ on amelioration of the symptoms of RA and atherosclerosis, and alterations in gut microbiota.	They mouse model of RA indicated that HFD increased ankle width and aggravated atherosclerosis and dyslipidemia, and that HCQ alleviated the dyslipidemia and atherosclerosis, but had no effect on ankle width.
Inhibition of MAOB Ameliorated High-Fat-Diet-Induced Atherosclerosis by Inhibiting Endothelial Dysfunction and Modulating Gut Microbiota	Zhen Tian et al	2023	C57BL/6 mice ApoE−/− mice HEK293 cells	However, whether MAOB regulates endothelial oxidative stress and its related mechanism and whether gut microbiota mediates the anti-atherosclerosis effect of MAOB inhibitor remains unclear.	In conclusion, this study discovered that endothelial MAOB was activated under HFD exposure, which controlled endothelial oxidative stress homeostasis and atherogenesis. This study also identified miR-3620-5p as the upstream regulator of elevated MAOB. In addition, the study provided novel insights into the protective effect of selegiline on atherosclerosis, providing evidence for selegiline as a promising therapeutic strategy for ACVDs.
Endurance exercise ameliorates Western diet–induced atherosclerosis through modulation of microbiota and its metabolites	Wen-Ching Huang et al	2022	C57BL6/J mice ApoE-/- mice	This study proposed that endurance exercise could alleviate the atherosclerosis induced by a WD and genetic deletion of ApoE through modulation of the microbiota and its associated metabolites.	In summary, this study identified the mechanisms underlying the antiatherosclerotic effects of endurance exercise exerted by the microbial community and microbial-derived SCFAs. This is the first study to demonstrate that endurance exercise can modulate microbial populations, including those of *Desulfovibrio, Tyzzerella, Lachnospiraceae_ge, Rikenellaceae,* and *Dubosiella* for anti-inflammatory activity and SCFA production; aortic inflammatory responses significantly decreased after exercise with amelioration of atherosclerotic pathogenesis.
Time to run: Late rather than early exercise training in mice remodels the gut microbiome and reduces atherosclerosis development	Milena Schönke et al	2022	APOE*3-Leiden.CETP mice	This study aimed to study whether the timing of exercise training differentially modulates the development of atherosclerosis and elucidate underlying mechanisms.	By using a well-established model of human-like cardiometabolic disease, the study show that later exercise training rather than early training attenuates diet-induced gain of fat mass and reduces atherosclerosis development. Overall, the study presented here underscores that the timing of exercise may be an important parameter in optimizing exercise recommendations for patients with

					cardiovascular diseases.
Directed remodeling of the mouse gut microbiome inhibits the development of atherosclerosis	Poshen B. Chen et al	2020	LDLr−/− mice	The study devised an invitroscreening protocol of the mouse gut microbiome to discover molecules that can selectively modify bacterial growth. This approach was used to identify cyclic D,L-α-peptides that remodeled the WD gut microbiome toward the low fat diet microbiome state.	Directed chemical manipulation provides an additional tool to decipher the chemical biology of the gut microbiome and may advance microbiome-targeted therapeutics.
Fecal microbiota transplantation ameliorates atherosclerosis in mice with C1q/TNF-related protein 9 genetic deficiency	Eun Sil Kim et al	2022	C57BL/6 mice CTRP9-KO mice	This study was performed to determine the impact of the gut microbiota on the pathogenesis of atherosclerosis caused by genetic deficiency.	This study showed that mutations in the genetic background can alter the composition of the gut microbiome and result in atherosclerosis. In addition, FMT using healthy donor stool can protect against this disease in CTRP9-deficient mice.
Therapeutic potential of human umbilical cord mesenchymal stem cells on aortic atherosclerotic plaque in a high-fat diet rabbit model	Yanhong Li et al	2021	Japanese big-ear white rabbits<br>Umbilical cord stem cells	The study aimed to investigate the effect of UCSCs treatment on atherosclerotic plaque formation and the progression of lesions in a high-fat diet rabbit model.	This study demonstrates that UCSCs transplantation in the treatment of AS at the early stages can not only attenuate atherosclerotic plaque formation and progression in large and medium-sized vessels, but also improve early peripheral blood filling. UCSCs transplantation can alleviate AS by reducing the serum lipid level, inhibiting the production of macrophages and inflammatory cytokines (IL-6 and TNF-α), inhibiting apoptosis, promoting production of anti-inflammatory cytokines (IL-10 and TGFβ) and endothelial cells, further inhibiting inflammatory responses and repairing damaged endothelium. UCSCs can also inhibit inflammation progression and ox-LDL phagocytosis by balancing intestinal flora dysbiosis caused by high-fat diet, as well as reducing TMAO production, which can also

					lessen atherosclerotic plaque burden. Future work will provide and in-depth investigation of the molecular mechanism by which UCSCs regulate intestinal flora and metabolite production to reduce AS progression.
The role of gut microbiome and its interaction with arsenic exposure in carotid intima-media thickness in a Bangladesh population	Fen Wu et al	2019	Human	This study aim was to examine the inter-relationships between arsenic exposure, the gut microbiome, and carotid IMT—a surrogate marker for atherosclerosis.	This study suggest a role of Citrobacter in the development of atherosclerosis, especially among individuals with higher levels of arsenic exposure.
Chronic Intermittent Hypoxia Participates in the Pathogenesis of Atherosclerosis and Perturbs the Formation of Intestinal Microbiota	Chaowei Hu et al	2021	ApoE-/- mice	The study aimed to reveal the shifts of GM in response to CIH induction during the development of AS and provide further evidence to develop preventive strategies for atherosclerotic cardiovascular risk reduction in patients suffering from OSA.	The study demonstrated a causal effect of CIH on GM alterations in AS mice and suggested that the disordered GM features in AS development were deteriorated by CIH, which may be associated with AS aggravation. Preventative strategies targeting gut microbiome are highly recommended for intervention of OSA-related AS.
Propamocarb exposure has the potential to accelerate the formation of atherosclerosis in both WT and ApoE−/− mice accompanied by gut microbiota dysbiosis	Cuiyuan Jin et al	2021	C57BL/6 J WT mice ApoE−/− mice	The effect of propamocarb on the formation of atherosclerosis was evaluated in WT and ApoE−/−mice.	In summary, propamocarb exposure induced initial symptoms of atherosclerosis in WT mice under different dietary conditions and exacerbated atherosclerosis development in ApoE−/− mice. Atherosclerosis caused by propamocarb exposure may result from lipid metabolism disorders, inflammation and gut microbiota dysbiosis. This study confirmed the risk of propamocarb for the occurrence of cardiovascular disease, which should not be

					ignored.
Diesel exhaust particles alter the profile and function of the gut microbiota upon subchronic oral administration in mice	Sybille van den Brule et al	2021	ApoE−/− mice C57Bl/6JRj mice	This study explore the possible contribution of the GM to mediate the CM toxicity of inhaled PM. This study tested this hypothesis by subchronically administering DEP by gavage, at doses corresponding to mucociliary clearance from inhalation exposure.	This study show here that oral exposure to DEP, at doses relevant for human health, changes the composition and function of the gut microbiota. These modifications were, however, not translated into ultimate atherosclerotic or metabolic outcomes.
Clock-Bmal1 mediates MMP9 induction in acrolein-promoted atherosclerosis associated with gut microbiota regulation	Xiaoyue Wu et al	2019	Huamn<br>Human umbilical vein endothelial cells<br>ApoE-/-mice	The study was aimed to explore the roles of circadian clock and intestinal flora in acrolein-accelerated atherogenesis in vivo and in vitro offering new thesis into mechanisms of atherosclerosis.	This study indicated that acrolein increased the expression of MMP9 through MAPK regulating circadian clock, which was associated with gut microbiota regulation in atherosclerosis. Circadian rhythms and gut microbiota might be promising targets in the prevention of cardiovascular disease caused by environmental pollutants.
CRISPR/Cas9 based blockade of IL-10 signaling impairs lipid and tissue homeostasis to accelerate atherosclerosis	Haozhe Shi et al	2022	Syrian golden hamsters<br>IL-10 mutant Syrian golden hamster	Given that Syrian golden hamsters possess metabolic features similar to humans , this study generated an IL-10deficient hamster model using CRISPR/Cas9 editing to investigate the role of IL-10 in lipid metabolism and atherosclerosis.	In conclusion, this study report an IL-10 mutant hamster model and characterize the phenotypes caused by IL-10 deficiency. This study clearly demonstrate a role for IL-10 in maintaining lipid and tissue homeostasis, and in preventing atherosclerotic lesion development. This study confirm the causal relationship between IL-10 and atherosclerosis and providing new insight into IL-10 as a potential therapeutic for the prevention or treatment of atherosclerosis.

Influence of Intermittent Hypoxia/ Hypercapnia on Atherosclerosis, Gut Microbiome, and Metabolome	Jin Xue et al	2021	ApoE−/− mice	This study hypothesized that IH or IC induces specific alterations in the gut microbiome and their metabolites, which may promote atherosclerosis. The study sought to address in the current study were (1) what is the particular role of IH or IC in inducing or promoting atherosclerosis, (2) what is the response of the vascular system (i.e., aorta vs. pulmonary artery, PA) to IH or IC in term of atherogenesis, (3) what is the signature of IH or IC on gut microbiome and metabolites, and (4) what is the potential impact of these changes of gut microbiome and metabolites on the development of atherosclerosis?	This study is the first to show that IC, in addition to IH, contributes to OSA-related atherosclerosis. Moreover, the study also demonstrate that IHC, a hallmark of OSA, change the gut microbiota and metabolites. The changes in the gut luminal environment likely influence the development of atherosclerosis by modulating host gut permeability, inflammatory responses, microbial metabolites TMA/TMAO, and bile acid and lipid metabolism. The knowledge obtained in the current study paves the way for a better understanding of the mechanistic link between IH/IC, gut microbiome, and OSA-induced atherosclerosis.

AS: atherosclerosis, ApoE-/- mice: Apolipoprotein E knockout mice, Ldlr−/− mice: Low-density lipoprotein receptor knockout mice, CAC: Coronary artery calcification, CAD:Coronary artery disease ,GA:gallic acid, TMAO:trimethylamine-N-oxide,TMA:trimethylamine ,HFD:high fat-diet, SA:sialic acid,3′-SL:3′-Sialyllactose , CCPS:Sulfated polysaccharides ,F&V:fruit and vegetable,RYR:red yeast rice, DP:dual-protein , HF:high fat, WD:Western high fat diet, FO:Flaxseed oil, BCAA: Branched-Chain Amino Acids ,MLM:medium-, long-, and medium-chain ,ESM:egg sphingomyelin, CSDRW:Cabernet Sauvignon dry red wine,HCD:high-choline western diet, ASTE:astaxanthin-rich extract,
ATO:atorvastatin, LRPA:L. ruthenicum, FMB:foxtail millet bran, I3C:Indole-3-carbinol, HAL:Helianthus Annuus L, QA:Quinic acid, MSPs:Millet shell polyphenols, PSE:peanut skin extract, PCA:protocatechuic acid, BBR:berberine, ASBUE:Acanthopanax senticosus, GS:guggulsterone, HPLC:high-performance liquid chromatography, MS:mass spectroscopy, LC:liquid chromatography, PMRP:Polygoni Multiflori Radix Praeparata, RM:red meat, UEE:Usnea ethanol extract, BBR:berberine, GEO:ginger essential oil, GAN:Gubra Amylin NASH, LPS:lipopolysaccharide, VSMC:vascular smooth muscle cell, BXD；Banxia Xiexin decoction, BHD:Buyang Huanwu decoction, T2DM:type 2 diabetes mellitus, BIC:bicyclol, UAP:unstable angina pectoris, WT:wild-type, Abx:antibiotic, HFHC:high fat and high-cholesterol, HC:high-cholesterol, IMT:intima-media thickness, WT:wild-type, NF-κB:Nuclear Factor-kappa B.

Supplementary Table 6:The underlying mechanisms through which GM modulate the progression of AS.

Title	Gut Microbiota Composition Analysis	Changes in inflammation	Regulating lipid metabolism	Intestinal mucosal barrier
Candida albicans accelerates atherosclerosis by activating intestinal hypoxia-inducible factor2a signaling	Increased abundance of *Alistipes senegalensis*.	Elevated levels of HIF-2a and IL-1b.	Heightened cholesterol and TG in the liver. Enhanced plasma levels of cholesterol, TG, VLDL-C, and LDL-C.	
Low-grade endotoxaemia enhances artery thrombus growth via Toll-like receptor 4: implication for myocardial infarction		Elevated plasma sP-selectin levels.		
Both gut microbiota and cytokines act to atherosclerosis in ApoE-/- mice	Increased abundance of *Firmicutes* and *Deferribacteres*. Decreased abundance of *Bacteroidetes* and *Actinobacteria*.	Heightened plasma levels of IFN-γ, IL-6, and MCP-1.	Elevated serum levels of TG, TCho, HDL-C and LDL-C.	
A Proinflammatory Gut Microbiota Increases Systemic Inflammation and Accelerates Atherosclerosis	Decreased abundance of *Akkermansia*, *Christensenellaceae, Clostridium*, and *Odoribacter*.	Increased plasma levels of IL-1β, IL-2, IL-10, and IFN-γ. Enhanced counts of Ly6Clo and Ly6Chi monocytes and neutrophils in the blood.		

Chronic apical periodontitis exacerbates atherosclerosis in apolipoprotein E-deficient mice and leads to changes in the diversity of gut microbiota	Increased abundance of *Firmicutes, Chloroflexi, Cyanobacteria, Allobaculum*, and *Sutterella*.<br>Decreased abundance of *Lactobacillus, Helicobacter,* and *Bacteroidetes*.		Elevated LDL-C levels.<br>Decreased HDL-C levels.	
Unveiling the oral-gut connection: chronic apical periodontitis accelerates atherosclerosis via gut microbiota dysbiosis and altered metabolites in apoE−/− Mice on a high-fat diet	Decreased α-diversity of gut microbiota.<br>Increased abundance of *Erysipelotrichaceae* and *Odoribacter*.<br>Decreased abundance of *Faecalibacterium* and *Lachnospiraceae*.		Regulated lipid metabolism and primary bile acid synthesis, promoting increased levels of TCDCA, TCA, and TDCA.	Decreased expression of tight junction proteins Zo-1, claudin, and occluding.<br>Enhanced gut permeability.
Gut microbiota may mediate the impact of chronic apical periodontitis on atherosclerosis in apolipoprotein E-deficient mice	Increased abundance of *Ruminococcaceae, Lachnospiraceae, Paraprevotellaceae, Prevotellaceae, Porphyromonadaceae, Moraxellaceae, Oxalobacteraceae, Anaeroplasmataceae, Enterobacteriaceae, Prevotella, p_75_a5, Oscillospira, AF12, Ruminococcus_*, *Ralstonia, Coprococcus, Clostridium, Anaeroplasma, Serratia*.			

Helicobacter pylori Infection Acts Synergistically with a High-Fat Diet in the Development of a Proinflammatory and Potentially Proatherogenic Endothelial Cell Environment in an Experimental Model		Elevated serum levels of CRP.<br>Helicobacter pylori components (GE and LPS) inducing macrophages to transform significantly into foam cells.<br>GE component dose-dependently upregulating apoptosis in endothelial cells.<br>Increased Bax protein expression.	
Oral *Porphyromonas gingivalis* infection affects intestinal microbiota and promotes atherosclerosis	Increased abundance of *Mucispirillum schaedleri* and *Lactobacillus gasseri*.		Increased serum TG levels.<br>Increased LDL/HDL ratio.<br>Upregulated ABCA1 expression in liver tissue.<br>Downregulated SR-B1 expression in the liver.
Bacteroides fragilis Supplementation Deteriorated Metabolic Dysfunction, Inflammation, and Aorta Atherosclerosis by Inducing Gut Microbiota Dysbiosis in Animal Model	Increased abundance of *Desulfovibrionaceae*.<br>Decreased abundance of *Bacteroidaceae* and *Lactobacillaceae*.	Upregulated mRNA expression levels of CD36, F4/80, TLR2, and TLR4 in duodenal tissue.<br>Upregulated mRNA expression levels of CD36 and F4/80 in aortic tissue.	Elevated serum TC and LDL-C levels.
The Microbiota Promotes Arterial Thrombosis in Low-Density Lipoprotein Receptor-Deficient Mice	Increased abundance of *Clostridiaceae, Staphylococcaceae, Bacillales, Streptococcaceae*, and *Clostridiales*.<br>Decreased abundance of *Lactobacillaceae, Proteobacteria,* and *Betaproteobacteria*.<br>Increased *F/B* ratio.	Increased counts of monocytes and neutrophils in plasma.<br>Elevated levels of monocyte-derived pro-inflammatory chemokines CCL7 and CXCL1.<br>Decreased levels of T cell-related cytokines IL-9 and IL-27.	

The Role of Intestinal Dysbacteriosis Induced Arachidonic Acid Metabolism Disorder in Inflammaging in Atherosclerosis	Increased abundance of *Lachnospiraceae_FCS020, Ruminococcaceae_UCG-009, Acetatifactor, Lachnoclostridium, Lactobacillus gasseri, Desulfovibrio.* Decreased abundance of *Ruminococcaceae-UCG-014* and *Bacteroides*. Increased *F/B* ratio. Elevated LPS levels.	Increased serum GM-CSF, IFN-g, IL-7, TNF-a, IL-1b, IL-12, MCP-1, CD40L, KCIL8, IL-13 levels. Decreased serum IL-10 levels.	Promoted metabolism of AA. Elevated levels of AA metabolites LTB4, PGF2a, and 20-HETE.	Decreased expression of tight junction proteins Zo-1 and occluding.
Experimental Periodontitis Deteriorated Atherosclerosis Associated With Trimethylamine N-Oxide Metabolism in Mice	Increased abundance of *Lachnospiraceae_NK4A136_group, Acetatifactor, Lachnospiraceae_bacterium_A4,Lactobacillus_animalis,Parabacteroides_goldsteinii,Lachnospiraceae_NK4A136_group,Mucispirillum, Ruminococcaceae_UCG-01.* Elevated LPS levels.	Increased serum inflammatory cytokines IL-6 and TNF-a.		Downregulated mRNA expression of tight junction proteins Zo-1, Claudin-1, and Occludin.
Desulfovibrio desulfuricans aggravates atherosclerosis by enhancing intestinal permeability and endothelial TLR4/NF-kB pathway in Apoe-/-mice	Decreased abundance of *Akkermansia muciniphila, prausnitzii, Roseburia intestinalis*. Elevated LPS levels.	Increased serum pro-inflammatory cytokines IL-1b, IL-6, and TNF-a. Enhanced cytokine levels in the aorta including IL-1b, IL-6, and TNF-a. Upregulated mRNA expression of F4/80, MCP-1, ICAM-1, and VCAM-1 in the aorta. Increased levels of chemokines ICAM-1 and MCP-1, promoting LPS-induced TLR4/NF-κB signaling with increased expression of TLR4 and P-p65.		Increased gut permeability. Decreased mucus layer thickness and the number of goblet cells. Decreased expression of tight junction proteins Zo-1,Occludin,and Claudin-3.

HIF-2α: Hypoxia-Inducible Factor 2 Alpha, IL-1β: Interleukin 1 Beta, IFN-γ: Interferon Gamma, IL-6: Interleukin 6, MCP-1: Monocyte Chemoattractant Protein-1, IL-1β: Interleukin 1 Beta, IL-2: Interleukin 2, IL-10: Interleukin 10, Ly6Clo: Lymphocyte Antigen 6 Complex, Locus C Low, Ly6Chi: Lymphocyte Antigen 6 Complex, Locus C High, CRP: C-Reactive Protein, Bax: BCL2-Associated X Protein, GE: glycine extract, LPS: Lipopolysaccharide, CD36: Cluster of Differentiation 36,F4/80: EGF-like Module-Containing Mucin-Like Hormone Receptor-Like 1, TLR2: Toll-Like Receptor 2, TLR4: Toll-Like Receptor 4, CCL7: Chemokine (C-C Motif) Ligand 7, CXCL1: Chemokine (C-X-C Motif) Ligand 1, IL-9: Interleukin 9, IL-27: Interleukin 27, IFN-g: Interferon Gamma, CD40L: CD40 Ligand, KCIL8: Keratinocyte Chemoattractant Interleukin 8, IL-13: Interleukin 13, ICAM-1: Intercellular Adhesion Molecule 1, VCAM-1: Vascular Cell Adhesion Molecule 1, NF-κB: Nuclear Factor Kappa-Light-Chain-Enhancer of Activated B, P-p65: Phosphorylated p65, TG: Triglycerides, VLDL-C: Very Low-Density Lipoprotein Cholesterol, LDL-C: Low-Density Lipoprotein Cholesterol, TCho: Total Cholesterol, HDL-C: High-Density Lipoprotein Cholesterol, TCDCA: Taurochenodeoxycholic Acid, TCA: Taurocholic Acid, TDCA: Taurodeoxycholic Acid, ABCA1: ATP Binding Cassette Subfamily A Member 1, SR-B1: Scavenger Receptor Class B Member 1, AA: Arachidonic Acid, LTB4: Leukotriene B4, PGF2α: Prostaglandin F2 Alpha, 20-HETE: 20-Hydroxyeicosatetraenoic Acid, Zo-1: Zonula Occludens-1,F/B: *Firmicutes/Bacteroidetes*.

Supplementary Table 7: This table illustrates the principal mechanisms of the GMM's impact on AS, as examined in this study.

Title	Gut Microbiota Composition Analysis	Changes in inflammation	Regulating lipid metabolism	Intestinal Mucosal Barrier
Integrated metagenomics identifies a crucial role for trimethylamine-producing Lachnoclostridium in promoting atherosclerosis	Increased abundance of *Lachnoclostridium, Clostridium*, and *Olsenella* in AS patients.	L. saccharolyticum promotes the expression of IL-1β, TNF-α, ICAM-1, VCAM-1, MCP-1, Cd68, and F4/80 genes.		
l-Carnitine in omnivorous diets induces an atherogenic gut microbial pathway in humans				
Plasma trimethylamine N-oxide (TMAO) levels predict future risk of coronary artery disease in apparently healthy individuals in the EPIC-Norfolk Prospective Population Study				
The Association between Trimethylamine N-Oxide and Its Predecessors Choline, L-Carnitine, and Betaine with Coronary Artery Disease and Artery Stenosis				
Choline and trimethylamine N-oxide supplementation in normal chow diet and western diet promotes the development of atherosclerosis in Apoe –/– mice through different mechanisms	Choline or TMAO decreased the relative abundance of *Bifidobacterium, Clostridium*, and *Lactobacillus*.<br>Choline reduced the relative abundance of *Streptococcus*.<br>Choline and TMAO treatment increased the relative abundance of *Desulfovibrio*.<br>Choline or TMAO promoted the expression of ICAM-1 and VCAM-1.	Western diet supplementation with choline or TMAO increased TNF-α, IL-1β, and MCP-1 levels.	Choline or TMAO elevated serum LDL-C, HDL-C, and TC levels.	

Choline Supplementation Does Not Promote Atherosclerosis in CETP-Expressing Male Apolipoprotein E Knockout Mice	No influence.	Not affected blood lipid levels.
Dietary Choline or Trimethylamine N-oxide Supplementation Does Not Influence Atherosclerosis Development in Ldlr−/− and Apoe−/− Male Mice		
Inhibition of Trimethylamine N-oxide attenuates neointimal formation through reduction of inflammasome and oxidative stress in a mouse model of carotid artery ligation	Upregulated expression of NLRP3 inflammasome and ASC.<br>Increased levels of pro-inflammatory cytokines such as IL-1β, IL-6, and TNF-α.<br>Enhanced protein expression of ICAM-1 and p-NF-κB p65/NF-κBp65 levels.<br>Upregulated expression of Bax.<br>Increased expression of PERK and IRE-1α in arterial tissue.<br>Promotion of ERK and JNK phosphorylation.<br>Elevated ROS levels.<br>Upregulated expression of NLRP3, ASC, and caspase<br>Activation of phosphorylated NF-κB p65.	
Plasma Levels of TMAO can be Increased with ‘Healthy’ and ‘Unhealthy’ Diets and Do Not Correlate with the Extent of Atherosclerosis but with Plaque Instability	Increased levels of circulating monocytes, neutrophils, and P-selectin.<br>Increased abundance of Enterorhabdus and Lachnoclostridium.	Not affected blood lipid levels.

Gut-Flora-Dependent Metabolite Trimethylamine-N-Oxide Promotes Atherosclerosis-Associated Inflammation Responses by Indirect ROS Stimulation and Signaling Involving AMPK and SIRT1.	Enhanced species diversity of the gut microbiota.<br>Increased relative abundance of *Bacteroidetes* and *Firmicutes*.<br>Increased *F/B* ratio.	Downregulated expression of SIRT1.<br>Promotion of ROS generation.<br>Elevated plasma levels of IL-1β, IL-6, TNF-α, NF-κB, MMP9, and NLRP3 inflammatory cytokines.	Increased plasma TC, HDL-C, and LDL-C levels.	
Fecal level of butyric acid, a microbiome- derived metabolite, is increased in patients with severe carotid atherosclerosis.	Increased abundance of *Roseburia* and *Faecalibacterium*.	No significant correlation.		No significant correlation.
Butyrate protects endothelial function through PPARδ/miR-181b signaling		Upregulated expression of PPARδ and miR-181b.<br>Elevated IL-1β levels.<br>Reduced endothelial ROS.		
Choline and butyrate beneficially modulate the gut microbiome without affecting atherosclerosis in APOE*3-Leiden.CETP mice	Increased abundance of anti-inflammatory bacteria such as *Duncaniella spB8, Blautia* producta, and *Faecalibaculum prausnitzii*.		Not affected blood lipid levels.	
Propionate attenuates atherosclerosis by immune-dependent regulation of intestinal cholesterol metabolism		Increased regulatory T cells and interleukin-10 in the small intestine.	Decreased plasma TC, VLDL, and LDL levels.<br>Upregulated expression of cholesterol-regulating gene Srebp.<br>Downregulated expression of Cyp7a.<br>Decreased gene expression of Npc1l1 in the small intestine.	
Butyrate suppresses atherosclerotic inflammation by regulating macrophages and polarization via GPR43/HDAC-miRNAs axis in ApoE−/− mice	Decreased *F/B* ratio.<br>Decreased abundance of *Firmicutes*.<br>Increased abundance of *Bacteroidetes, Verrucomicrobiota*, and *Faecalibaculum*.	Decreased plasma levels of pro-inflammatory cytokines IL-1β, IL-6, IL-17A, and IFN-γ.<br>Elevated anti-inflammatory IL-10 levels.<br>Downregulated mRNA expression of TNF-α, IL-1β, IL-6, IL-17A, and	Decreased plasma TC, TG, and LDL-C levels.	Upregulated expression of tight junction protein ZO-1 in the intestinal barrier.

		IFN-γ in atherosclerotic plaques.<br>Inhibition of macrophage M1 polarization.<br>Enhanced M2 polarization.	
Gut Microbially Produced Indole-3-Propionic Acid Inhibits Atherosclerosis by Promoting Reverse Cholesterol Transport and Its Deficiency Is Causally Related to Atherosclerotic Cardiovascular Disease	Decreased abundance of *Bacteroides vulgatus, Clostridium sporogenes*, and *Peptostreptococcus russellii* in CAD patients.<br>Increased abundance of *Fusobacterium varium, Citrobacter rodentium*, and *Ruminococcus gnavus* in CAD patients.<br>Decreased abundance of key IPA-producing microbes *Clostridium* and *Peptostreptococcus* in CAD patients.		Reduced plasma total cholesterol and triglyceride levels.<br>Downregulated SPI1 expression.<br>Decreased miR-142-5p levels.<br>Upregulated ABCA1 expression in macrophages.<br>Reduced foam cells.
Tryptophan metabolism, gut microbiota, and carotid artery plaque in women with and without HIV infection	Increased abundance of *Roseburia, Lachnospira, Barnesiellaceae_unclassified, Clostridiales_unclassified*, and *RF39_unclassified*.<br>Decreased abundance of *Eggerthella*.		
Gut microbiome-derived glycine lipids are diet-dependent modulators of hepatic injury and atherosclerosis		Downregulated expression of Major Histocompatibility Complex (MHC) class II-related genes (H2αα, H2ab1, H2eb1, H2dma, H2dmb1, Cd74, Irf8).<br>Decreased expression of inflammatory marker genes such as IL-1β.<br>Downregulated expression of IFNγ-induced H2aa, H2eb1, and IL-1β.	Reduced serum TC and non-HDL-C, NEFAs levels.<br>Decreased liver cholesterol.

Glycoursodeoxycholic Acid Ameliorates Atherosclerosis and Alters Gut Microbiota in Apolipoprotein E–Deficient Mice	Increased abundance of *Alloprevotella* and *Parabacteroides*.<br>Decreased abundance of *Turicibacter* and *Alistipes*.	Inhibition of macrophage recruitment.<br>Downregulated mRNA levels of MCP-1 and IL-1β.<br>Downregulated mRNA level of SR-A.	Decreased liver levels of TG, TC, and LDL-C.<br>Decreased serum levels of TG, TC, and LDL-C.<br>Downregulated expression of acyl-CoA cholesteryl acyltransferases<br>Promotion of fecal cholesterol excretion.
A novel candidate for prevention and treatment of atherosclerosis: Urolithin B decreases lipid plaque deposition in apoE-/- mice and increases early stages of reverse cholesterol transport in ox-LDL treated macrophages cells			Promotion of cholesterol excretion.<br>Increased expression of SR-BI and ABCA1 proteins in macrophages.
Urolithin A promotes atherosclerotic plaque stability by limiting inflammation and hypercholesteremia in Apolipoprotein E–deficient mice		Decreased expression of VCAM-1 in the aorta.<br>Decreased mRNA levels of Yap and Cyr61 in the aorta.<br>Increased mRNA level of eNOS.	Decreased mRNA expression of Scd1, Srebp1, and Srebp2 in the liver tissue.<br>Increased expression of Fxr and Lxrα in the liver tissue.<br>Decreased levels of mature SREBP1 and SREBP.

IL-1β: Interleukin 1β,TNF-α: Tumor Necrosis Factor α, ICAM-1: Intercellular Adhesion Molecule 1, VCAM-1: Vascular Cell Adhesion Molecule 1 , MCP-1: Monocyte Chemoattractant Protein-1, Cd68: Cluster of Differentiation 68, F4/80: EGF-like Module-Containing Mucin-Like Hormone Receptor-Like 1, TMAO: Trimethylamine N-Oxide, NLRP3: NLR Family Pyrin Domain Containing 3, ASC: Apoptosis-Associated Speck-like Protein Containing a CARD, MMP9: Matrix Metallopeptidase 9, SIRT: Sirtuin1, *F/B: Firmicutes to Bacteroidetes* Ratio, ROS: Reactive Oxygen Species, NF-κB: Nuclear Factor Kappa-Light-Chain-Enhancer of Activated B Cells, IL-6: Interleukin 6, miR-181b: MicroRNA-181b, IFN-γ: Interferon Gamma, IL-17A: Interleukin 17A, H2dma: Histocompatibility 2, Class II, Locus DMa H2dmb1: Histocompatibility 2, Class II, Locus DMb1, Cd74: Cluster of Differentiation 74, Irf8: Interferon Regulatory Factor 8, SR-A: Scavenger Receptor Class A, eNOS: Endothelial Nitric Oxide Synthase, Cyr61: Cysteine-Rich Angiogenic Inducer 61, Yap: Yes-Associated Protein, LDL-C: Low-Density Lipoprotein Cholesterol, HDL-C: High-Density Lipoprotein Cholesterol, TC: Total Cholesterol, Cyp7a: Cytochrome P450 Family 7 Subfamily A Member 1, VLDL: Very Low-Density Lipoprotein, LDL: Low-Density Lipoprotein, Npc1l1: Niemann-Pick C1-Like 1, miR-142-5p: MicroRNA-142-5p, SPI1: Spi-1 Proto-Oncogene, SR-BI: Scavenger Receptor Class B Type I, ABCA1: ATP Binding Cassette Subfamily A Member 1, Fxr: Farnesoid X Receptor, Lxrα: Liver X Receptor Alpha, SREBP1: Sterol Regulatory Element-Binding Protein 1, SREBP: Sterol Regulatory Element-Binding Protein, non-HDL-C: Non-High-Density Lipoprotein Cholesterol, NEFAs: Non-Esterified Fatty Acids, ZO-1: Zonula Occludens-1.

Supplementary Table 8:This table presents the main mechanisms by which various intervention factors influence the occurrence and development of AS.

Title	Gut Microbiota Composition Analysis	Changes in inflammation	Regulating lipid metabolism	Intestinal Mucosal Barrier
A consortium of three-bacteria isolated from human feces inhibits formation of atherosclerotic deposits and lowers lipid levels in a mouse model	Increased the abundance of *Bacteroides cellulosilyticus, Faecalibacterium prausnitzi*i, and *Roseburia intestinalis*.		*F. prausnitzii, B. cellulosilyticus,* and *R. intestinalis* reduced serum TG and LDL-C levels.<br>*F. prausnitzii, B. cellulosilyticus*, and *R. intestinalis* upregulated the expression of nuclear bile acid receptor farnesoid X receptor (FXR; NR1H4) and NR4A1 (Nur77).<br>Lowered plasma long-chain fatty acids.<br>Elevated plasma lithocholic acid levels.	
Isolation and characterization of a novel choline degrading Citrobacter amalonaticus strain from the human gut				
Metagenomic analysis of the gut microbiome in atherosclerosis patients identify cross-cohort microbial signatures and potential therapeutic target			*E. eligens* promoted CDP-diacylglycerol biosynthesis.	
Faecalibacterium prausnitzii as a potential Antiatherosclerotic microbe		*F. prausnitzii* decreased the levels of inflammatory factors such as F4/80, MCP1, VCAM1, CD4, CD86, and Foxp3.		Upregulated the expression of ileal tight junction protein ZO-1.
Bifidobacterium animalis subsp. lactis F1−7 Alleviates Lipid Accumulation in Atherosclerotic Mice via Modulating Bile Acid Metabolites to Downregulate Intestinal FXR	Increased the relative abundance of *Desulfobacterota, Actinobacteria, Verrucomicrobiota, Bifidobacterium, Lactobacillus, Faecalibaculum,* and *Desulfovibrio.*<br>Reduced the relative abundance of *Firmicutes, Bacteroidetes, Norank_f_Muribaculaceae, Dubosiella, Clostridium_sensu_stricto_1, Lachnospiraceae_NK4A136_group*, and *Turicibacter*.<br>Lowered the *F/B* ratio.	Reduced plaque area inflammatory cells and foam cells.	Lowered serum TG, TC, and LDL levels.<br>Elevated serum HDL-C levels.<br>Downregulated FXR expression.<br>Reduced FGF15 levels.<br>Decreased CYP7A1 protein levels.	

Enterobacter aerogenes ZDY01 inhibits cholineinduced atherosclerosis through CDCA-FXR-FGF15 axis	Increased the abundance of *Turicibacter* and *unidentified_Ruminococcaceae*.	Reduced macrophage content in plaques.	Lowered plasma TG and LDL-C levels.<br>Elevated plasma HDL-C levels.<br>Upregulated mRNA expression of Cyp7a1, Abcg5, and Abcg8.<br>Reduced cecal CDCA levels.<br>Downregulated the FXR/FGF15 pathway.<br>Upregulated Cyp7a1 expression.<br>Promoted RCT.
Anti-atherosclerotic effects of *Lactobacillus plantarum ATCC 14917* in ApoE−/− mice through modulation of proinflammatory cytokines and oxidative stress	Increased the abundance of *Bacteroidetes*.<br>Reduced the abundance of *Firmicutes, Verrucomicrobia,* and *Proteobacteria*.<br>Lowered the *F/B* ratio.	Reduced serum oxLDL and MDA levels.<br>Increased serum SOD levels.<br>Lowered serum TNF-α and IL-1β levels.<br>Downregulated aortic TNF-α mRNA and IL-1β mRNA expression.	
Lactic acid bacteria prevent both periodontitis and atherosclerosis exacerbated by periodontitis in spontaneously hyperlipidemic mice	Increased the abundance of lactic acid bacteria.<br>Reduced the abundance of *Bacteroides*.	Downregulated IL-6 and TNF-α gene expression.<br>Lowered serum IL-1β and TNF-α levels.	
Krill Oil Combined with *Bifidobacterium animalis subsp. lactis F1-7* Alleviates the Atherosclerosis of ApoE−/− Mice		Lowered serum IL-1β and TNF-α levels.<br>Downregulated MYD88 and NFκB expression.	Reduced serum TC, TG, and LDL-C levels.<br>Elevated HDL-C levels.<br>Downregulated intestinal FXR expression.<br>Improved liver CYP7A1 expression.<br>Promoted bile acid metabolism.
Metabolite acetyl-L-carnitine participates in *Bifidobacterium animalis F1-7* to ameliorate atherosclerotic inflammation by downregulating theTLR4/NF-κB pathway		Downregulated aortic MyD88 and NF-κB p65 expression.<br>Lowered serum TNF-α and IL-1β levels.<br>Downregulated gut TLR4, MyD88, and NF-κB p65 gene expression.	ALC significantly reduced cellular uptake of ox-LDL, decreasing foam cell formation.

Reduction of intestinal trimethylamine by probiotics ameliorated lipid metabolic disorders associated with atherosclerosis			Lowered serum TC and TG levels.<br>Downregulated FXR expression.<br>Upregulated liver CYP7A1 expression.<br>Reduced hepatic lipid accumulation.	
Lactobacillus rhamnosus GG protects against atherosclerosis by improving ketone body synthesis	Increased the Shannon index and Simpson index.<br>Reduced the relative abundance of *Proteobacteria* and *Desulfovibrionaceae*.<br>Increased the relative abundance of *Firmicutes* and *Lactobacillus*.			
Chitin oligosaccharides alleviate atherosclerosis progress in ApoE-/mice by regulating lipid metabolism and inhibiting inflammation	Reduced serum LPS levels.	Lowered aortic levels of inflammatory cytokines such as IL-6, IL-1β, TNF-α, and MCP-1.<br>Downregulated protein expression of monocyte chemoattractant factors: MMP-2 and VCAM-1.<br>Decreased serum levels of IL-1β, IL-6, TNF-α, and MCP-1.	Reduced serum TC, TG, LDL-C, VLDL, and ox-LDL levels.<br>Lowered hepatic TG and TC content.<br>Alleviated liver cell degeneration.<br>Downregulated Hmgcr gene expression.<br>Increased colonic SCFAs content.<br>Upregulated hepatic expression of Ldlr and Sr-b1 genes.<br>Increased protein expression of Ldlr and Sr-b1.<br>Upregulated expression of Cyp7a1, Abcg5, and Abcg8 genes.<br>Downregulated intestinal gene expression of Niemann-Pick C1-like 1 (Npc1l1).<br>Upregulated intestinal expression of Abcg5 and Abcg8.	Upregulated mRNA levels of tight junction proteins Zona occludins 1 (Zo-1) and Occludin.
Dissecting the impact of dietary fiber type on atherosclerosis in mice colonized with different gut microbial communities	Decreased *F/B* ratio.<br>Increased abundance of butyrate-producing bacteria such as *Clostridium, Oscillospira, Ruminococcus, Gemmiger,* and *Faecalibacterium*.		Enhanced total SCFA concentration.	
Neoagarotetraose Alleviates Atherosclerosis via Modulating Cholesterol and Bile Acid Metabolism in ApoE−/− Mice	Increased abundance of *Bacteroidota, Campilobacterota, no-rank f_Muribaculaceae, g_unclassified f_Lachnospiraceae,* and *Blautia*.<br>Decreased abundance of *Actinobacteriota, Faecalibaculum, Desulfovibrio*.		Reduced serum TC, TG, and LDL-C levels.<br>Increased serum and hepatic HDL-C levels.<br>Alleviated hepatic steatosis.<br>Upregulated hepatic mRNA expression levels.<br>Increased liver protein expression of	

			Srbi, Abca1, HMGCR, SR-BI, LXR, ABCG1, and ABCG5.<br>Upregulated mRNA expression levels of Npc1l1 and Abcg8.<br>Increased intestinal ABCG8 protein expression.	
Manno-oligosaccharides from Cassia Seed Gum Attenuate Atherosclerosis through Inflammation Modulation and Intestinal Barrier Integrity Improvement in ApoE−/− Mice	Decreased *F/B* ratio.<br>Reduced abundance of *Actinobacteria, Verrucomicrobiota*.<br>Increased abundance of *Helicobacter, norank_f_Muribaculaceae, unclassified_f_Lachnospiraceae, Colidextrebacter*.	Reduced serum levels of TNF-α, IL-6, and NO.<br>Downregulated relative mRNA expression levels of ICAM-1, VCAM-1, MCP-1, TNF-α, Il-6, and iNOS in the aortic tissue.<br>Downregulated mRNA expression levels of TNF-α and INOS in the colonic tissue.<br>Decreased ICAM-1 expression.	Lowered serum TC, TG, LDL-C, and ox-LDL levels.<br>Reduced hepatic TC, TG, and LDL-C levels.	Upregulated mRNA expression of Occludin, Zo-1, and Claudin-1.
Potential effects of sialic acid and 3′-Sialyllactose on intestinal health and anti-cardiovascular disease in mice fed with a high-fat diet	Increased abundance of *Verrucomicrobiota, Bacteroidota, Akkermansia, Blautia, Lactobacillus, Faecalitalea, Parasutterella*.<br>Decreased abundance of *Firmicutes, Desulfobacterota, Proteobacteria, Actinobacteriota, Acidobacteriota, Planctomycetota, Chloroflexi, Myxococcota, Desulfovibrio, Erysipelatoclostridium, Faecalibaculum*.	Reduced serum IL-6, TNF-α levels.	Decreased serum TG and LDL-C levels.<br>Increased serum HDL-C level.	
Protective Activities of Polysaccharides from Cipangopaludina chinensis Against High-Fat Diet Induced Atherosclerosis via Regulating Gut Microbiota in ApoE-deficient Mice	Decreased *Firmicutes* abundance.<br>Increased abundance of *Bacteroidetes, Lactobacillus, Pediococcus, Ruminiclostridium, Alloprevotella, Flavobacterium, Lactobacillus, Pediococcus, Dubosiella*.		Reduced serum TC, TG, and LDL-C levels.<br>Increased serum HDL-C level.	
The Antioxidant Dendrobium officinale Polysaccharide Modulates Host Metabolism and Gut Microbiota to Alleviate High-Fat Diet-Induced Atherosclerosis in ApoE−/− Mice	Increased abundance of *Bacteroidota, Desulfobacterota, Allobaculum, Coriobacteriaceae_UCG-00*<br>Decreased abundance of *Firmicutes, unclassified_f__Lachnospiraceae*.<br>Reduced *F/B* ratio.	Increased activity of serum GSH-PX and SOD.<br>Decreased serum MDA levels.<br>Downregulated expression levels of inflammatory factors TNF-α, Il-1β, IL-6.<br>Upregulated expression levels of anti-inflammatory factors Arg1, Mrc1, Retnla, Irf4.	Reduced serum TC, TG, and LDL-C levels.<br>Increased serum HDL-C level.	

The amelioration of a purified Pleurotus abieticola polysaccharide on atherosclerosis in ApoE−/− mice	Increased relative abundance of *Bacteroidetes, Roseburia*.<br>Decreased abundance of *Firmicutes, Adlercreutzia, Turicibacter, Helicobacter*.	Reduced levels of oxidative stress factors (ROS, MDA) in serum and aorta.<br>Enhanced levels of antioxidant factors (SOD, GSH-Px) in serum and aorta.<br>Lowered levels of inflammatory factors (IL-6, TNF-α, IL-1β, IL-18) in serum and aorta.<br>Downregulated expression of TLR4, NLRP3, p-NF-κB in the aorta.	Reduced serum TC, TG, and LDL-C levels.<br>Alleviated liver steatosis.<br>Decreased levels of CER and LPC in serum and aorta.
The effect and mechanism of inulin on atherosclerosis is mediated by the characteristic intestinal flora and metabolites	Decreased abundance of *Firmicutes, Pseudoflavonifractor, Clostridium IV, Alistipes, Mucispirillum, Oscillibacter, Flavonifractor, Streptococcus*.<br>Increased abundance of *Prevotella, Bacteroidetes*.<br>Reduced *F/B* ratio.<br>Lowered serum LPS levels.	Decreased concentrations of inflammatory cytokines TNF-α, IL-1β, IL-6, and IL-17A.	Reduced plasma TC, TG, and LDL-C levels.<br>Decreased levels of bacterial metabolites such as 2-hydroxyhexanoic acid, 2-methylpentanoic acid, 3-methyl-2-oxopentanoic acid, para-aminobenzoic acid, hexanoic acid, and isohexanoic acid.
Whole milk consumption is associated with lower risk of coronary artery calcification progression: evidences from the Multi- Ethnic Study of Atherosclerosis			
The Effects of Moderate Alcohol Consumption on Circulating Metabolites and Gut Microbiota in Patients With Coronary Artery Disease	Increased the abundance of *Paraprevotella* and *Lysinibacillus*.<br>Decreased the abundance of *Bifidobacterium, Megasphaera*, and *Streptococcus*.		Reduced the levels of sphingolipids and glycerophospholipids.
Cow's milk polar lipids reduce atherogenic lipoprotein cholesterol, modulate gut microbiota and attenuate atherosclerosis development in LDL-receptor knockout mice fed a Western-type diet	Increased the abundance of *Bacteroidetes, Actinobacteria,* and *Bifidobacterium*.<br>Decreased the abundance of *Firmicutes*.<br>Reduced the *F/B* ratio.	Downregulated the expression of Ccl4 mRNA in liver tissue.<br>Downregulated the expression of Ccl2 mRNA in the aorta.	Lowered the serum levels of TC, VLDL, and LDL.<br>Reduced the liver TC concentration.<br>Downregulated the expression of Scd mRNA in liver tissue.<br>Upregulated the expression of Hmgcr mRNA in liver tissue.

Dietary Fruit and Vegetable Supplementation Suppresses Diet-Induced Atherosclerosis in LDL Receptor Knockout Mice	Increased gut microbiota diversity.<br>Increased the relative abundance of *Leuconostoc, Trichococcus, Turicibacter,* and *Dorea*.	Reduced the serum TNF-α concentration.	Alleviated liver steatosis.<br>Regulated the serum triglyceride and VLDL -C, and HDL-C concentrations to normal levels.	
Red yeast rice ameliorates high-fat diet-induced atherosclerosis in Apoe−/− mice in association with improved inflammation and altered gut microbiota composition	Decreased the abundance of *Firmicutes, Rikenellaceae, Alistipes, Barnesiella*, and *Flavonifractor*.<br>Increased the abundance of *Bacteroidaceae, Bacteroides, Bacteroides, Anaeroplasma*.	lowered the protein levels of hepatic HMG-CoA reductase.<br>lowered the protein levels of TNF-α and IL-1β.<br>lowered the protein levels of TLR2, TLR4, and MAPK.	Lowered the plasma levels of total CHOL and LDL.	Upregulated the expression levels of JAM-1 and occludin proteins in the intestinal tissue.
Dosage of Dual-Protein Nutrition Differentially Impacts the Formation of Atherosclerosis in ApoE−/− Mice	Reduced the *F/B* ratio.<br>Increased the abundance of *Blautia* and *Akkermansia*.	Lowered the serum levels of IL-1β and TNF-α.<br>Downregulated the expression of ICAM-1.<br>Reduced the number of macrophages in atherosclerotic lesions.	Lowered the plasma levels of TG and LDL-C.	Upregulated the expression levels of tight junction proteins ZO-1 and occludin.
Effects of Whole Brown Bean and Its Isolated Fiber Fraction on Plasma Lipid Profile, Atherosclerosis, Gut Microbiota, and Microbiota-Dependent Metabolites in Apoe−/− Mice	Increased gut microbiota diversity.<br>Reduced the *F/B* ratio.<br>Increased the relative abundance of *unclassified S24-7, Prevotella, Bifidobacterium,* and *unclassified Clostridiales*.<br>Decreased the abundance of *Lactobacillus*.			
Fish Oil Is More Potent than Flaxseed Oil in Modulating Gut Microbiota and Reducing Trimethylamine- N- oxide-Exacerbated Atherogenesis	Lowered the *F/B* ratio.<br>Reduced the concentration of *Desulfovibrionaceae*.<br>Fish oil had increased the abundance of all four SCFA-producing bacteria (*Alistipes, Bifidobacterium, Odoribacter, Parasutterella,* and *Bacteroidales S24-7*).<br>Flaxseed oil had increased the abundance of *Alistipes* and *Odoribacter*.	Lowered plasma TNF-α, IL-1β, and MCP-1 levels.	Lowered plasma TC, TG, and non-HDL-C levels.<br>Reduced liver cholesterol content.<br>Lowered total hepatic fatty acid content.<br>Upregulated CYP7A1 expression.<br>Fish oil had downregulated the transcription and translation levels of HMG-CoA-R.<br>Flaxseed oil had upregulated LDL-R protein expression.	

Camellia oil (Camellia oleifera Abel.) treatment improves highfat diet-induced atherosclerosis in apolipoprotein E (ApoE)−/− mice	Enhanced gut microbiota α-diversity.<br>Increased the Shannon index of gut microbiota.<br>Lowered the relative abundance of *Firmicutes*.<br>Increased the relative abundance of *Bacteroidetes* and *Tenericutes*.<br>Lowered the *F/B* ratio.	Reduced serum IL-6 and TNF-α levels.	Lowered serum TC, TG, and LDL-C levels.
Natto consumption suppresses atherosclerotic plaque progression in LDL receptor‐deficient mice transplanted with iRFP‐expressing hematopoietic cells	Increased the abundance of *Bacillus*.	Lowered the serum levels of CCL2 and IL-1b.<br>Increased the serum level of IL-10.	
Effects of Thermally-Oxidized Frying Oils (Corn Oil and Lard) on Gut Microbiota in Hamsters	Decreased the abundance of *Proteobacteria, Porphyromonadaceae, Coriobacteriaceae, Lachnospiraceae, Prevotellaceae, Bacteroidaceae, Sutterellaceae, Barnesiella, Parasutterella, Olsenella, Lactococcus, Paraprevotella, Clostridium_XIVa*, and *Bacteroides*.<br>Increased the *F/B* ratio.<br>Increased the abundance of *Actinomycetes, Bifidobacteriaceae*, and *Bifidobacterium.*	Increased the plasma level of IL-6.<br>Decreased the plasma level of IL-10.	Increased the plasma levels of TC and TG.
Dietary α-Linolenic Acid-Rich Flaxseed Oil Ameliorates High-Fat Diet-Induced Atherosclerosis via Gut Microbiota-Inflammation-Artery Axis in ApoE−/− Mice	Decreased the *F/B* ratio.<br>Decreased the abundance of *Intestinimonas, Bilophila, Anaerotruncus, Oscillibacter, Negativibacillus, Lachnoclostridium,* and *Enterorhabdus*.	Lowered the plasma levels of TNF-α, IL-1β, and IL-17A.<br>Reduced the levels of TNF-α, IL-1β, IL-6, and IL-17A in the aortic tissue.	
Oral Administration of Branched-Chain Amino Acids Attenuates Atherosclerosis by Inhibiting the Inflammatory Response and Regulating the Gut Microbiota in ApoE-Deficient Mice	Increased the relative abundance of *Ruminiclostridium_5, Faecalibaculum*, and *unclassified_bacterium_f_Lachnospiraceae*.	Reduced the serum levels of MCP-1, IL-1β, and TNF-α.<br>Upregulated the expression of ICAM-1 and VCAM-1 at the root of the aorta.<br>Downregulated the NF-KB/AKT inflammatory pathway.	Lowered the serum levels of TC, LDL-C, and HDL-C.<br>Promoted BA excretion to facilitate cholesterol catabolism.

Medium-, long- and medium-chain-type structured lipids ameliorate high-fat diet-induced atherosclerosis by regulating inflammation, adipogenesis, and gut microbiota in ApoE−/− mice	Increased the abundance of *Bacteroidetes, Blautia*, and *Anaerotruncus*.<br>Decreased the abundance of *Firmicutes, [Ruminococcus] torques group, Ruminiclostridium 9, Catenibacterium,* and *[Eubacterium] fissicatena group*.	Increased the serum T-AOC and SOD levels.<br>Downregulated the expression of VCAM-1, MCP-1, and CD68 in the aortic tissue.	Reduced lipid accumulation in fat tissue.<br>Downregulated the expression of SREBP-1, ACC, FAS, C/EBPα, and PPARγ in epididymal white adipose tissue (eWAT).	
Dietary Egg Sphingomyelin Prevents Aortic Root Plaque Accumulation in Apolipoprotein-E Knockout Mice	Decreased the abundance of *Ruminococcacceae_unclassied*.	Lowered the SAA level.	Reduced epididymal fat accumulation.	
Anti-Atherosclerotic Properties of Wild Rice in Low-Density Lipoprotein Receptor Knockout Mice: The Gut Microbiome, Cytokines, and Metabolomics Study	Increased the abundance of *unclassified Anaeroplasma sp, Acetatifactor muris, unclassified Lactobacillus sp, unclassified Oscillospira sp*, and *Dubosiella newyorkensis*.<br>Decreased the abundance of *unclassified Barnesiella sp, unclassified Butyrivibrio sp,* and *unclassified Oscillibacter sp.*	Lowered the levels of TNF-α, VEGF, and IL-16.<br>Increased the levels of EPO and IL-10.		
Oat fiber supplementation alleviates intestinal inflammation and ameliorates intestinal mucosal barrier via acting on gut microbiotaderived metabolites in LDLR / mice		Downregulated the protein expression of TLR4, MyD88, TRIF, and NF-kB p65 in the aorta.		Alleviated intestinal villous injury.<br>Improved the inflammatory infiltration in the intestinal mucosa, submucosa, and muscle layers.<br>Upregulated the expression of tight junction proteins, including ZO-1 and occludin.<br>Lowered the relative levels of proteins in the intestinal TLR4/NF-kB signaling pathway (including TLR4, MyD88, TRIF, and NF-kB p65) and the NLRP3 inflammasome pathway (including NLRP3, Caspase-1, ASC, IL-1β, and IL-18).
Effects of Different Carbohydrate Content Diet on Gut Microbiota and Aortic Calcification in	Ketogenic diet increased gut microbiota diversity.<br>Ketogenic diet increased the abundance of *Allobaculum* and *Firmicutes.*		Increased the TC level.<br>Lowered the serum TG, LDL-C levels.<br>Increased the serum HDL-C level.	Ketogenic diet alleviated intestinal villous injury, reduced intestinal inflammatory cell infiltration.<br>Ketogenic diet upregulated the expression

Diabetic Mice				of tight junction proteins ZO-1 and Occludin in the intestine.
Whole-Grain Highland Barley Attenuates Atherosclerosis Associated with NLRP3 Inflammasome Pathway and Gut Microbiota in ApoE−/− Mice	Decreased the abundance of *Firmicutes*.<br>Reduced the *F/B* ratio.<br>Increased the abundance of *Bacteroidetes, norank_f_Muribaculaceae, lactic acid bacteria, Lachnospiraceae_NK4A136*, and *Akkermansia*.	Downregulated the expression of the NLRP3 gene.<br>Reduced the serum levels of IL-1β and TNF-α.		
Cabernet sauvignon dry red wine ameliorates atherosclerosis in mice by regulating inflammation and endothelial function, activating AMPK phosphorylation, and modulating gut microbiota	Reduced the *F/B* ratio.<br>Increased the abundance of *Lachnospiraceae, Akkermansiaceae*, and *Muribaculaceae*.<br>Decreased the abundance of *Erysipelotrichaceae* and *Turicibacter*.	Lowered the serum levels of IL-1β, IL-6, and iNOS.<br>Downregulated the protein expression of TLR4, NF-κB, and TNF-α.<br>Increased the serum level of eNOS.	Lowered the serum levels of TC, TG, and LDL-C.<br>Increased the serum HDL-C level.<br>Downregulated the expression of ABCA1, PPARγ, p-AMPK, AMPK, and LXR-α in the liver.<br>Upregulated the expression of SERBP in the liver.	
Gut Microbiota Functional Dysbiosis Relates to Individual Diet in Subclinical Carotid Atherosclerosis	Increased the relative abundance of *Escherichia* and *Oscillospira*.			
The gut microbiome in subclinical atherosclerosis: a population-based multiphenotype analysis	Increased the abundance of *Alistipes, Prevotella, Brevundimonas*, and *Oligella*.<br>Participants with high IMT had highly abundant genera in their feces including *Enterococcus, Methanobrevibacter, Helicobacter, Libanicoccus*, and *Turicibacter*.<br>Participants with high IMT had low abundant genera in their feces including *Faecalicatena, Alistipes, Acinetobacter*, and *Oligella*.			
Dietary titanium dioxide particles (E171) promote diet-induced atherosclerosis through reprogramming gut microbiota-mediated choline metabolism in	Increased the abundance of *Firmicutes, Clostridia, Bacilli, Clostridium XIVa, Eubacterium*, and *Prevotella*.		Inhibited the conversion of cholesterol to bile acids in the liver.	

APOE-/- mice				
Ligustrum Robustum Alleviates Atherosclerosis by Decreasing Serum TMAO, Modulating Gut Microbiota and Decreasing Bile acid and cholesterol absorption in Mice	Increased the abundance of *Actinobacteria, Bifidobacterium,* and *Rikenellaceae_R9_gut_group*.<br>Decreased the abundance of *Prevotellaceae_UCG-001, Lachnospiraceae_NK4A136_group*, and *unclassified_Bacteroidales_bacterium*.	Lowered the protein expression level of F4/80.<br>Upregulated the protein expression of α-SMA.	Lowered the serum and liver cholesterol levels.<br>Promoted the excretion of fecal cholesterol and bile acids.<br>Upregulated the mRNA expression level of the cholesterol transporter Sr-b1 in the liver.<br>Downregulated the mRNA expression level of the key protein Npc1L1 involved in cholesterol absorption in the ileum.<br>Upregulated the mRNA expression level of Abcg8 involved in cholesterol transport to intestinal cells.	
Dietary astaxanthin-rich extract ameliorates atherosclerosis/retinopathy and restructures gut microbiome in apolipoprotein E-deficient mice fed on a high-fat diet	Reduced *F/B* ratio.<br>Decreased the abundance of *Bacteroidetes*, *Firmicutes, Alloprevotella, Desulfovibrio, Muribaculum, Odoribacter, Parabacteroides*.<br>Increased the abundance of *Verrucomicrobia, Akkermansia, Bacteroides, Oscillibacter, Ruminiclostridun_4, Verrucomicrobiae, Clostridia, Verrucomicrobiales, Clostridiales, Akkermansiaceae, Ruminococcaceae.*		Lowered TC and TG levels in the liver.<br>Downregulated mRNA expression of FXR.<br>Downregulated the expression of NPC1L1, ACAT2, and MTTP genes.<br>Inhibited cholesterol reabsorption in the jejunum.<br>Promoted excretion of CA, CDCA, LCA, and DCA.	Upregulated the expression of JAM-A, claudin, and mucin.
Long-chain monounsaturated fatty acids improve endothelial function with altering microbial flora	Reduced *F/B* ratio.<br>Decreased the abundance of *Firmicutes, Clostridium, Lachnospiraceae, Bifidobacterium*.<br>Increased the abundance of *Bacteroidetes*.	Downregulated mRNA expression of MCP-1, ICAM-1, CD68, macrophage SR-a, and inflammatory cytokines IL-6 and TNF-α.<br>SCFAs promote the production of GLP-1 in the plasma and upregulate the expression of GPR43 in the colon.		
Ameliorative effect of purified anthocyanin from Lycium ruthenicum on atherosclerosis in rats through synergistic modulation of the gut microbiota and NF-κB/SREBP-2 pathways	Increased the abundance of *Bifidobacterium, Lactobacillus, Roseburia, Akkermansia,* and *Lachnospiraceae_NK4A136_group*.<br>Decreased the abundance of *Firmicutes* and *Prevotellaceae_NK3B31_group*.<br>Enhanced gut microbiota Chao1 index and Shannon index.	Lowered TNF-α and IL-6 levels.<br>Downregulated the expression of NF-κB and VCAM-1.	Lowered serum TG, TC, and LDL-C levels.<br>Increased serum HDL-C level.<br>Upregulated the protein expression of SREBP-2 and CYP7A1 in the liver.	

Bowman-Birk Major Type Trypsin Inhibitor Derived from Foxtail Millet Bran Attenuate Atherosclerosis via Remodeling Gut Microbiota in ApoE−/− Mice	Increased the abundance of *Bifidobacterium, Lactobacillus, Roseburia, Akkermansia*, and *Lachnospiraceae_NK4A136_group*.<br>Decreased the abundance of *Firmicutes* and *Prevotellaceae_NK3B31_group*.<br>Enhanced gut microbiota Chao1 index and Shannon index.	Lowered TNF-α and IL-6 levels.	Promoted lipid metabolism.
Capsaicin Ameliorates High-Fat Diet-Induced Atherosclerosis in ApoE−/− Mice via Remodeling Gut Microbiota	Increased the abundance of *Deferribacteres, Ileibacterium, Ruminococceae_UCG-014, Odoribacter, Mucispirillum*.<br>Decreased the abundance of *Cyanobacteria, Tenericutes, Faecalibaculum, Marvinbryantia*.<br>Lowered serum LPS levels.	Lowered serum IL-6 levels.	Decreased serum LDL-C levels.<br>Increased serum HDL-C levels.
Eicosapentaenoic Acid-Enriched Phosphoethanolamine Plasmalogens Alleviated Atherosclerosis by Remodeling Gut Microbiota to Regulate Bile Acid Metabolism in LDLR−/− Mice	Decreased the abundance of *Bacteroides*.<br>Increased the abundance of *Clostridium*.		Lowered serum TC and LDL-C levels.<br>Lowered liver TC and TG levels.<br>Increased fecal lipid and bile acid excretion.<br>Decreased CA, increase LCA.<br>Inhibited FXR expression to promote bile acid synthesis, upregulate HMGCR and SREBP-2 mRNA expression, and lower HMGCR protein content.<br>Increased Cyp27a1 and Cyp7b1 mRNA expression to enhance CYP7A1 protein content.
Four Citrus Flavanones Exert Atherosclerosis Alleviation Effects in ApoE−/− Mice via Different Metabolic and Signaling Pathways	Increased the abundance of *Lactobacillus*, *Eubacterium coprostanoligenes, Eubacterium brachy*, and 7α-dehydroxylase-producing bacteria *(Eubacterium coprostanoligenes* and *Eubacterium brachy).*<br>Decreased the abundance of *Bacteroides, Lactococcus, Clostridium sensu stricto 1*, and all BSH-producing bacteria (*Clostridium, Enterococcus, Bacteroides*).		Lowered serum TC and LDL-C levels.<br>Increased serum HDL-C level.<br>Reduced liver TC and TG levels.<br>Decreased HMGCR protein level.<br>Enhanced CYP7A1 protein level.<br>Lowered PCSK9 protein level.
Gallic acid ameliorates atherosclerosis and vascular senescence and remodels the microbiome in a sex-dependent manner in ApoE−/− mice	Reduced *F/B* ratio.<br>Decreased the abundance of *Eubacterium fissicatena* and *Turicibacter*.	Lowered Akt and ERK1/2 phosphorylation induced by Ang II.<br>Reduced IL-3, IL-10, and IL-12 levels in male mice.<br>Lowered IL-10, LIF, and CXCL1 levels in female mice.	

Gut microbiome and metabolomic profiles reveal the antiatherosclerotic effect of indole-3-carbinol in high-choline-fed ApoE-/- mice	Decreased the abundance of *Bacilli, Lactobacillales, Lactobacillaceae, Lactobacillus*, and *Faecalibaculum*. Increased the abundance of *Verrucomicrobia, Verrucomicrobiae, Verrucomicrobiales, Akkermansiaceae,* and *Akkermansia.* Enhanced gut microbiota diversity.		Lowered serum TG levels.	
Helianthus Annuus L. Alleviates High-Fat Diet Induced Atherosclerosis by Regulating Intestinal Microbiota, Inhibiting Inflammation and Restraining Oxidative Stress	Increased the abundance of *Akkermansia muciniphila* and *Lactobacillus*. Decreased the abundance of *unidentified_Enterobateriaceae, Sphingomonas*, and *Methylobacterium.*	Lowered MDA concentration. Increased the concentrations of SOD, NO, and GSH-Px. Downregulated the expression of IL-6, IL-1β, and TNF-α in the aorta. Reduced serum IL-6, IL-1β, and TNF-α levels.	Lowered serum TG, TC, LDL-C levels, and LDL-C/HDL-C ratio. Increased serum HDL-C level.	Enhanced the levels of occludin and ZO-1 in the colon.
Quinic acid regulated TMA/TMAO-related lipid metabolism and vascular endothelial function through gut microbiota to inhibit atherosclerotic	Decreased the abundance of *Streptococcus_danieliae.* Increased the abundance of *Lactobacillus_intestinalis* and *Ileibacterium_valens*.	Inhibited the increase in COX-2, IL-6, E-selectin, ICAM-1, and HMGB1 expression and the phosphorylation of p-P65 and p-MAPK14 proteins induced by TMAO in HCAECs.	Lowered plasma TC, TG, and LDL-C levels. Increased plasma HDL-C level. Inhibited HMGB1 expression and improved TMAO-induced LDL uptake in HCAECs by regulating the HMGB1/SREBP2 axis.	Upregulated the expression of ZO-2, VE-cadherin, and claudin.
Mangiferin alleviates trimethylamine-N-oxide (TMAO)-induced atherogenesis and modulates gut microbiota in mice	Increased the abundance of *Bacteroidetes, Akkermansia, Desulfovibrio*, and *Parabacteroides*. Decreased the abundance of *Firmicutes, Acetatifactor*, and *Blautia*.	Lowered plasma IL-1β and TNF-α levels.	Reduced plasma TC and non-HDL cholesterol levels. Increased the excretion of neutral sterols and acidic sterols in feces, particularly cholesterol and dehydrocholesterol. Downregulated liver FMO3 mRNA expression. Restoried CYP7A1 protein level.	
Millet shell polyphenols prevent atherosclerosis by protecting the gut barrier and remodeling the gut microbiota in ApoE−/− mice	Decreased the abundance of *Verrucomicrobia, Actinobacteria,* and *Allobaculum.* Increased the abundance of *Bacteroidetes, Oscillospira*, and *Ruminococcus*.	Downregulated IL-1β and TNF-α expression in the aorta. Lowered TNF-α and IL-1β levels.		Upregulated the mRNA expression of tight junction proteins occludin, ZO-1, and claudin1 in the intestines.
Naringin Alleviates Atherosclerosis in ApoE−/− Mice by Regulating Cholesterol Metabolism Involved in Gut Microbiota	Increased the abundance of *Firmicutes* and 7α-dehydroxylase-producing bacteria such as *Eubacterium fissicatena, Eubacterium coprostanoligenes,* and *Eubacterium brachy*. Decreased the abundance of *Bacteroidetes, Verrucomicrobia, Bacteroides, Bifidobacterium,*		Lowered serum TC, LDL-C, and TBA levels. Reduced liver TC and TG levels. Decreased the levels of CA,TUDCA,CDCA, and TCA. Promoted the excretion of lipids and bile	

Remodeling	*Lactococcu*s, and *Clostridium sensu stricto.*		acids in feces.	
Atherosclerosis amelioration by allicin in raw garlic through gut microbiota and trimethylamine-N-oxide modulation	Enhanced gut microbiota alpha diversity.<br>Increased the abundance of *Akkermansia, Desulfovibrio, Christensenellaceae R-7, Lachnospiraceae UCG-008, Faecalibacterium prausnitzii,* and *Akkermansia*.			
Peanut skin extract ameliorates high-fat diet-induced atherosclerosis by regulating lipid metabolism, inflammation reaction and gut microbiota in ApoE / mice	Increased the abundance of *Bacteroidetes, Roseburia, Rothia, Parabacteroides,* and *Akkermansia.*<br>Decreased the abundance of *Firmicutes, Bilophila,* and *Alistipes*.<br>Reduced *F/B* ratio.	Lowered the levels of pro-inflammatory cytokines TNF-α and IL-6.<br>Increased the level of anti-inflammatory factor IL-10.<br>Reduced MDA content.<br>Increased GSH level.	Lowered serum TC and LDL-C levels.<br>Increased serum HDL-C level.	
Polyphenols from hickory nut reduce the occurrence of atherosclerosis in mice by improving intestinal microbiota and inhibiting trimethylamine N-oxide production	Decreased the abundance of *Desulfovibrio* and *Lachnochlostrium*.<br>Increased the abundance of *unclassified_Lachnospiraceae* and *Romboutsia*.		Lowered TG, TC, LDL, and TNF-α levels.<br>Increased HDL-C, adiponectin, and APOA1 levels.	
Protocatechuic acid alleviates TMAO-aggravated atherosclerosis via mitigating inflammation, regulating lipid metabolism, and reshaping gut microbiota	Enhanced gut microbiota alpha diversity.<br>Increased the abundance of *Actinobacteria, Bifidobacteriaceae, Bifidobacterium, Olsenella, Rikenella, Turicibacter, Clostridium_sensu_stricto,* and *Bifidobacterium*.<br>Decreased the abundance of *Peptostreptococcaceae, Lactococcus,* and *Enterorhabdus*.	Lowered plasma TNF-α, MCP-1, IL-1β, and IL-6 levels.<br>Increased PPARα protein level.	Promoted the excretion of total FAs, SFAs, MUFAs, and PUFAs in fecal fatty acids.<br>Downregulated the mRNA expression levels of FAS and SREBP1c.	
Amelioration of Atherosclerosis by lycopene is linked to the modulation of gut microbiota dysbiosis and related gut-heart axis	Enhanced gut microbiota alpha diversity.<br>Reduced *F/B* ratio.<br>Increased the abundance of *Akkermansia, Alloprevotella, Bacteroidetes,* and *Verrucomicrobia*.<br>Decreased the abundance of *Firmicutes*.<br>Lowered serum LPS level.	Reduced TNF-α, MCP-1, IL-6, and IL-1β levels.<br>Downregulated the expression of TLR4 and phosphorylated NF-κB p65 in aortic sinus plaques.<br>Lowered serum MCP-1, TNF-α, IL-1β, and	Lowered serum TC and LDL-C levels.<br>Increased serum HDL-C level.	Upregulated the expression of ZO-1 and occludin in the intestines.

activation in high-fat diet-fed ApoE−/− mice		IL-6 levels.<br>Downregulated the expression of TLR4 and phosphorylated NF-κB p65 in aortic sinus plaques.		
Anti-atherosclerotic effects of geraniin through the gut microbiota-dependent trimethylamine N-oxide (TMAO) pathway in mice	Increased the abundance of *Bacteroidetes, Firmicutes, Bacteroides, Alloprevotella,* and *Alistipes*.<br>Decreased the abundance of *Sphingomonas* and *Rhizobium*.	Reduced TMAO-induced M1 macrophage aggregation.<br>Lowered plasma IL-1β, IL-6, and TNF-α levels.<br>Increased plasma IL-10 level.		
Berberine attenuates choline-induced atherosclerosis by inhibiting trimethylamine and trimethylamine-N-oxide production via manipulating the gut microbiome	Increased the abundance of *Lachnospiraceae NK4A136 group, Bacteroidales S24-7 group (unclassified), Eubacterium, Bacteroides, Prevotella, Parabacteroides*, and *Alloprevotella*.<br>Inhibited the ability of four TMA-producing strains (*Clostridium sporogenes, Anaerococcus hydrogenalis*, etc.) to convert d9-choline to d9-TMA.			
Butanol Extract of Acanthopanax senticosus (Rupr. etMaxim.) Harms Alleviates Atherosclerosis inApolipoprotein E-Deficient MiceFed a High-Fat Diet	Reduced *F/B* ratio.<br>Increased the abundance of *lactobacilli, Bacteroidetes, Prevotella, Ruminococcus*, and *Stutteralla*.<br>Decreased the abundance of *Desulfovibrio*.	Lowered the levels of serum IL-1β, IL-6, TNF-α, and iNOS.<br>Increased the level of SOD.<br>Reduced the levels of cytokines such as IL-1β, IL-6, IL-7, TNF-α, and TNF-β in liver tissue.<br>Downregulated the expression of NOS in the liver.<br>Increased the levels of SOD, CAT, and GSH in the liver.	Lowered serum TC, TG, and LDL-C levels.<br>Increased serum HDL-C level.	
Crocin mitigates atherosclerotic progression in LDLR knockout mice by hepatic oxidative stress and inflammatory reaction reduction, and intestinal barrier improvement and gut microbiota modulation	Reduced *F/B* ratio.<br>Enhanced gut microbiota alpha diversity.<br>Decreased the abundance of *Erysipelotrichia, Erysipelotrichales*, and *Parabacteroides*.<br>Increased the abundance of *Bacilli* and *Lactobacillales*.	Downregulated the expression levels of NF-κB p65, NLRP3, ASC, Caspase-1, IL-1β, and IL-18.<br>Inhibited the TLR4/MyD88 signaling pathway.<br>Lowered the levels of NF-κB p65, TLR4, MyD88, TRAF6, TRIF, RIP, and IKKβ.	Reduced the triglyceride concentration in liver homogenate.	

Curcumin attenuates cadmium-induced atherosclerosis by regulating trimethylamine-N-oxide synthesis and macrophage polarization through remodeling the gut microbiota	Reduced *F/B* ratio.<br>Increased the abundance of *Verrucomicrobia, Unspecified_S24_7,* and *Akkermansia*.<br>Decreased the abundance of *Lactobacillaceae* and *Lactobacillus*.	Downregulated the expression of NF-KB p65 and NLRP.<br>Lowered the plasma levels of IL-1β and IL-6.<br>Restored macrophage polarization, reduced M1 macrophages, and increased M2 macrophages.	Lowered plasma LDL, TG, and T-CHO levels.<br>Increased plasma HDL-C level.	
Gypenoside XLIX Ameliorate High-Fat Diet-Induced Atherosclerosis via Regulating Intestinal Microbiota, Alleviating Inflammatory Response and Restraining Oxidative Stress in ApoE−/− Mice	Decreased the abundance of gut bacteria that produce trimethylamine such as *Clostridioides* and *Desulfovibrionaceae.*<br>Increased the abundance of bacteria that produce butyrate such as *Eubacterium, Roseburia, Bifidobacterium, Lactobacillus*, and *Prevotella*.	Increased the activity of GSH-Px and SOD.<br>Lowered MDA concentration.<br>Downregulated the mRNA expression levels of TNF-α, IL-6, and IL-1β in the liver.<br>Downregulated the mRNA expression levels of ICAM-1 and CCL2 in the liver.	Lowered plasma TC, LDL-C, and TG levels.<br>Increased plasma HDL-C level.	
Ginkgo biloba extract ameliorates atherosclerosis via rebalancing gut flora and microbial metabolism	Reduced *F/B* ratio.<br>Increased the abundance of *Desulfobacteria, Bacteroidetes, Verrucomicrobia, norank Desulfovibrionaceae, Akkermansia, Alistipes, Rikenellaceae RC9Alloprevotella*, and *Parabacteroides*.<br>Decreased the abundance of *Firmicutes (Blautia, norank Lachnospiraceae)* and *Actinobacteriota*.	Downregulated the mRNA expression of CCL2, IL-1β, and TNF-α in the artery.<br>Downregulated the mRNA expression levels of ICAM-1 and VCAM-1 in the artery.<br>Lowered the mRNA expression of CCL2, IL-6, and TNF-α in the colon.	Lowered serum TC, LDL-c, and TG levels.<br>Inhibited macrophage cholesterol uptake by downregulating the transcription levels of macrophage markers Cd68 and scavenger receptor Cd36 and Sra1 in the artery.<br>Increased the concentrations of secondary bile acids such as LCA, TUDCA, and THDCA.<br>Lowered the levels of primary bile acids CA and β-MCA.	Increased the levels of ileal Claudin-1 and ZO-1 protein.
Ginkgolide B treatment regulated intestinal flora to improve high-fat diet induced atherosclerosis in ApoE / mice	Reduced abundance of *Firmicutes, Deferrobacters, Helicobacter*, and *Roseburia*.<br>Increased abundance of *Bacteroides, Bacteroidetes*, and *Prevotella*.		Lowered plasma TC, TG, VLDL-C, and LDL-C levels.<br>Increased plasma HDL-C levels.	
Ginsenosides retard atherogenesis via remodelling host–microbiome metabolic homeostasis	Reduced abundance of *Desulfobacterota*.<br>Increased abundance of *Verrucomicrobiota, Alloprevotella, Akkermansia, Lactobacillus, Eubacterium fissicatena group*, and *Ruminococcus torques group.*	Lowered serum CRP, CCL2, and IL-1β levels.<br>Downregulated mRNA expression of CCl2, IL-1β, IL-6, TNFα, ICAM1, and VCAM1 in the aorta.	Alleviated liver fat accumulation, lipid droplet formation, and vacuolar degeneration.<br>Lowered serum TC, TG, and LDL-C levels.<br>Increased abundance of Lactobacillus	Upregulated expression of MUC2, TFF2, and tight junction proteins (TJP1, ZO-1, and Occludin) in the colon.

			and its BSH coding genes, promoting hydrolysis of conjugated bile acids. Increased fecal UDCA, HDCA, β-MCA, α-MCA, and DCA free bile acid proportions, thereby promoting total bile acid excretion.	
Grape Seed Proanthocyanidin Extract Alleviates Atherosclerosis by Modulating the Production of Trimetlylamine N-Oxide	Reduced abundance of *Firmicutes, Deferribacteres, Helicobacter*, and *Roseburia*. Increased abundance of *Bacteroidetes, Proteobacteria, Prevotella,* and *Bacteroides*.		Lowered TG, TC, and LDL-C levels.	
Ferulic Acid Ameliorates Atherosclerotic Injury by Modulating Gut Microbiota and Lipid Metabolism	Reduced abundance of *Firmicutes, Erysipelotrichaceae*, and *Ileibacterium*. Increased abundance of *Bacteroidetes, Ruminococcaceae*, and *Lactobacillus*.	Lowered MDA activity. Increased SOD activity.	Lowered plasma TC, TG, and LDL-C levels. Downregulated mRNA levels of SREBP1 and ACC. Decreased protein levels of SREBP1 and ACC. Increased protein levels of AMPKα and phosphorylated AMPKα.	
Guggulsterone, a farnesoid X receptor antagonist lowers plasma trimethylamine-N-oxide levels: An evidence from in vitro and in vivo studies		Lowered serum TNF-α, IL-6, and IL-1β levels. Reduced MDA levels. Increased GSH levels. Enhanced SOD and CAT activities.	Lowered serum TC, TG, LDL-C, VLDL-C and ApoB levels. Increased serum HDL-C levels.	
Gut Microbiota Composition Affects Procyanidin A2-Attenuated Atherosclerosis in ApoE−/− Mice by Modulating the Bioavailability of Its Microbial Metabolites	Lowered *F/B* ratio. Increased abundance of *Verrucomicrobia, Akkermansia, Prevotellaceae*, and *Coriobacteriaceae_UCG-00*.	Enhanced SOD activity. Lowered VCAM-1 and ICAM-1 levels. Upregulated mRNA expression of PPARγ, CYP7A1, and ABCA1. Downregulated mRNA expression of ICAM-1 and VCAM-1.	Lowered TG and LDL-C levels. Increased HDL-C levels.	
Gut Parabacteroides merdae protects against cardiovascular damage by enhancing branched-chain amino	Increased abundance of *Parabacteroides* and *Akkermansia*.	Lowered plasma MDA and hs-CRP levels.	Lowered plasma TC, TG, and LDL-C levels.	

acid catabolism				
Inflammation inhibition and gut microbiota regulation by TSG to combat atherosclerosis in ApoE−/− mice	Reduced abundance of *Bacteroidetes, Proteobacteria, Tenericutes*, and *Akkermansia*.<br>Increased abundance of *Firmicutes* and *Helicobacter pylori.*<br>Increased *F/B* ratio.	Lowered serum IL-6, TNF-α, VCAM-1, and MCP-1 levels.<br>Downregulated expression of VCAM-1, ICAM-1, and CCRA in aortic tissue.	Lowered TG and ox-LDL levels.	
Isolation of melanoidins from heat-moisture treated ginseng and its inhibitory effect on choline metabolism	Lowered *F/B* ratio.<br>Reduced abundance of *Firmicutes, [Eubacterium]_xylanophilum_group*, and *Burkholderia-Caballeronia-Paraburkholderia*.<br>Increased abundance of *Parabacteroides, Bifidobacterium, Lactobacillus*, and *Candidatus_Saccharimonas.*		Lowered serum TC, TG, and LDL-C levels.<br>Increased serum HDL-C levels.	
Gut microbiota and diet matrix modulate the effects of the avonoid quercetin on atherosclerosis	Increased abundance of *Eggerthellaceae, Ruminococcaceae, Desulfovibrionaceae, Parvibacter, Dorea, Ruminiclostridium, Eubacterium xylanophilum group*, and *Eggerthellaceae*.<br>Decreased abundance of *Lactobacillaceae*.<br>Enhanced gut microbiota Shannon index.			
Puerarin alleviates atherosclerosis via the inhibition of Prevotella copri and its trimethylamine production	Decreased abundance of *Prevotella copri*.<br>Enhanced gut microbiota α-diversity.	Reduced levels of TNF-α,IL-6, IL-1β, and adhesion molecules such as ICAM-1 and VCAM-1.<br>Lowered cholesterol levels.	Lowered cholesterol levels.	
Integration of 16S rRNA sequencing and metabolomics to investigate the modulatory effect of ginsenoside Rb1 on atherosclerosis	Enhanced gut microbiota α-diversity index.<br>Decreased *F/B* ratio.<br>Increased abundance of *Bacteroidetes, Actinobacteria, Lactobacillus, Prevotella*, and *Oscillospira*.<br>Decreased abundance of *Desulfovibrio*.	Reduced plasma concentrations of IL-1β, TNF-α, IL-6, and IL-18.<br>Lowered protein expression of NF-κB, Caspase-1, and NLRP.	Alleviated hepatic steatosis in liver cells.<br>Lowered serum TC, TG, and LDL-C levels.	
Usnea improves high-fat dietand vitamin D3-induced atherosclerosis in rats by remodeling intestinal flora homeostasis	Increased abundance of *Bacteroidetes.*<br>Decreased abundance of *Akkermansia, Verrucomicrobiota,* and *Proteobacteria*.<br>Reduced serum LPS levels.	Lowered serum TNF-α and IL-6 levels.	Lowered serum TC, TG, and LDL-C levels.<br>Upregulated CYP7A1 protein expression.<br>Enhanced BA levels.	Upregulated ZO-1 and occludin protein expression.

Berberine treats atherosclerosis via a vitamine-like effect downregulating Choline-TMA-TMAO production pathway in gut microbiota	Decreased abundance of TMA-producing bacteria in hamster intestines such as *Proteus mirabilis, Shigella baumannii, Bacteroides fragilis*.<br>Decreased abundance of TMA-producing bacteria in atherosclerosis patients such as *Eubacterium coprostanoligenes group, Eubacterium hallii group*.		
Notoginsenoside R1 Ameliorate High-Fat-Diet and Vitamin D3-Induced Atherosclerosis via Alleviating Inflammatory Response, Inhibiting Endothelial Dysfunction, and Regulating Gut Microbiota	Decreased abundance of *Firmicutes* and *Proteobacteria.*<br>ncreased abundance of *Bacteroidetes.*	Lowered plasma levels of IL-6, IL-33, TNF-α, and IL-1β.<br>Downregulated aortic VCAM-1 and ICAM-1 expression levels.<br>Enhanced aortic NO and eNOS expression levels.<br>Downregulated endothelial cell NLRP3, Cleaved Caspase-1, and IL-1β expression.	Lowered plasma TG, TC, LDL-C, and ox-LDL levels.<br>Increased plasma HDL-C levels.
Microbiome and metabonomics study of quercetin for the treatment of atherosclerosis	Increased abundance of *Phascolarctobacterium* and *Anaerovibrio.*	Lowered plasma TNF-α and IL-6 levels.	Lowered plasma TC, TG, HDL, and LDL-C levels.<br>Promoted primary bile acid biosynthesis.
Paeonol reduces microbial metabolite α-hydroxyisobutyric acid to alleviate the ROS/TXNIP/NLRP3 pathway-mediated endothelial inflammation in atherosclerosis mice	Decreased *F/B* ratio.<br>Decreased abundance of *Acetatifactor, Helicobacter,* and *Mucispirillum.*<br>Increased abundance of *Bacteroides, Alistipes,* and *Barnesiella*.<br>Enhanced gut microbial α-diversity and β-diversity.	Lowered serum TNF-α, IL-1β, and IL-18 levels.	Lowered serum TC, LDL-C, and TG levels.<br>Increased serum HDL-C levels.
Ginger essential oil and citral ameliorates atherosclerosis in ApoE−/− mice by modulating trimethylamine-N-oxide and gut microbiota	Enhanced gut microbiota Shannon diversity index.<br>Increased abundance of *Akkermansia, Allobaculum, Bifidobacterium,* and *Alistipes.*<br>Decreased abundance of *Enterorhabdus* and *Proteus*.	Lowered plasma TNF-α, IL-6, and IL-1β levels.	Lowered plasma TC and LDL-C levels.<br>Increased plasma HDL-C levels.
Quercetin reduces atherosclerotic lesions by altering the gut microbiota and reducing atherogenic lipid metabolites	Increased abundance of *Actinobacteria, Bacteroidetes, Akkermansia, Bacteroides, Parabacteroides,* and *Ruminococcus*.<br>Decreased abundance of *Firmicutes* and *Lactobacillus*.	Lowered plasma MDA levels.<br>Increased IL-6 levels.	Lowered levels of pro-atherosclerotic lipid metabolites in the gut (such as LPC 18:1, LPA 18:1, LPA 18:2, LPA 20:4, PEIPC, POVPC, and PGPC).<br>Increased fecal sterol levels.<br>Lowered total cecal bile acids and

			cholesterol levels.	
Remodelling of gut microbiota by Berberine attenuates trimethylamine N-oxide-induced platelet hyperreaction and thrombus formation	Decreased abundance of *Bacteroidetes, Blautia, Parashuttella, Roseburia, Lachnospiraceae incertae sedis, Actinomyces, Actinomycetaceae, Clostridium XIVa, Rothia, Micrococcaceae, Ruminococcaceae, Clostridiales, Clostridia*, and *Firmicutes*.<br>Increased abundance of *Lactobacillus, Gammaproteobacteria, Enterobacteriales, Enterobacteriaceae, Proteobacteria, Escherichia_Shigella, Butyricimonas, Verrucomicrobiales, Akkermansia, Verrucomicrobia, Verrucomicrobiae, Verrucomicrobiaceae, Clostridium sensu stricto,* and *Clostridiaceae.*<br>Lowered gut microbiota α-diversity index.	Inhibited TMAO-induced high platelet reactivity to collagen by promoting phosphorylation of ERK1/2 and JNK.		
Paeonol Attenuated Vascular Fibrosis Through Regulating Treg/Th17 Balance in a Gut Microbiota-Dependent Manner	Increased abundance of SCFA-producing microbiota such as *Clostridia, Clostridium IV, Lachnospiraceae*, and *Lactobacillus*.	Reduced Th17 cell numbers and increased Treg cell numbers, thereby improving the Treg/Th17 balance.<br>Downregulated expression of pro-inflammatory cytokines (such as IL-1β, IL-6, TNF-α, and IL-17A).<br>Upregulated expression of anti-inflammatory cytokine IL-10.<br>Downregulated α-SMA protein expression, alleviating vascular fibrosis.<br>Downregulated LOX, MMP-2/9, and collagen I/III expression, inhibiting vascular fibrosis.		
Inhibiting vascular smooth muscle cell proliferation mediated by osteopontin via regulating gut microbial lipopolysaccharide: A novel mechanism for paeonol in atherosclerosis treatment	Decreased abundance of Gram-negative bacteria.<br>Lowered serum LPS concentration.	Downregulated protein expression of α-SMA and PCNA.<br>Downregulated mRNA expression of OPN.		Upregulated expression of tight junction proteins (claudin-1, occludin, and ZO-1).
Astragaloside IV Mediates the PI3K/Akt/mTOR Pathway	Increased abundance of *Bifidobacterium, Lactobacillus*, and *Bacteroides.*<br>Decreased abundance of *Enterobacter,*	Increased serum NO levels.<br>Lowered serum ET-1 and Ang-II levels.<br>Inhibited PI3K/Akt/mTOR signaling	Lowered serum TC, TG, and LDL-C levels.<br>Increased serum HDL-C levels.	

to Alleviate Injury and Modulate the Composition of Intestinal Flora in ApoE−/− Atherosclerosis Model Rats	*Enterococcus, Fusobacterium*, and *Clostridium*.	pathway to reduce TNF-α, IL-6, IL-1β, VCAM-1, MMP-2, MCP-1, and ICAM-1 mRNA levels in aortic root tissue.	
The Role of Ophiopogonin D in Atherosclerosis: Impact on Lipid Metabolism and Gut Microbiota	Increased abundance of *Bacteroidetes* and *Faecalibaculum*.<br>Decreased abundance of *Firmicutes* and *Ileibacterium*.	Lowered MDA and LDH levels.<br>Increased SOD levels.	Lowered serum TG, TC, and LDL-C levels.<br>Alleviated hepatic steatosis in liver cells.<br>Downregulated mRNA expression of mTOR, Srebp1, Acc, and Scd.<br>Lowered protein levels of mTOR and p-mTOR.
Thelenota ananas saponin extracts attenuate the atherosclerosis in apoE−/− mice by modulating lipid metabolism	Increased abundance of *Bacteroides, Blautia, Eubacterium xylanophilum, Lachnospiraceae, Alistipes, Anaerotruncus, Lactobacillus*, and *Ruminiclostridium*.<br>Decreased abundance of *Alloprevotella.*<br>Enhanced gut microbiota α-diversity.	Lowered serum IL-1β and IL-6 concentrations.	Lowered serum TCHO and TG levels.<br>Increased serum HDL-C levels.<br>Elevated protein levels of CYP27A1, CYP1B1, NPC1, ABCA1, COX2, and SRBI in the liver.
Tilianin improves lipid profile and alleviates atherosclerosis in ApoE / mice through up-regulation of SREBP2-mediated LDLR expression	Lowered *F/B* ratio.<br>Increased abundance of *Bacteroidetes, Faecalibaculum*, and a genus under *Bacteroidales S27 group.*<br>Decreased abundance of *Firmicutes*.		Lowered serum TC and LDL-C levels.<br>Alleviated hepatic lipid accumulation.<br>Increased LDLR protein levels in the liver and LDL-C uptake.<br>Enhanced LDLR protein levels by activating SRE-1/SREBP2-regulated transcription of the LDLR gene.
Effect of Berberine on Atherosclerosis and Gut Microbiota Modulation and Their Correlation in High-Fat Diet-Fed ApoE−/− Mice	Increased abundance of *Turicibacter, Alistipes, Roseburia, Allobaculum,* and *Blautia.*<br>Decreased abundance of *Bilophila*.	Lowered levels of pro-inflammatory cytokines such as TNF-α, IL-1β, IL-6, and Hs-CRP.<br>Increased levels of anti-inflammatory cytokines IL-10 and ADPN.	Lowered TC, TG, and LDL-C levels.<br>Increased HDL-C level.
β-sitosterol inhibits trimethylamine production by regulating the gut microbiota and attenuates atherosclerosis in ApoE−/− mice	Increased abundance of *Actinobacteriota, Bacteroidota, Desulfobacterota, Firmicutes, Weissella, Eubacterium, Lactobacillus,* and *Butyricioccus*.<br>Decreased abundance of *Proteobacteria, Verrucomicrobiota, Klebsiella, Clostridioides, Desulfovibrionaceae,* and *Prevotella*.	Lowered serum TNF-α and IL-6 levels.<br>Increased GSH-Px and SOD levels.<br>Lowered MDA levels.	Lowered serum TC and LDL-C levels.

Ginsenoside Rc ameliorated atherosclerosis via regulating gut microbiota and fecal metabolites	Increased abundance of *Bacteroidetes, Muribaculaceae, Lactobacillus, Ileibacterium, Bifidobacterium, Burkholderiales, Prevotellaceae, Erysipelotrichaceae, Sutterellaceae, Alloprevotella, Lachnoclostridium, Allobaculum*, and *Parasutterella.* Decreased abundance of *Firmicutes, Faecalibaculum, Oscillibacter, Eubacterium coprostanoligenes group,* and *Blautia.*	Lowered serum TNF-α, IL-6, and IL-1β levels.	Lowered serum TC, TG, and LDL-C levels.<br>Increased serum HDL-C level.<br>Promoted taurine and hypotaurine metabolism, arginine biosynthesis.<br>Inhibited the TCA cycle.
Banxia Xiexin decoction alleviates AS co-depression disease by regulating the gut microbiome-lipid metabolic axis	Decreased the abundance of *Proteobacteria, Deferribacteres, Clostridium_IV, Helicobacter, Pseudoflavonifractor, Acetatifactor*, and *Oscillibacter*.		Reduced the plasma levels of LDL-C, HDL-C, and TG.<br>Decreased the levels of LPC(20:3) in the hippocampus and LPC in the prefrontal cortex.
Buyang Huanwu Decoction Alleviates Atherosclerosis by Regulating gut Microbiome and Metabolites in Apolipoprotein E-deficient Mice fed with High-fat Diet	Decreased the abundance of *Cytophagales, Myxococcales, Rhizobiales, Sporocytophag*a, and *Acidibacter*.	Reduced the levels of serum inflammatory cytokines such as IL-1β, MCP-1, IL-6, KC, and TNF-α.	Reduced the serum levels of TG and TC.<br>Regulated the metabolism of phenylalanine, tyrosine, tryptophan biosynthesis, biosynthesis of unsaturated fatty acids, phenylalanine metabolism, and linoleic acid metabolism.
Dingxin Recipe IV attenuates atherosclerosis by regulating lipid metabolism through LXR-α/SREBP1 pathway and modulating the gut microbiota in ApoE-/- mice fed with HFD	Increased the abundance of *Bacteroides, Muribaculaceae*, and *Ruminococcacea*e.<br>Decreased the abundance of *Firmicutes* and *Erysipelotrichaceae*.<br>Reduced the *F/B* ratio.		Reduced the serum levels of TG, TC, and LDL-C.<br>Downregulated the expression of lipid metabolism genes LXR-α, SREBP1, and ACC1 in the liver.<br>Upregulated the expression of the lipid metabolism gene ABCA1 in the liver.
HuangQi ChiFeng decoction maintains gut microbiota and bile acid homeostasis through FXR signaling to improve atherosclerosis	Increased the alpha diversity index of the gut microbiota.<br>Increased the abundance of *Alistipes, Rodentibacter, Acetatifactor, Peptococcus, Anaeroplasma, Bacteroides, Parasutterella, Mucispirillum, Butyricicoccus, Parabacteroides, Rodentibacter.*<br>Decreased the abundance of *Desulfobacterota, Deferribacterota, Proteobacteria, Clostridia_UCG-014, Lactobacillus, Lachnospiraceae_UCG-006*, and *Alistipes*.		Improved bile acid metabolism by reducing LCA, DCA, UDCA, GLCA, TLCA, and GDCA.<br>Upregulated expression of CYP8B1 and CYP27A1, inhibited FXR expression, increased CYP7A1 expression, accelerated cholesterol metabolism and bile acid synthesis, and alleviated cholesterol accumulation.<br>Upregulated expression of LXRα,

			ABCA1, and ABCG1, promoting cholesterol metabolism.	
Guanxin Xiaoban capsules could treat atherosclerosis by affecting the gut microbiome and inhibiting the AGE–RAGE signalling pathway	Increased the abundance of *Actinobacteria* and *Akkermansia.*<br>Decreased the abundance of *Faecalibaculum*.	Downregulated the expression of RAGE, VEGFA, and CDKN1A.<br>Upregulated the expression of GSTM1.		
Guanxinning Tablet Attenuates Coronary Atherosclerosis via Regulating the Gut Microbiota and Their Metabolites in Tibetan Minipigs Induced by a High-Fat Diet	Increased the abundance of *Prevotellaceae* and *Prevotella*.<br>Decreased the abundance of *Proteobacteria, Enterobacteriaceae*, and *Escherichia*.	Reduced the serum levels of ox-LDL, CRP, TNF-α, IL-1β, MDA, vWF, and ET-1.<br>Increased the serum level of SOD.<br>Reduced the expression of NF-κB, TNF-α, and MMP-9 in the aorta.	Reduced the serum LDL-C level.<br>Increased the serum HDL-C level.<br>Reduced the content of CDCA.<br>Increased the content of LCA-3S.	
The Enhanced Pharmacological Effects of Modified Traditional Chinese Medicine in Attenuation of Atherosclerosis Is Driven by Modulation of Gut Microbiota	Increased the abundance of *Enterobacter, Streptococcus, Blautia, Clostridiacea, Adlercreutzia, Pantoea, Allobaculum.*	Reduced the serum levels of hs-CRP and IFN-γ.<br>Increased the serum level of IL-4.	Reduced the serum levels of TC, TG, and LDL-C.<br>Increased the serum level of HDL-C.	
Naoxintong capsule remodels gut microbiota and ameliorates early-stage atherosclerosis in apolipoprotein E-deficient mice	Increased the abundance of *Akkermansia, Faecalibacterium, Prevotella, Bifidobacterium*.<br>Reduced the serum LPS level.	Reduced the serum levels of TNF-α, IL-6L.	Alleviated liver steatosis.<br>Reduced the plasma levels of TC, TG, and LDL-C.<br>Increased the plasma HDL-C level.	Upregulated the expression of tight junction proteins (ZO-1, Claudin-1) in the intestine.
Tongxinluo May Alleviate Inflammation and Improve the Stability of Atherosclerotic Plaques by Changing the Intestinal Flora	Increased the abundance of *Bacteroides*.<br>Reduced the *F/B* ratio.	Reduced the concentration of TNF-α.<br>Downregulated the expression of NLRP3, caspase-1, IL-1β, and IL-18.	Not affected blood lipid levels.	

Qing-Xin-Jie-Yu Granule alleviates atherosclerosis by reshaping gut microbiota and metabolic homeostasis of ApoE-/- mice	Increased the abundance of *Roseburia, Aerococcus, Enterobacter, Monoglobus*.<br>Decreased the abundance of *Alistipes, Rikenella*, and *Blautia.*	Reduced the serum levels of TNF-α, IL-1β, and IL-6.	Reduced the serum levels of TC, TG, and LDL-C.<br>Increased the serum level of HDL-C.	
Xinnaokang improves cecal microbiota and lipid metabolism to target atherosclerosis	Increased the abundance of *Bifidobacteriales, Bifidobacteriaceae, Marinifilaceae, Rikenellaceae, Tannerellaceae, Deferribacteraceae, Deferribacterales, Deferribacteres, Planococcaceae, Carnobacteriaceae, Christensenellaceae, Clostridiaceae_1, Clostridiales_vadinBB60_group, Family_XIII, Lachnospiraceae, Ruminococcaceae, un_o_Clostridiales, Clostridia, un_p_Firmicutes.*		Decreased the TC, TG, and LDL-C levels.<br>Increased the HDL-C level.<br>Upregulated the expression of ABCA1, SREBP-1, and LXR in vascular tissue.<br>Upregulated the expression of SREBP-2 and LDLR in liver tissue.<br>Downregulated the expression of IDOL in liver tissue.	
Efficacy of Spleen-and-Stomach-Tonifying, Yin-FirePurging, and Yang-Raising Decoction Derived from the Trimethylamine N-Oxide Metabolic Pathway of Intestinal Microbiota on Macrovascular Lesions Caused by Type 2 Diabetes Mellitus	Increased the abundance of *Faecalibacterium* and *Ruminococcus.*<br>Decreased the abundance of *Colidextribacter, Papilibacter*, and *Desulfovibrio.*	Increased the concentration of SOD.<br>Decreased the concentrations of MDA, ICAM-1, VCAM-1, IL-6, and hs-CRP.	Decreased the serum levels of TC, TG, and LDL-C.	
Alisma orientalis Beverage Treats Atherosclerosis by Regulating Gut Microbiota in ApoE-/- Mice	Increased the abundance of *Actinobacteria, Bifidobacterium*, and *Lachnospiraceae_NK4A136_group*.<br>Decreased the abundance of *Firmicutes* and *Lactobacillus*.<br>Improved the diversity of the gut microbiota.	Reduced levels of serum IL-6, TNF-α, IL-1β, and IL-17.	Decreased the serum levels of TC, TG, and LDL-C.<br>Increased the HDL-C level.	
Bicyclol Alleviates Atherosclerosis by Manipulating Gut Microbiota	Increased the abundance of *Alloprevotella, Bacteroides, Prevotellaceae, Ruminococcaceae, Clostridum,* and *Eubacterium.*<br>Increased the abundance of SCFA-producing genera: *Clostridum* and *Prevotellaceae for acetate, Blautia for propionate, Bacteroides, Ruminococcaceae, Eubacterium, Lachnospiraceae_UCG-006,* and *Alloprevotella*.<br>Reduced the *F/B* ratio.	Decreased the number of monocytes (F4/80+/CD11b+) in the aortic arch.<br>Downregulated the expression of pro-inflammatory cytokines such as IL-1a, IL-1b, IL-2, IL-6, IL-9, IL12(p40), IL-12(p70), G-CSF, KC, TNF-α, IL17, and MIP-1b in the aortic arch.<br>Upregulated the expression of anti-inflammatory cytokines such as IL-3,	Reduced the level of CE in the aorta.<br>Downregulated the expression of ICAM-1 and VCAM-1 in the aorta.<br>Downregulated the expression of tlr4 and nfκb in the liver.<br>Upregulated the expression of cyp7a1 and abca1 in the liver.<br>Reduced the level of cholesterol esters in the liver.	Upregulated the expression of tight junction proteins (occludin and ZO-1) in the intestinal epithelium.

		IL-4, IL-10, IL-13, and IL-1.	Increased the level of SCFAs.
Aspirin ameliorates atherosclerotic immuno-inflammation through regulating the Treg/Th17 axis and CD39-CD73 adenosine signaling via remodeling the gut microbiota in ApoE-/- mice	Increased the abundance of *Bacteroidetes, Bacteroides, Mucispirillum, Prevotellaceae_UCG_001,* and *Bilophila.*<br>Reduced the *F/B* ratio.	Increased the proportion of Tregs and the proportion of CD39+ and CD73+ Tregs in PBMCs.<br>Decreased the proportion of Th17 cells.<br>Reduced the levels of pro-inflammatory cytokines such as IFN-γ, TNF-α-α, MCP-1, IL-1β, IL-6, and IL-17A.	Increased the levels of propionate, butyrate, isobutyrate, and isovalerate.<br>Decreased the concentrations of TCDCA and DCA.<br>Increased the levels of isoalloLCA.
Empagliflozin ameliorates atherosclerosis via regulating the intestinal flora	Reduced the *F/B* ratio.<br>Increased the abundance of *Coriobacteriaceae, S24-7* (also known as Muribaculaceae), *Lachnospiraceae, Lactobacillus, Subdoligranulum, Clostridium, Ruminococcaceae, Adlercreutzia, Oscillospira, Coprococcus,* and *Anaerotruncus*.	Decreased the levels of pro-inflammatory cytokines TNF-α and IL-6.<br>Increased the levels of anti-inflammatory cytokines IL-4 and IL-10.	Decreased the levels of CA, LCA, and DCA in fecal BAs.
Ticagrelor combined with aspirin displays the signature of regulating the gut 1microbiome in consistence with improving the immuno-inflammatory response in atherosclerosis	Increased the alpha diversity of the gut microbiota.<br>Increased the abundance of *Parasutterella* and *Muribaculum*.<br>Decreased the abundance of *Firmicutes, Bilophila, Blautia, Clostridium_sensu_stricto_1,* and *[Eubacterium]_fissicatena_group*.	Increased the number of Foxp3+ Tregs, Foxp3+Helios+ Tregs, CD39+ Tregs, and CD73+ Tregs.<br>Decreased the number of IL17A+\CD4+ T helper cells.<br>Upregulated the expression of ADORA2A and ADORA2B genes.<br>Decreased the levels of pro-inflammatory cytokines such as IFN-γ, IL-1β, IL-6, MCP-1, TNF-α, and IL-17A.<br>Increased the level of anti-inflammatory cytokine IL-10.	
Impavido attenuates inflammation, reduces atherosclerosis, and alters gut microbiota in hyperlipidemic mice	Increased the alpha and beta diversity of the gut microbiota.<br>Decreased the abundance of *Romboutsia*.<br>Increased the abundance of *Bacteroides*.	Decreased the plasma level of IL-1β.	Promoted reverse cholesterol transport.
Disulfiram Reduces Atherosclerosis and Enhances Efferocytosis, Autophagy, and	Decreased the abundance of *Romboutsia, Lactococcus*, and *Blautia*.<br>Increased the abundance of *Turicibacter, Bidifobacterium, Lactobacillus,* and *Akkermansia*.	Decreased the serum level of IL-1β.<br>Upregulated the expression of MerTK receptor on THP-1 macrophages.	Upregulated the expression of LDLR and Cyp7a.

Atheroprotective Gut Microbiota in Hyperlipidemic Mice				
Metformin intervention ameliorates AS in ApoE-/- mice through restoring gut dysbiosis and anti-inflammation	Reduced the *F/B* ratio.<br>Decreased the abundance of *Firmicutes, Proteobacteria,* and *Romboutsia.*<br>Increased the abundance of *Akkermansia, Bacteroidetes*, and *Bifidobacterium*.	Reduced the serum level of T-SOD .<br>Reduced the plasma levels of TNF-α and IL-6.	Reduced the plasma levels of LDL-C, TG, and TC.<br>Increased the plasma level of HDL-C.	
Hydroxyurea ameliorates atherosclerosis in ApoE-/mice by potentially modulating Niemann-Pick C1-like 1 protein through the gut microbiota	Reduced the *F/B* ratio.<br>Decreased the abundance of *Lactobacillus* and *Helicobacter.*<br>Increased the abundance of *Lachnospiraceae_NK4A136, Lachnospiraceae_UCG-008, Lachnospiraceae_UCG-006,* and *Roseburia.*		Reduced the serum levels of TC, LDL-C, and ox-LDL.<br>Downregulated the expression of NPC1L1 in small intestinal epithelial cells.	
Cross-omics analysis revealed gut microbiomerelated metabolic pathways underlying atherosclerosis development after antibiotics treatment	Decreased the abundance of *Lachnospiraceae, Ruminococcaceae, Porphyromonadaceae,* and *Prevotellaceae*.		Inhibited secondary bile acid metabolism.	
Reshaping of the gastrointestinal microbiome alters atherosclerotic plaque inflammation resolution in mice	Increased the *F/B* ratio.<br>Decreased the abundance of *Anaeroplasma, Lactobacillus,* and *Bacteroides.*<br>Increased the abundance of *Turicibacter* and *Paenibacillus*.	Increased the number of M1-polarized CD68 foam cells.		
Remodeling Intestinal Microbiota Alleviates Severe Combined Hyperlipidemia-Induced Nonalcoholic Steatohepatitis and Atherosclerosis in LDLR-/- Hamsters	Decreased the plasma level of LPS.		Decreased the plasma levels of TG, HDL-C, and NEFA.<br>Decreased the activity of LPL.<br>Downregulated the expression of lipid absorption-related genes (CD36 and NPC1L1) and transport-related genes (FABP1 and FATP4).	Upregulated the mRNA expression levels of tight junction markers ZO-1, occludin, and Cdh1/5.
Protective effect of hydroxychloroquine on rheumatoid arthritis-associated	Increased the abundance of *Akkermansia* and *Parabacteroides*.<br>Decreased the abundance of *Clostridium sensu stricto cluster.*		Decreased the serum levels of LDL-C, TCHO, and TG.<br>Increased the serum level of HDL-C.	

atherosclerosis			
Inhibition of MAOB Ameliorated High-Fat-Diet-Induced Atherosclerosis by Inhibiting Endothelial Dysfunction and Modulating Gut Microbiota	Increased the abundance of *Faecalibaculum* and *Akkermansia*.<br>Decreased the abundance of *unclassified_f__Lachnospiraceae, Desulfovibrio,* and *Blautia*.	Decreased the plasma levels of ROS and MDA.<br>Downregulated the expression of inflammatory genes such as IL-1β, IL-1α, and IL-6.	Decreased the serum levels of TC, TG, and LDL-C.
Endurance exercise ameliorates Western diet–induced atherosclerosis through modulation of microbiota and its metabolites	Reduced the abundance of *Desulfovibrio, Parabacteroides,* and *Bacteroides*.	Downregulated the expression of VCAM-1, MCP-1, IL-1β, and TNF-α in arterial tissue.	Lowered the serum HDL-C level.<br>Increased the total levels of SCFAs, propionate, and butyrate in the feces.
Time to run: Late rather than early exercise training in mice remodels the gut microbiome and reduces atherosclerosis development	Decreased the alpha diversity of the gut microbiota.<br>Increased the abundance of the *Firmicutes, Ruminococceae*, and *Lachnospiraceae families.*<br>Increased the abundance of SCFA-producing microbial populations, such as *uncultured Ruminococcus species, one uncultured Lachnospiraceae species, Lachnospiracea bacterium 28-4, one uncultured Clostridiales species,* and *Anaerotruncus G*.	Reduced the levels of IL-10, ICAM-1, and VCAM-1 without affecting vascular inflammation.	
Directed remodeling of the mouse gut microbiome inhibits the development of atherosclerosis	Decreased the abundance of *Firmicutes.*<br>Increased the abundance of *Bacteroidetes.*	Decreased the levels of pro-inflammatory cytokines such as IL-6, TNF-α, and IL-1β.	Decreased the plasma levels of TC, TG, VLDL-C, and LDL-C.<br>Increased the levels of SCFAs and bile acids.
Fecal microbiota transplantation ameliorates atherosclerosis in mice with C1q/TNF-related protein 9 genetic deficiency	Increased the relative abundance of *Firmicutes* and *Bacteroidetes*.<br>Decreased the relative abundance of *Proteobacteria* and *Verrucomicrobia*.	Restored the number of macrophages and lymphocytes to normal.	Decreased the levels of TC, TG and LDL-C.<br>Increased the level of HDL-C.<br>Improved the LDL/HDL ratio.

Therapeutic potential of human umbilical cord mesenchymal stem cells on aortic atherosclerotic plaque in a high-fat diet rabbit model	Increased gut microbiota diversity.<br>Increased the abundance of *Ruminococcaceae_UCG-014, Ruminococcus_1, [Eubacterium]_coprostanoligenes_gr.,* and *Christensenellaceae_R-7_group.*<br>Decreased the abundance of *Bacteroides* and *Ruminococcaceae_UCG_00.*	Lowered the levels of macrophage marker CD68 and inflammatory cytokines IL-6 and TNF-α.<br>Increased the levels of anti-inflammatory cytokines IL-10 and TGF-β.<br>Downregulated the expression of the macrophage scavenger receptor SRA-1.	Lowered the serum levels of ALT, TC, LDL-C, TG, CK-MB, and ApoB.<br>Downregulated the expression of the macrophage scavenger receptor CD36.
The role of gut microbiome and its interaction with arsenic exposure in carotid intima-media thickness in a Bangladesh population	Increased the abundance of *Aeromonadaceae* and *Citrobacter*.		
Chronic Intermittent Hypoxia Participates in the Pathogenesis of Atherosclerosis and Perturbs the Formation of Intestinal Microbiota	Decreased the abundance of *Sutterella*.<br>Increased the abundance of *Halomonas, Halomonadaceae, Oceanospirillales, Actinobacteria, Allobaculum, Lactobacillus, Desulfovibrio, Oscillospira, Adlercreutzia, Bacteroides, Helicobacter,* and *Odoribacter*.		
Propamocarb exposure has the potential to accelerate the formation of atherosclerosis in both WT and ApoE−/− mice accompanied by gut microbiota dysbiosis	Increased the abundance of *Peptostreptoccaceae, Ruminococaceae, Clostridiales-VadinBB60_group, Paeniclostridium, Allobaculum,* and *Clostridioides.*<br>Decreased the abundance of *Butyricicoccus, Lachnospiraceae_nk4a136,* and *Roseburia*.	Upregulated the mRNA expression levels of IL-1β, TNF-α, ICAM-1, and VCAM-1 in the aorta.<br>Increased the protein levels of CD36, NF-κB, VCAM-1, and ICAM-1 in the aorta.<br>Increased the serum levels of IL-1α, IL-1β, IFN-γ, and TNF-α.	Increased the serum levels of TC, TG, and LDL-C.<br>Decreased the serum level of HDL-C.<br>Upregulated the mRNA expression levels of HMG-CoA synthase, LDL-R, and SR-B1 in the liver.
Diesel exhaust particles alter the profile and function of the gut microbiota upon subchronic oral administration in mice	Decreased the alpha diversity of the gut microbiota.<br>Decreased the abundance of *Proteobacteria, Patescibacteria,* and *Sutterellaceae.*<br>Increased the abundance of *Campylobacteria, Cyanobacteria, Helicobacteraceae, Roseburia, Helicobacter,* and *Rikenellaceae_RC9_gut_group*.		Lowered the plasma triglyceride level.<br>Reduced the plasma SCFAs level.
Clock-Bmal1 mediates MMP9 induction in acrolein-promoted atherosclerosis associated with gut microbiota regulation	Increased the abundance of *Firmicutes* and *Deferribacteres.*<br>Decreased the abundance of *Bacteroidetes* and *Actinobacteria.*	Lowered the mRNA expression of Clock and Bmal.<br>Increased the protein level of MMP9.	

CRISPR/Cas9 based blockade of IL-10 signaling impairs lipid and tissue homeostasis to accelerate atherosclerosis	Increased the abundance of *Proteobacteria, Helicobacter,* and *Staphylococcus*.<br>Decreased the abundance of *Firmicutes, Bacteroidetes, Lactobacillus, Allobaculum*, and *Erypelotrichia*.<br>Increased the plasma LPS level.	Increased the number of WBC, MO, and GR.<br>Upregulated the mRNA expression levels of IL-1b, IL-6, NFkB, and TNFa in the spleen.<br>Promoted colon inflammatory cell and macrophage infiltration.<br>Upregulated the mRNA expression of BAX, MLKL, and MYD88 in white adipose tissue.<br>Upregulated expression of BAX, MLKL, and MYD88 genes in white adipose tissue, and downregulated expression of BCL2 gene in white adipose tissue.<br>Promoted LPS-induced upregulation of TLR4, MYD88, and MLKL gene expression in the liver.	Lowered the plasma HDL-C level.<br>Increased the plasma levels of ApoB100 and ApoB48.<br>Decreased the plasma level of ApoA.<br>Increased the plasma VLDL-C levels.	
Influence of Intermittent Hypoxia/ Hypercapnia on Atherosclerosis, Gut Microbiome, and Metabolome	Increased the abundance of *Lachnospiraceae_NK4A136_group, Acetatifactor, Lachnospiraceae_bacterium_A4,Lactobacillus_animalis,Parabacteroides_goldsteinii, Lachnospiraceae_NK4A136_group, Mucispirillum,* and *Ruminococcaceae_UCG-01*<br>Increased the serum LPS level.	Increased the serum levels of inflammatory cytokines IL-6 and TNF-α.<br>LPS promoted a significant increase in the levels of inflammatory cytokines IL-6, IL-1b, and TNF-α in the liver.<br>LPS promoted an increase in the mRNA expression of FMO3 in the liver, thereby increasing TMAO levels.		Downregulated the mRNA expression of tight junction proteins ZO-1, Claudin-1, and Occludin.

TMAO: Trimethylamine N-Oxide，F4/80: EGF-like Module-Containing Mucin-Like Hormone Receptor-Like 1，LPS: Lipopolysaccharide，MCP1: Monocyte Chemoattractant Protein-1, VCAM1: Vascular Cell Adhesion Molecule 1，CD4: Cluster of Differentiation 4, CD86: Cluster of Differentiation 86，Foxp3: Forkhead Box P3，oxLDL: Oxidized Low-Density Lipoprotein，MDA: Malondialdehyde, SOD: Superoxide Dismutase, TNF-α: Tumor Necrosis Factor α, IL-1β: Interleukin 1 β, MYD88: Myeloid Differentiation Primary Response 88, NFκB: Nuclear Factor Kappa-Light-Chain-Enhancer of Activated B Cells, NF-κB p65: Nuclear Factor Kappa-Light-Chain-Enhancer of Activated B Cells p65, IL-6: Interleukin 6, MCP-1: Monocyte Chemoattractant Protein-1, NO: Nitric Oxide, ICAM-1: Intercellular Adhesion Molecule 1, INOS: Inducible Nitric Oxide Synthase, GSH-PX: Glutathione Peroxidase, MDA: Malondialdehyde, GSH-Px: Glutathione Peroxidase, NLRP3: NLR Family Pyrin Domain Containing 3, IL-18: Interleukin 18, IL-17A: Interleukin 17A, Ccl4: Chemokine (C-C Motif) Ligand 4, Ccl2: Chemokine (C-C Motif) Ligand 2, IL-1b: Interleukin 1b, AKT: Protein Kinase B , T-AOC: Total Antioxidant Capacity, CD68: Cluster of Differentiation 68, SAA: Serum Amyloid A, VEGF: Vascular Endothelial Growth Factor, IL-16: Interleukin 16 , EPO: Erythropoietin, MyD88: Myeloid Differentiation Primary Response 88, TRIF: TIR-Domain-Containing Adapter-Inducing Interferon-β，iNOS: Inducible Nitric Oxide Synthase, eNOS: Endothelial Nitric Oxide Synthase, GLP-1: Glucagon-Like Peptide-1, GPR43: G Protein-Coupled Receptor 43, Ang II: Angiotensin II, IL-3: Interleukin 3, LIF: Leukemia Inhibitory Factor, ERK1/2: Extracellular Signal-Regulated Kinase 1/2, IL-12: Interleukin 12, PPARα: Peroxisome Proliferator-Activated Receptor α, CAT: Catalase, IL-7: Interleukin 7, TNF-β: Tumor Necrosis Factor β, α-SMA: α-Smooth Muscle Actin, SR-a: Scavenger Receptor Class A, NLRP: NLR Family Pyrin Domain Containing, ABCA1: ATP Binding Cassette Subfamily A Member 1, hs-CRP: High-Sensitivity C-Reactive Protein, CCRA: Chemokine (C-C Motif) Receptor, JNK: c-Jun N-Terminal Kinase, OPN: Osteopontin, LOX: Lysyl Oxidase, ET-1: Endothelin-1, MMP-2: Matrix Metallopeptidase 2, MMP-9: Matrix Metallopeptidase 9, ADPN: Adiponectin, GSTM1: Glutathione S-Transferase Mu 1, RAGE: Receptor for Advanced Glycation Endproducts, VEGFA: Vascular Endothelial Growth Factor A, CDKN1A: Cyclin-Dependent Kinase Inhibitor 1A, AGE: Advanced Glycation Endproducts, G-CSF: Granulocyte Colony-Stimulating Factor, KC: Keratinocyte Chemoattractant, MIP-1b: Macrophage Inflammatory Protein-1 b, PBMCs: Peripheral Blood Mononuclear Cells, CD39+ Tregs: CD39-Positive Regulatory T Cells, CD73+ Tregs: CD73-Positive Regulatory T Cells, ADORA2A: Adenosine A2a Receptor, ADORA2B: Adenosine A2b Receptor, SRA-1: Scavenger Receptor Class A Member 1, TGF-β: Transforming Growth Factor β, WBC: White Blood Cells, MO: Monocytes, GR: Granulocytes, MLKL: Mixed Lineage Kinase Domain-Like, TG: Triglycerides, LDL-C: Low-Density Lipoprotein Cholesterol, NR4A: Nuclear Receptor Subfamily 4 Group A, FXR: Farnesoid X Receptor, HDL-C: High-Density Lipoprotein Cholesterol, LDL: Low-Density Lipoprotein, ZO-1: Zonula Occludens-1, CYP7A1: Cytochrome, P450 Family 7 Subfamily A Member 1, Cyp7a1: Cytochrome P450 Family 7 Subfamily A Member 1, Abcg5: ATP-Binding Cassette Subfamily G Member 5, Abcg8: ATP-Binding Cassette Subfamily G

Member 8, CDCA: Chenodeoxycholic Acid, RCT: Reverse Cholesterol Transport, ox-LD: Oxidized Low-Density Lipoprotein, ALC: Acyl-CoA, Sr-b1: Scavenger Receptor Class B Type 1, Abcg5: ATP-Binding Cassette Subfamily G Member 5, Abcg8: ATP-Binding Cassette Subfamily G Member 8, Srbi: Scavenger Receptor Class B Type 1, Abca1: ATP Binding Cassette Subfamily A Member 1, HMGCR: 3-Hydroxy-3-Methylglutaryl-CoA Reductase, SR-BI: Scavenger Receptor Class B Type 1, LXR: Liver X Receptor, ABCG1: ATP Binding Cassette Subfamily G Member 1, CER: Ceramide, LPC: Lysophosphatidylcholine, VLDL: Very Low-Density Lipoprotein, non-HDL-C: Non-High-Density Lipoprotein Cholesterol, HMG-CoA-R: 3-Hydroxy-3-Methylglutaryl-CoA Reductase, LDL-R: Low-Density Lipoprotein Receptor, eWAT: Epididymal White Adipose Tissue, SREBP-1: Sterol Regulatory Element-Binding Protein 1, ACC: Acetyl-CoA Carboxylase, FAS: Fatty Acid Synthase, C/EBPα:CCAAT/Enhancer-Binding Protein α, PPARγ: Peroxisome Proliferator-Activated Receptor Gamma, LXR-α: Liver X Receptor α, SERBP: Sterol Regulatory Element-Binding Protein, p-AMPK: Phosphorylated AMP-Activated Protein Kinase, AMPK: AMP-Activated Protein Kinase, NPC1L1: Niemann-Pick C1-Like 1, ACAT2: Acyl-CoA Cholesterol, Acyltransferase 2, MTTP: Microsomal Triglyceride Transfer Protein, Cyp27a1: Cytochrome P450 Family 27 Subfamily A Member 1, Cyp7b1: Cytochrome P450 Family 7 Subfamily B Member 1, HMGB1: High Mobility Group Box 1, HCAECs: Human Coronary Artery Endothelial Cells, TUDCA: Tauroursodeoxycholic Acid, APOA1: Apolipoprotein A1, SFAs: Saturated Fatty Acids, MUFAs: Monounsaturated Fatty Acids, PUFAs: Polyunsaturated Fatty Acids, β-MCA: β-Muricholic Acid, α-MCA: α-Muricholic Acid, AMPKα: AMP-Activated Protein Kinase α, BA: Bile Acid, LPC 18:1: Lysophosphatidylcholine 18:1, LPA 18:1: Lysophosphatidic Acid 18:1, LPA 18:2: Lysophosphatidic Acid 18:2, LPA 20:4: Lysophosphatidic Acid 20:4, PEIPC: Phosphatidylethanolamine Isopropanolamine, POVPC: Phosphatidylcholine Oxidized at the Vinyl Ether Group, PGPC: Phosphatidylcholine Oxidized at the Glycerol Backbone, ACC1: Acetyl-CoA Carboxylase 1, CYP27A1: Cytochrome P450 Family 27 Subfamily A Member 1, LCA-3S: Lithocholic Acid 3-Sulfate, CE: Cholesterol Esters, DCA: Deoxycholic Acid, NEFA: Non-Esterified Fatty Acids, CK-MB: Creatine Kinase-MB, ApoB: Apolipoprotein B, ApoB100: Apolipoprotein B100, ApoB48: Apolipoprotein B48, ApoA: Apolipoprotein A.

Supplementary Table 9: This table presents information related to the translation of clinical trials.

Title	Drug/Biomarker	Clinical trial deficiencies	Solutions
Gut Microbiota Functional Dysbiosis Relates to Individual Diet in Subclinical Carotid Atherosclerosis	*Escherichia coli, Faecalibacterium prausnitzii,* TMAO, Zonulin	1.Limitations of self-reported dietary data: Reliance on self-reported dietary habits may introduce recall bias and inaccuracies, potentially compromising the validity of the observed associations. This methodological constraint hinders the definitive establishment of causal relationships between dietary patterns and gut microbiota composition.<br>2.Confounding factor considerations: Advanced age in patients with SCA emerges as a predominant confounding variable, potentially influencing both microbial profiles and cardiovascular parameters. Furthermore, incomplete adjustment for pharmaceutical interventions (particularly lipid-lowering medications) leaves residual confounding in microbiota-drug interaction analyses.<br>3.Sample selection biases: The study cohort demonstrated notable demographic constraints, including (a) restricted age distribution (60-80 years) with underrepresentation of younger populations and (b) predominant recruitment of lean/overweight individuals, limiting generalizability across broader BMI categories. Geographical constraints (single-center sampling) further restrict extrapolation to diverse ethnic groups.<br>4.Mechanistic research gaps: Although Escherichia coli was identified as a key TMAO producer, the study lacked mechanistic validation through microbial transplantation or gene knockout models. The physiological significance of normal zonulin levels in early atherosclerosis remains undetermined, necessitating investigations into alternative intestinal permeability biomarkers.<br>5.Clinical translation challenges: Current findings primarily describe subclinical associations, necessitating (a) longitudinal validation in atherosclerotic progression cohorts and (b) therapeutic intervention trials to assess microbiota modulation efficacy. The absence of large-scale population data prevents a robust evaluation of the predictive value of microbial signatures for cardiovascular events.	1. Improvement of Data Collection Methods: Employ smartphone health applications or digital tools to record dietary data in real time, minimizing self-reporting bias, and incorporate objective indicators (e.g., wearable devices) to monitor lifestyle parameters.<br>2. Expansion of the Research Scope: Enrolling additional young participants to explore gut microbiota characteristics across age groups and enhance the geographic diversity of cohorts to ensure the universal applicability of findings.<br>3. Mechanistic Investigation: Validate the Escherichia coli TMAO interaction mechanism through in vitro experiments and animal models and elucidate the biological implications of normal zonulin levels and their potential role in early stage atherosclerosis (AS) development.<br>4. Personalized Intervention Strategies: Develop microbiota profile-based personalized dietary regimens and conduct intervention trials combining probiotics and prebiotics to assess their effectiveness in subclinical atherosclerosis (SCA) prevention.<br>5. Multicenter Collaborative Research: Implement multicenter clinical trials to verify gut microbiota alterations in cardiovascular disease progression and systematically investigate the specific impacts of pharmaceutical interventions on microbial communities to optimize therapeutic strategies.
Long-chain monounsaturated fatty acids improve endothelial function with altering microbial flora	Fish oil-derived long-chain monounsaturated fatty acids (LCMUFAs)	1. No significant changes in blood lipid profiles: Although LCMUFA improved flow-mediated dilation (FMD), it failed to significantly affect blood lipid levels, such as low-density lipoprotein (LDL) cholesterol. The study cohort consisted of healthy individuals whose baseline lipid levels were within the normal range, leaving limited room for further reduction. Moreover, the majority of participants were female, and potential sex differences or menstrual cycle effects were not systematically evaluated in this study.<br>2. Short trial duration: The 8-week intervention period might have been	1. Extend the trial duration: Increase the intervention period (e.g., 12 weeks or longer) to evaluate the long-term effects of LCMUFA on lipid profiles and atherosclerosis development.<br>2. Include patient populations: Repeat the experiment in patients with clinically confirmed atherosclerosis to verify whether LCMUFA improves endothelial function and reduces cardiovascular risks.<br>3. Collect fecal samples: Analyze dynamic changes in the gut microbiota of participants to clarify the specific regulatory mechanisms of LCMUFA on microbial metabolites (e.g., SCFAs).<br>4. Test dose-response relationships: A multi-dose group experiment was designed to determine the optimal LCMUFA dosage and corresponding therapeutic efficacy.

		insufficient to detect changes in LDL cholesterol levels, as alterations in lipid metabolism often require longer observation windows to manifest.<br>3. Absence of fecal sample analysis: The study did not collect fecal specimens from participants, precluding the direct validation of the changes in the gut microbiota composition that were hypothesized to mediate the observed endothelial functional improvements.<br>4. Lack of dose-response assessment: The experimental design did not investigate potential dose-dependent relationships between LCMUFA intake and its effects on endothelial function and microbial community dynamics.<br>5. Limited generalizability: The findings were exclusively derived from healthy volunteers, leaving the therapeutic efficacy of LCMUFA in atherosclerotic populations untested and clinically relevant endpoints unaddressed.	5.Consider sexual dimorphism: Enroll more male participants and analyze the effects of sex and estrous cycles on LCMUFA's biological effects of LCMUFAs.<br>6. Integrate animal and human studies: Utilize established animal models (e.g., ApoE knockout mice) to investigate the mechanisms of LCMUFA action in-depth, followed by a comparative analysis with human experimental data.
Metagenomic analysis of the gut microbiome in atherosclerosis patients identify cross-cohort microbial signatures and potential therapeutic target	*Streptococcus anginosus, Atopobium parvulum* , *Actinomyces graevenitzii*	1. Sample heterogeneity: The study involved cohorts from Sweden and China, where geographical disparities led to significant differences in microbial community composition. This geographical variation may compromise the generalizability of the research findings.<br>2. Limitations of functional pathway analysis: Although altered pathways related to starch degradation and folate transformation were identified, the specific mechanisms underlying these pathways and their causal relationships with atherosclerosis require further validation.<br>3. Interference from medication effects: LDL, HDL, and cholesterol levels are significantly influenced by medication. Sole reliance on control group data analysis may inadequately represent the clinical profiles of patients. Insufficient biomarker specificity: Certain marker species (e.g., *Streptococcus anginosus*) are associated not only with atherosclerosis but also with other pathologies, including liver cirrhosis and gastric cancer, necessitating further refinement of diagnostic specificity.<br>4. Strain-level adaptive divergence: Functional variations may exist among bacterial strains from different geographical regions, complicating cross-regional clinical applications	1. Expand sample diversity: Enroll cohorts from broader geographic regions to minimize the influence of geographic variation on research outcomes.<br>2. Elucidation of functional mechanisms: Validation of the functional roles of key pathways through in vitro experiments and animal models to establish causal relationships in atherosclerosis pathogenesis.<br>3. Control pharmaceutical interference: Collect comprehensive medication records for statistical adjustments of drug-induced metabolic alterations or preferentially analyze treatment-naïve patients.<br>4. Enhancing biomarker specificity: Integration of multi-omics data (e.g., transcriptomic and metabolomic profiles) to identify biomarkers with improved disease specificity.<br>5. Conduct strain-level characterization: Perform large-scale strain-resolved analyses to clarify functional heterogeneity across microbial strains, facilitating the development of strain-based personalized therapeutic strategies.<br>6. Validation through clinical trials: Randomized controlled trials (RCTs) should be designed to systematically evaluate the preventive and therapeutic efficacy of candidate probiotics against AS progression.
Ticagrelor combined with aspirin displays the signature of regulating the gut microbiome in consistence with improving the immuno-inflammatory response in atherosclerosis	Ticagrelor + Aspirin (TA) , Clopidogrel + Aspirin (CA)	1. Insufficient sample size: The study enrolled only 21 patients (11 in the TA group and 10 in the CA group), potentially leading to insufficient statistical power. The small sample size limits evaluation of long-term efficacy and safety.<br>Rationale: Maintains causality between sample size and statistical limitations while adhering to precise clinical trial terminology.<br>2. Lack of long-term follow-up data: The study conducted only one-month follow-up, preventing comprehensive assessment of ticagrelor's sustained therapeutic effects and potential side effects over extended periods.	1. Expand sample size: Increase the number of study participants to enhance statistical power and validate the universality of the preliminary findings.<br>2. Extended follow-up duration: Conduct multicenter, long-term randomized controlled trials to evaluate the long-term efficacy and safety profile of ticagrelor across diverse populations.<br>3. Investigate underlying mechanisms: Utilize single-cell sequencing and metabolomics technologies to elucidate the precise regulatory mechanisms of ticagrelor on the gut microbiota and its interaction with the immune system.<br>4. Control confounding factors: Strictly exclude antibiotic usage or other potential microbiota-altering interventions in future studies to ensure the reliability of the results.<br>5. Develop novel biomarkers: Explore more sensitive and specific biological markers for the precise

		Rationale: Emphasizes temporal limitation ("one-month") and critical missing data categories (both efficacy and safety).<br>3. Complexity of mechanisms: The interaction mechanisms between gut microbiota and the immune system remain incompletely elucidated, particularly regarding how ticagrelor specifically regulates functional pathways of microbial communities.<br>Rationale: Uses "incompletely elucidated" to denote ongoing scientific uncertainty while specifying the knowledge gap in pathway regulation.<br>4. Antibiotic interference: Antibiotic depletion experiments demonstrated ticagrelor's effects depend on gut microbiota, which may obscure other potential mechanisms (e.g., direct immune-modulatory effects).<br>Rationale: Preserves experimental causality ("depletion experiments demonstrated") and explicitly lists alternative mechanisms in parentheses as per academic conventions.<br>5. Inadequate sensitivity and specificity of biomarkers: Currently employed biomarkers (e.g., PGN biosynthesis pathways, Tregs expression) may exhibit individual variations, potentially compromising result interpretation.	assessment of ticagrelor's therapeutic effects and prediction of patient responses.<br>6. Personalized treatment strategies: Formulate individualized therapeutic regimens based on patients' gut microbiota profiles to maximize ticagrelor efficacy while minimizing adverse reactions.
Faecalibacterium prausnitzii as a potential Antiatherosclerotic microbe	*Faecalibacterium_prausnitzii*, *Lachnospiraceae_bacterium_1_1_57FAA*, *Dorea_longicatena*, *Eubacterium_ramulus*	1. Sample selection bias: The study recruited participants from specific regions, which may have introduced region-specific microbial profile characteristics, compromising the generalizability of the findings to broader populations.<br>2. Multifactorial confounding: Table 1 shows significant differences in the participants' baseline characteristics (e.g., smoking status, alcohol consumption, and comorbidities). These covariates may independently influence the gut microbiota composition, thereby confounding the observed associations between gut microbiota alterations and CAD progression.<br>3. Insufficient mechanistic investigation: Although experimental data suggest that *Faecalibacterium prausnitzii* reduces atherosclerosis risk by suppressing LPS synthesis and enhancing intestinal barrier integrity, the precise molecular mechanisms underlying these protective effects remain unclear.<br>4. Lack of longitudinal observation: The cross-sectional design predominantly employed in this study did not provide long-term follow-up data to validate the sustained preventive efficacy of *F. prausnitzii* against clinical CAD endpoints.<br>5. Unclear intervention parameters: The manuscript fails to specify the therapeutic dosage and administration frequency of *F. prausnitzii* supplementation, which hinders its translation into clinical practice and reproducibility in subsequent trials.	1. Expanding sample size and diversity: Future studies should include participants from diverse regions and ethnicities to minimize geographic and individual biases in the analysis.<br>2. Controlling for confounding variables: More rigorous adjustments are required to account for the effects of confounding factors such as smoking, alcohol consumption, and medication use, thereby clarifying the causal relationship between gut microbiota alterations and coronary artery disease (CAD).<br>3. Mechanistic investigations: Integrated multi-omics approaches (e.g., genomics and transcriptomics) should be employed to elucidate the molecular mechanisms by which *F. prausnitzii* reduces lipopolysaccharide (LPS) biosynthesis, which will inform the development of targeted therapeutic strategies for IBD.<br>4. Longitudinal study design: Prospective cohort studies with extended follow-up periods are needed to assess the temporal impact of *F. prausnitzii* abundance on CAD progression and to validate its long-term preventive efficacy.<br>5. Intervention protocol optimization: Systematic dose-response studies must be conducted to determine the optimal therapeutic dosage and administration frequency of *F. prausnitzii*, accompanied by randomized controlled trials to evaluate clinical safety and treatment outcomes.

Supplementary Table 10: This table presents information related to the TMAO and SCFAs in AS.

	TMAO	SCFAs
Sources	Trimethylamine (TMA) is a low-boiling nitrogen-containing small-molecule chemical. Genetically modified TMA lyases convert dietary precursors such as choline, phosphatidylcholine, and l-carnitine, which are found in meats, dairy products, eggs, and fish, into TMA. Following intestinal absorption, TMA travels through the portal vein to the liver, where it is transformed to TMAO by the flavin-containing monooxygenase, and microorganisms participate in this process.	SCFAs, which comprise acetates, butyrates, and propionates, are mostly produced when anaerobic bacteria breakdown undigested dietary fiber in the large intestine and cecum. SCFAs are created by several Gut Microbiota metabolisms; for example, Bacteroidetes make acetic and propionic acids, whereas Firmicutes produce butyric acid.
Roles	Although the role of TMAO in the pathogenesis of AS is not singular, current research suggests that TMAO contributes to the progress of AS.	SCFAs are involved in the regulation of AS through multiple pathways and the potential molecular mechanisms of SCFAs warrant further in-depth investigation.

Supplementary Materials 2: The call-out box summarizing the most promising interventions.

- ❑ **Probiotics and prebiotics**
- ❑ **Diet**
- ❑ **Natural compounds**
- ❑ **Traditional Chinese Medicine**
- ❑ **Western medicines**
- ❑ **Exercise**
- ❑ **Other factors(e.g., Fecal microbiota transplantation and Human umbilical cord mesenchymal stem cells）**
